# Catalyst-free synthesis of 1,2,3-triazole-N-oxide derivatives using *tert*-butyl nitrite: a novel strategy and synthetic applications†

**DOI:** 10.1039/d5ra01327e

**Published:** 2025-04-04

**Authors:** Karuppaiah Perumal, Markabandhu Shanthi, Vijayakumar Hemamalini, Bhaskaran Shankar, Subburethinam Ramesh

**Affiliations:** a Department of Chemistry, School of Chemical and Biotechnology, SASTRA Deemed University Thanjavur 613 401 Tamil Nadu India rameshsbdu@gmail.com; b Department of Chemistry, Thiagarajar College of Engineering Madurai 625 015 Tamil Nadu India

## Abstract

A simple, metal-free method has been developed to synthesize novel 1,2,3-triazole-N-oxide derivatives. In this reaction, *t*-BuONO serves as a NO source, with environmentally friendly solvents such as EtOH and H_2_O employed as additives. Control experiments provided valuable insights into the reaction mechanism. Furthermore, 1,2,3-triazole-N-oxides demonstrated versatility in synthetic transformations.

## Abstract 2

*t*BuONO-mediated synthesis of triazole-N-oxide using *t*BuONO as an NO source. EtOH/H_2_O serves as a green solvent and additive. Triazole N-oxide is used in various synthetic transformations.

## Graphical abstract

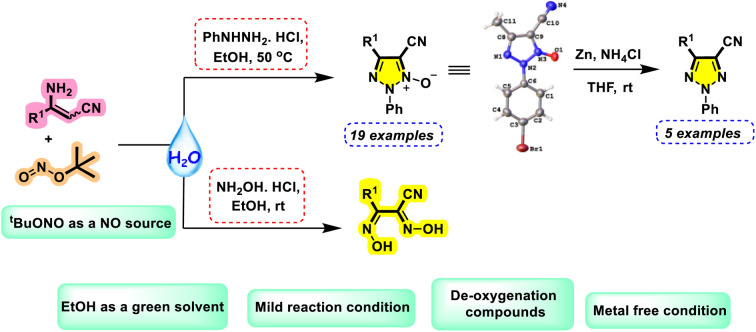

Nitrogen-containing heterocyclic compounds such as furazan, triazole, and tetrazine have been explored as high-energy materials replacing conventional detonators.^1^ Energetic materials are an essential class of compounds widely used in various industries, most notably the military and space technology.^2^ The NO functional group is replacing the typical nitro-energetic group to improve the energy density and oxygen balance. Wang *et al.* demonstrated the disturbance in the symmetry of the compound; that the presence of an N–O bond in a system reduces the length of the C–C bond, increases the C–N bond length in the ring, and facilitates a compound to be an energy material.^3^ The N–O bond in heterocyclic N-oxides confers unique electronic properties, making the nitrogen centre electron-rich and highly polarizable. This dual electron-donating and accepting capacity enables a wide range of synthetic transformations, making N-oxide derivatives indispensable in organic synthesis.^4^ 1,2,3-Triazoles are significant heterocyclic compounds.^5^ They are widely utilized in pharmaceuticals and agrochemicals. Triazoles have been shown to serve a variety of biological activities in Fig. 1.^6^ Heterocyclic N-oxides’ biological activity is related to their distinct electronic characteristics, which allow these molecules to interact efficiently with biological targets. These compounds have proven noteworthy therapeutic promise, with activities that include anticancer,^7^ antibacterial,^8^ antihypertensive,^9^ antiparasitic,^10^ anti-HIV,^11^ and anti-inflammatory properties.^12^ To introduce an N–O bond in a nitrogen heterocycle, one must utilize appropriate oxidizing agents, which must provide proper chemo and regioselectivity, which is very difficult to control when multiple nitrogens are available in a system. On the other hand, these oxidizing agents produce unwanted by-products. Designing a methodology that generates an N–O bond during the construction of the ring would avoid these problems. In this context, *tert*-butyl nitrite (TBN), a metal-free reagent, has lately emerged as an efficient reagent in various processes for constructing N-containing complex compounds.^13^ We focused on TBN, which, in general, is utilized for *in situ* generation of diazonium salt and limitedly utilized as a NO source.

**Fig. 1:**
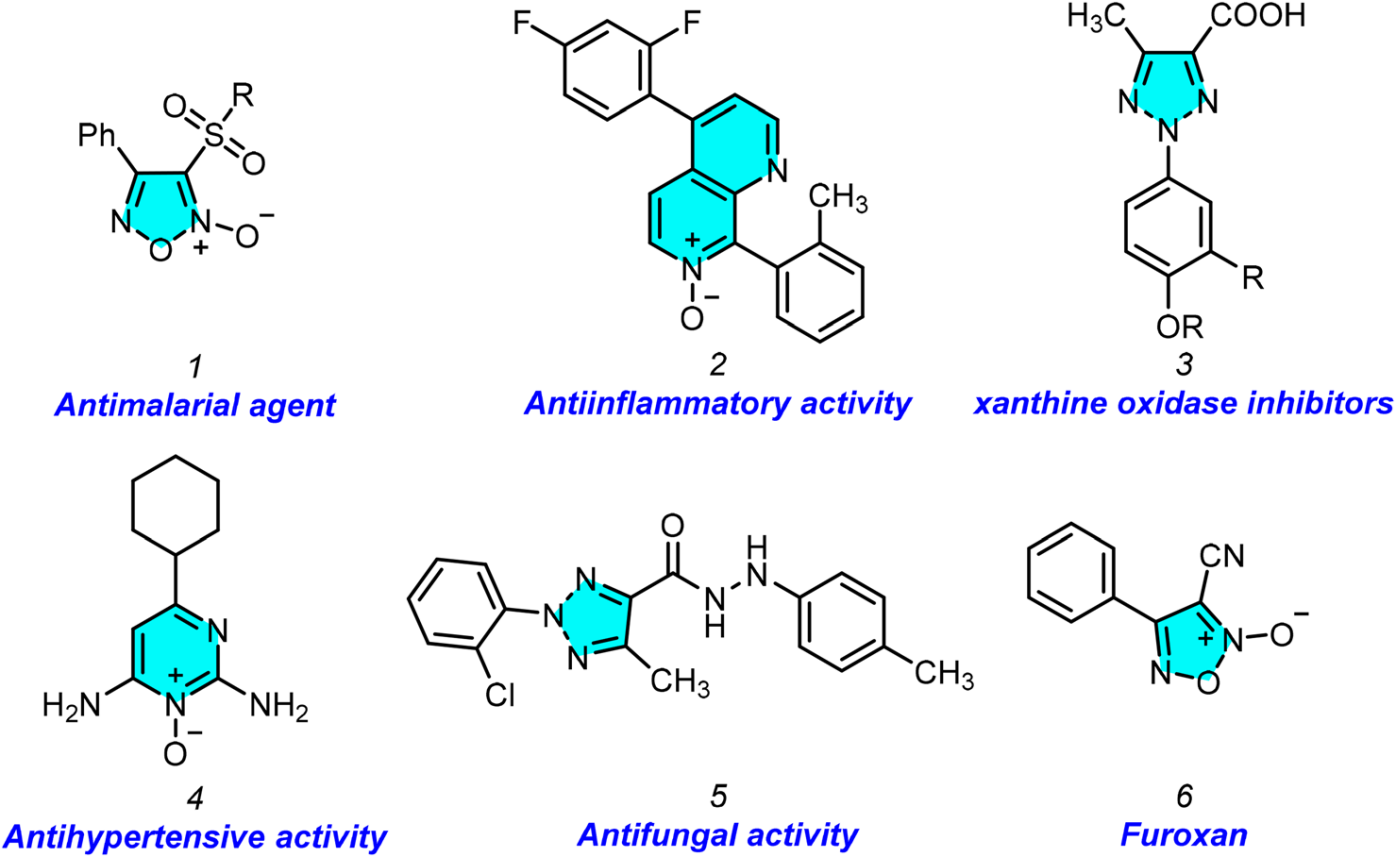
Biologically active N-oxide and triazole compounds.

If a structure design facilitates capturing the NO group from TBN and following cyclization with an electrophilic nitrogen atom in NO, we could achieve the heterocyclic N-oxide derivative in one pot. While several techniques are available to synthesize heterocyclic N-oxides, triazole N-oxide synthesis has received little attention.

Ethanol has emerged as an effective and sustainable green solvent for various organic processes, driven by growing global environmental concerns and principles of green chemistry.^14^ When compared to traditional solvents, ethanol stands out due to its affordability, environmental benignity, and renewable nature, making it a preferred choice in many applications. Additionally, water is often used as an additive, further enhancing the eco-friendliness of the process. On the other hand, catalysts, while often essential in facilitating chemical reactions, can present significant challenges. They tend to be expensive and environmentally hazardous, and metal catalysts are often toxic. Additionally, their removal from reaction mixtures can be difficult, especially in large-scale industrial processes. The use of catalysts can also lead to the formation of harmful by-products, which complicates the overall sustainability of the process. In contrast, catalyst-free reactions have gained significant attention as a viable alternative, offering a more cost-effective and environmentally friendly approach for synthesizing organic molecules. These reactions typically feature reduced sensitivity to air and moisture, minimize side reactions, and promote cleaner, more efficient product separation. Furthermore, the simplicity of operating catalyst-free systems enhances their appeal, making them a promising option for green, large-scale synthesis with minimal environmental impact.^15^

In this context, some heterocyclic N-oxides have been reported using TBN, metal, and high temperature.^16^ A synthesis using an electrochemical method was published in the Green Chemistry journal.^17^ Muthu Krishnan *et al.* reported N-oxide synthesis.^18^ however, they utilize acetoacetate under oxidative conditions using copper metal-based transformation for the synthesis of triazole N-oxide only (Scheme 1). In addition, our paper demonstrated triazole-N-oxide has been synthesis without additional catalysts or any metal under mild reaction conditions.

**Scheme 1:**
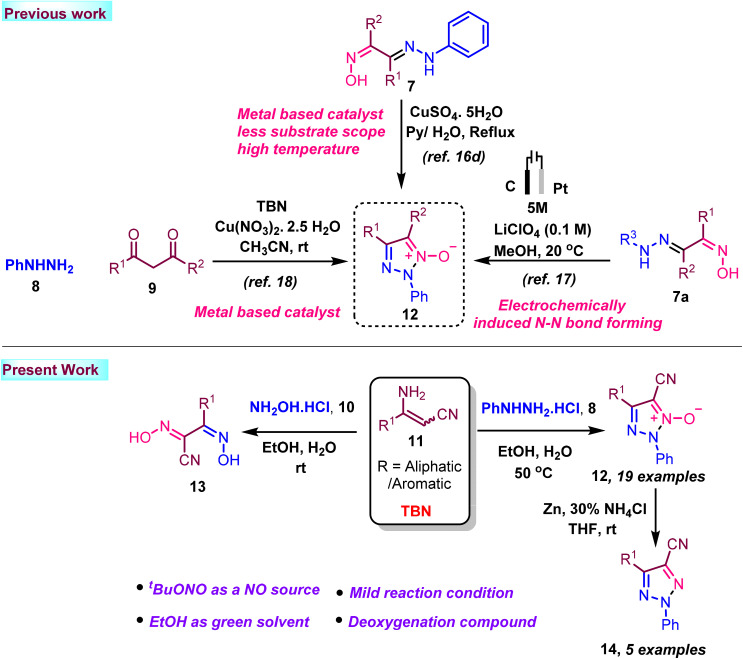
Various synthetic methodology of triazole-N-Oxide.

## Results and discussion

The investigation on synthesizing our desired compound 12a began with optimization of the reaction conditions using phenylhydrazine hydrochloride (8a), and 3-aminocrotononitrile (11a), as the model substrate. Initially, we used *tert*-butyl nitrite as a NO source, H_2_O (2 mmol) as an additive, and DMSO as the solvent, under open-air conditions. The expected triazole-N-oxide product 12a was obtained in 18% of the yield (Table 1, entry 1). ^1^H,^13^C NMR, HRMS and X-ray crystallographic method analysis confirmed the structure of the compound 12b.^19^

**Table 1:** Optimization of reaction condition

S. no.	Nitrating source	Solvent	Yield^a^ (%)
1^b^	TBN, 3 equiv.	DMSO	18
2^c^	TBN, 3 equiv.	DMSO	39
3	TBN, 3 equiv.	1,4-Dioxane	38
4	TBN, 3 equiv.	n-propanol	49
5	TBN, 3 equiv.	MeOH	45
6	TBN, 3 equiv.	DCM	31
7	TBN, 3 equiv.	DMF	45
8	TBN, 3 equiv.	CH_3_NO_2_	47
9	TBN, 3 equiv.	ACN	40
**10**	**TBN, 3 equiv.**	**EtOH**	**85**
11^d^	TBN, 3 equiv.	EtOH	30
12^e^	TBN, 3 equiv.	EtOH	ND
13^f^	TBN, 3 equiv.	EtOH	ND
14	TBN, 2 equiv.	EtOH	29
15	TBN, 4 equiv.	EtOH	63
16^g^	TBN, 3 equiv.	EtOH	ND
17	^*n*^BuONO, 3 equiv.	EtOH	50
18	^i^AmylONO, 3 equiv.	EtOH	50
19^h^	TBN, 3 equiv.	EtOH	74
20^i^	TBN, 3 equiv.	EtOH	ND
21^j^	TBN, 3 equiv.	EtOH	ND

a Reaction condition: Isolated yield.

b Open condition.

c Closed condition.

d 1 : 1.

e 1 : 2.

f 2 : 1.

g Without ^*t*^BuONO.

h H_2_O (3 mmol).

i H_2_O (1 mmol).

j H_2_O (4 mmol).

**Scheme 2:**
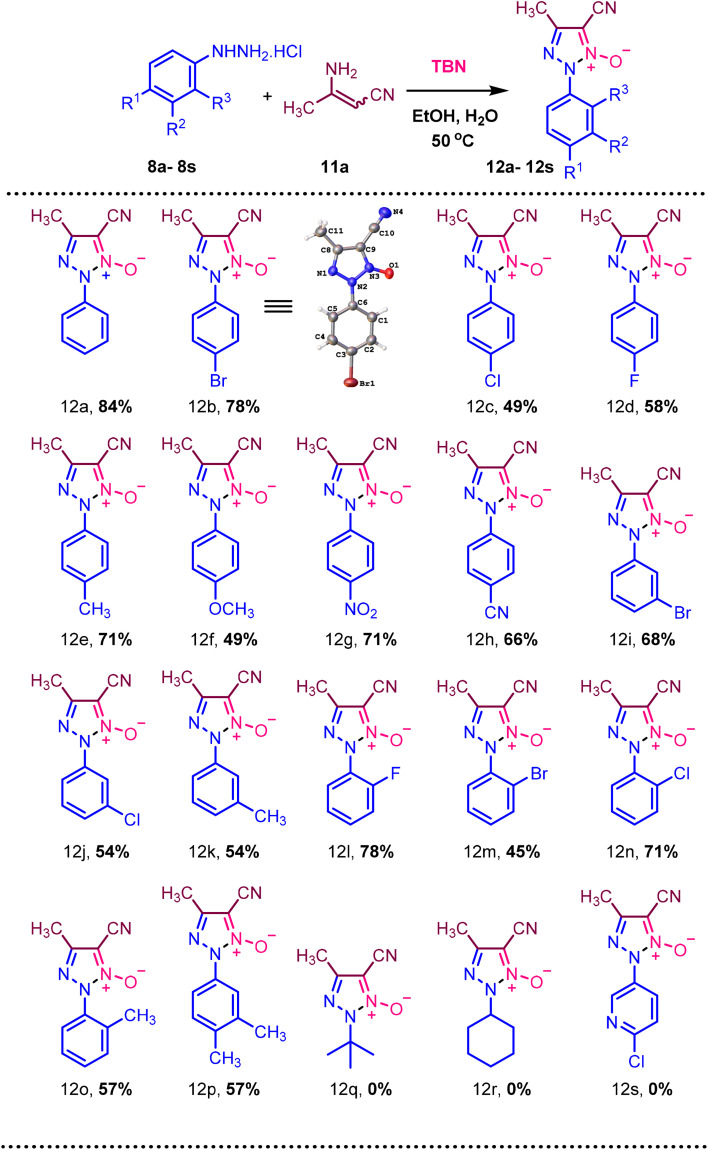
Synthesis of various 5-cyano-4-methyl-2-phenyl-2*H*-1,2,3-triazole 1-oxide (12a–12s) using various phenylhydrazine hydrochloride (8a–8s). ^a^ Reaction condition: 8a (1 mmol), 11a (1.5 mmol), ^*t*^BuONO (3 mmol), H_2_O (2 mmol), EtOH, 50 °C heating. Isolated yields are provided.

The yield of product 12a increased when the reaction was performed in closed conditions (Table 1, entry 2). Notable conversion and yield were achieved while using EtOH as a solvent. Other solvents like 1,4-dioxane, *n*-propanol, MeOH, ACN, DMF, and CH_3_NO_2_ did not improve the yield (Table 1, entries 3–10). Different ratios of starting materials, 8a and 11a, were tested. However, the yield did not improve (Table 1, entries 11–13). When the *tert*-butyl nitrite ratio was changed, the yield did not improve and without *tert*-butyl nitrite the product was not formed (Table 1, entries 14–16). Among various nitrating agents such as *tert*-butyl nitrite, isoamyl nitrite, and *n*-butyl nitrite, *tert*-butyl nitrite was superior (Table 1, entries 17 and 18). Water is used as an additive. Changing the water equivalence in the reaction prevented the desired product from being obtained (Table 1, entries 20 and 21). Additionally, increasing the water equivalence led to a decrease in product yield (Table 1, entry 19). With the optimized reaction conditions in hand, we next investigated the substrate scope utilizing different phenylhydrazine hydrochloride derivatives (8a–8s) and 3-aminocrotononitrile (11a) to synthesize various 5-cyano-4-methyl-2-phenyl-2*H*-1,2,3-triazole 1-oxide derivatives (12a–12s). In Scheme 2, the electron-withdrawing groups such as *p*-Br (8b), *p*-Cl (8c), and *p*-F (8d) in the para position provided the desired products in 78% (12b), 49% (12c), and 58% (12d) yields, respectively. The electron-donating group's *p*-CH_3_ (9e) and *p*-OCH_3_ (9f) yield 71% (12e) and 49% (12f), respectively. The electron-donating group by resonance effect like *p*-OCH_3_ resulted in a lower yield than *p*-CH_3_. However, strong electron-withdrawing groups like *p*-NO_2_ (8g) and *p*-CN (8h) yield 71% (12g) and 66% (12h), respectively. *Meta*-position having substitutions like *m*-CH_3_ (8k), *m*-Cl (8n), and *m*-Br (8m) provided good yields of 54% (12k), 54%(12j), and 68%(12i). Similarly, ortho substitution has yielded 78% to 45% yield. Hydrazine having aliphatic and heterocyclic substitutions is incompatible with the reaction condition. Next, we examined the reaction condition with phenylhydrazine hydrochloride (8a) and various aminocrotononitrile (11b–11e) derivatives.

**Scheme 3:**
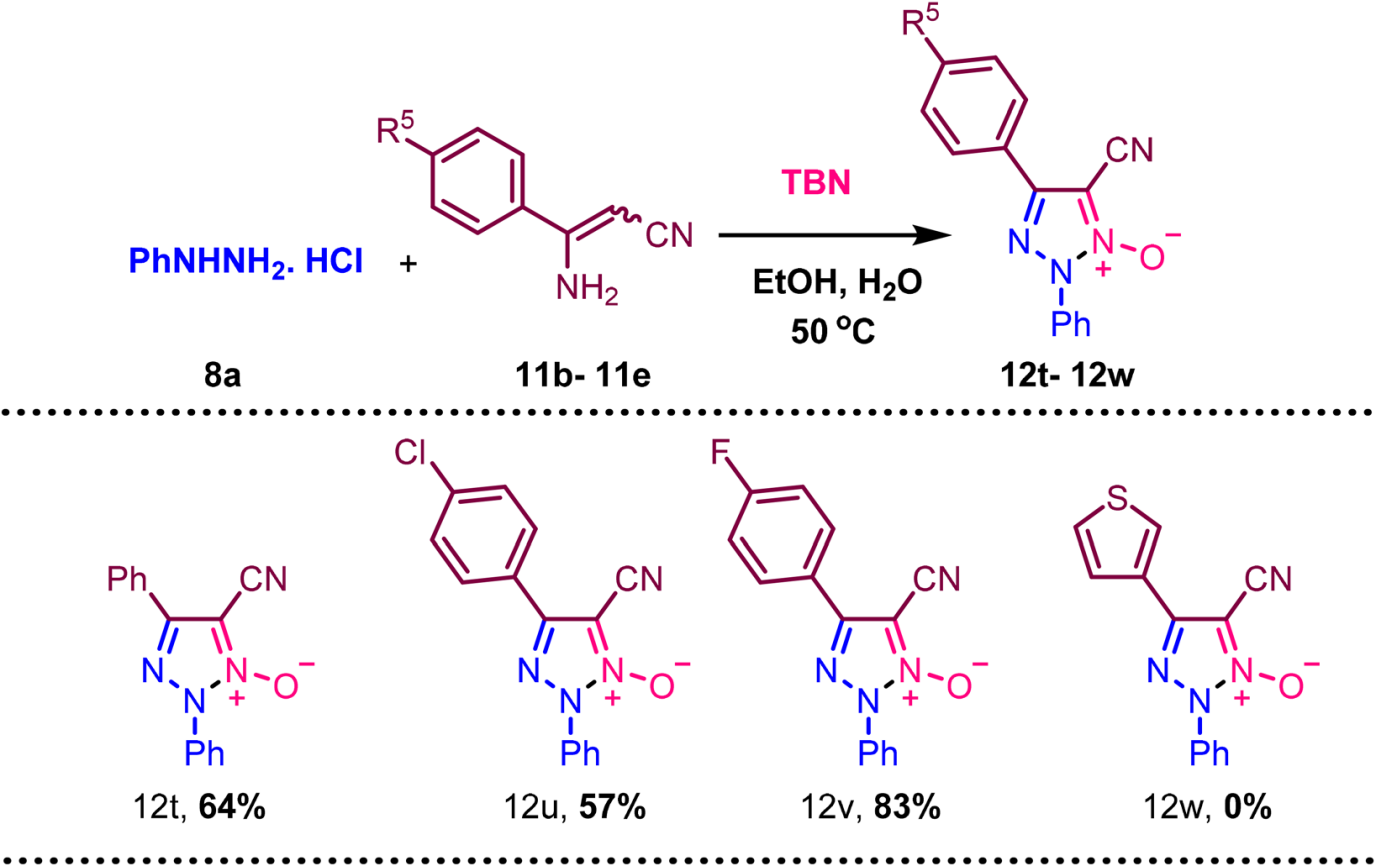
Synthesis of various 5-cyano-4-methyl-2-phenyl-2*H*-1,2,3-triazole 1-oxide (12t–12w) using various 3-aminocrotononitrile (11b–11f) and phenylhydrazine hydrochloride(8a). ^a^Reaction condition: 8a (1 mmol), 11 (1.5 mmol), ^*t*^BuONO (3 mmol), H_2_O (2 mmol), EtOH, 50 °C heating. Isolated yields are provided.

When aminocrotononitrile having substitutions like *p*-Cl (11c), and *p*-F (11d) in the phenyl ring resulted in good yields of 57% (12u), and 83% (12v), and heterocyclic compound (12w) is not compatible with the reaction condition (Scheme 3).

**Scheme 4:**
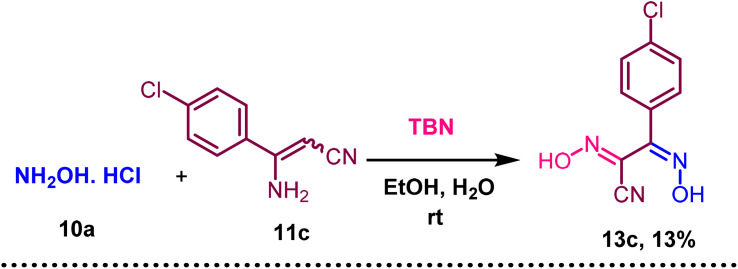
Synthesis of (1*Z*,2*Z*)-2-(4-chlorophenyl)-*N*-hydroxy-2-(hydroxyimino)acetimidoyl cyanide (13c) using aminocrotononitrile 11c and hydroxylamine hydrochloride (10a). ^a^ Reaction condition: 10a (1 mmol), 11 (1.2 mmol), ^*t*^BuONO (3 mmol), H_2_O (2 mmol), EtOH, RT. Isolated yields are provided.

Triazole-N-oxides 12t–12v were obtained in good yields. In Scheme 4, when hydroxylamine hydrochloride (10a) was used instead of phenylhydrazine hydrochloride (8a), compound 13c was formed and confirmed by ^1^H NMR, ^13^C NMR, and HRMS.

To predict the mechanism of the reaction, we conducted several control experiments.

**Scheme 5:**
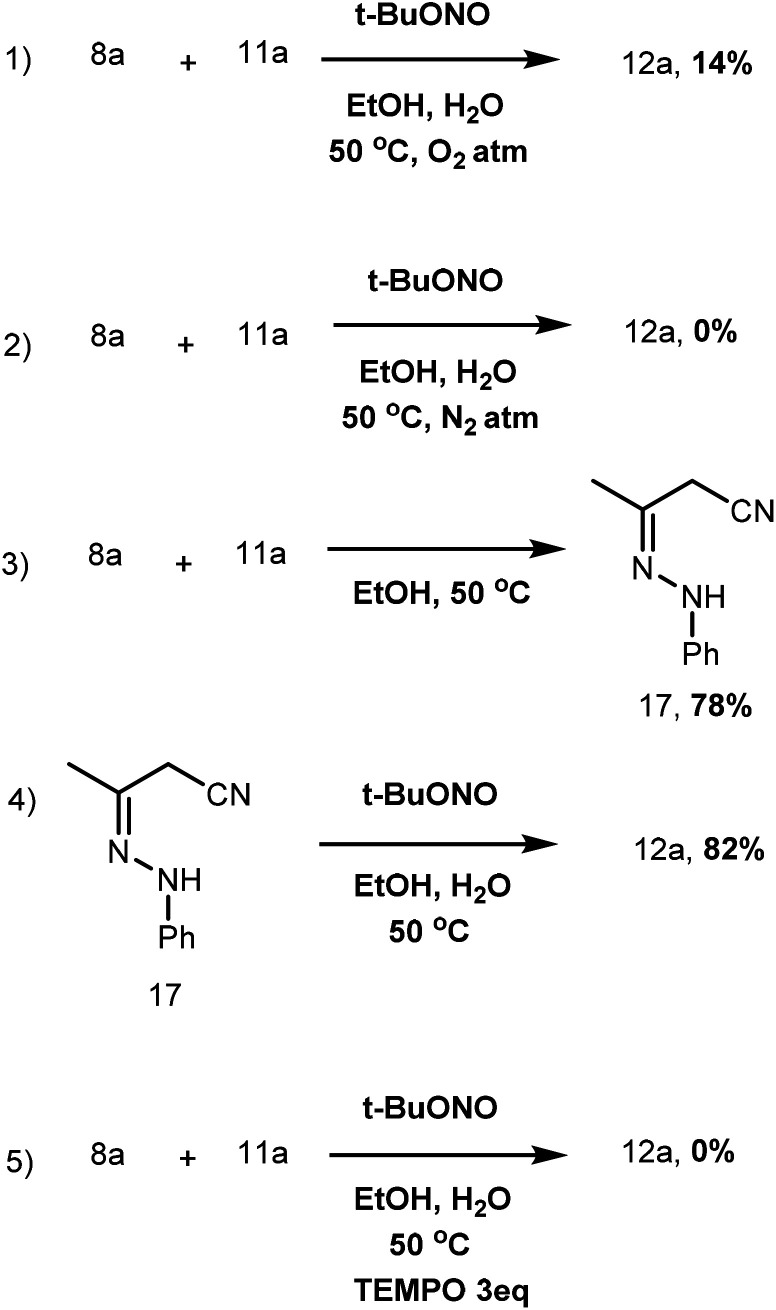
Control experiment.

When the reaction was carried out in O_2_ atm, it resulted in a low product yield. In the case of N_2_ atm, the product formation was completely inhibited (Scheme 5, eqn (1) and (2)). Thus, the closed condition favours product formation because, under aerobic conditions, NO radicals are transformed into NO_2_ radicals. An intermediate was identified when a reaction was carried out with 8a and 11a at 50 °C heating (Scheme 5, eqn (3)). The intermediate was isolated after the reaction was carried out under the optimized conditions, resulting in product formation with a yield of 82%, confirming that the product was formed exclusively *via* the intermediate (Scheme 5, eqn (4)). In addition, the product formation was completely inhibited when the reaction was carried with radical scavenger-TEMPO. Thus, the radical mechanism involved in the reaction is confirmed (Scheme 5, eqn (5))

**Scheme 6:**
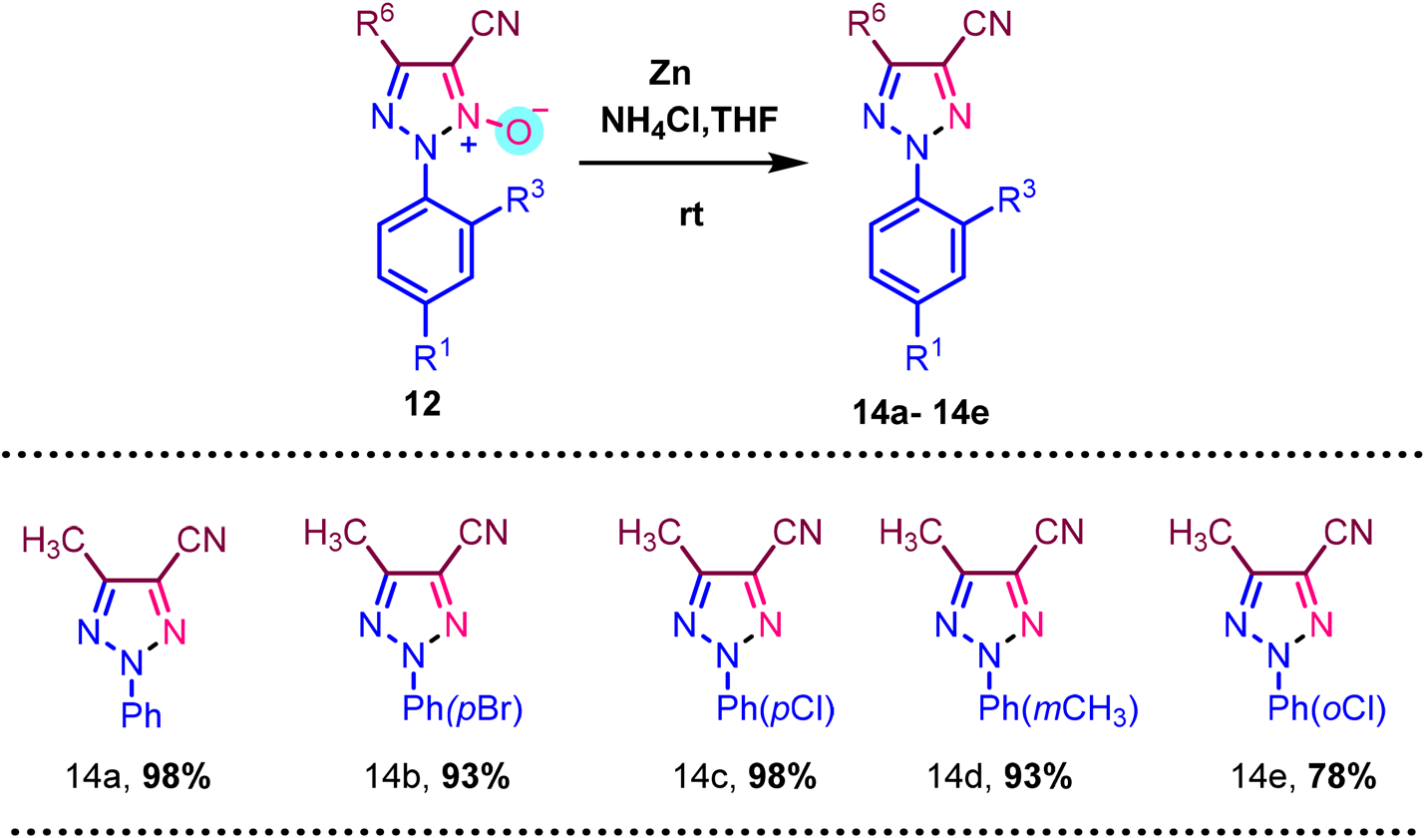
Deoxygenation of various N-oxides. ^a^ Reaction condition: 12 (1 mmol), Zn (6 mmol), 30% aqueous NH_4_Cl solution, THF, room temperature. Isolated yields are provided.

In Scheme 6, deoxygenation of N-oxide is demonstrated employing Zn and 30% NH_4_Cl solution and THF as a solvent. We used various N-oxide 14a–14e substrates for deoxygenation to form a triazole compound, resulting in a moderate to good yield of 98% to 78%.

**Scheme 7:**
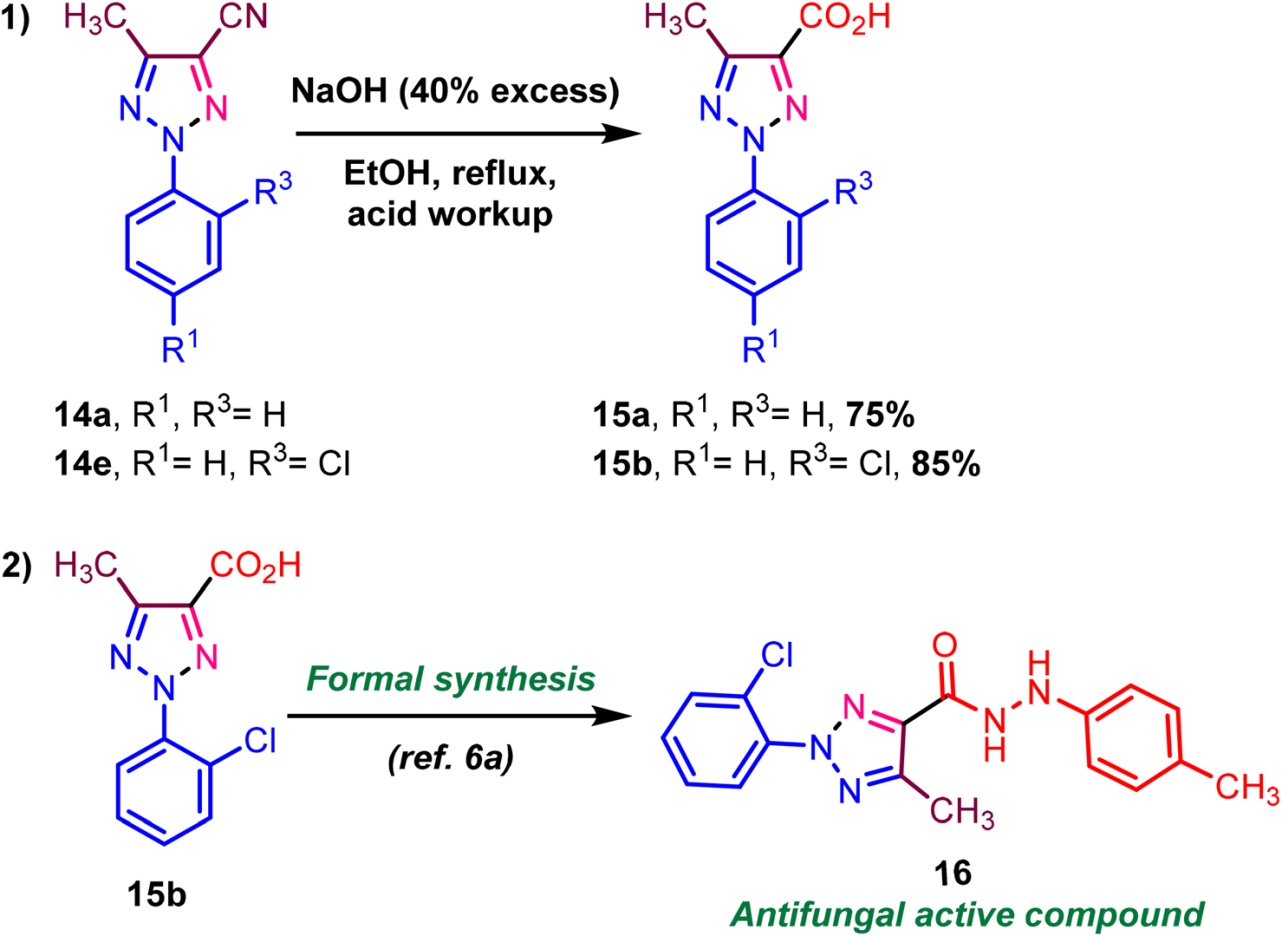
Synthetic transformations.

We demonstrated synthetic transformations using triazole-N-oxide functional group transformation. We executed the reaction to convert nitrile to acid *via* the nitrile hydrolysis reaction (Scheme 7, eqn (1)). In addition, the target compound 16 was formally synthesized in one step from acid derivatives (Scheme 7, eqn (1)).^6*a*^

**Scheme 8:**
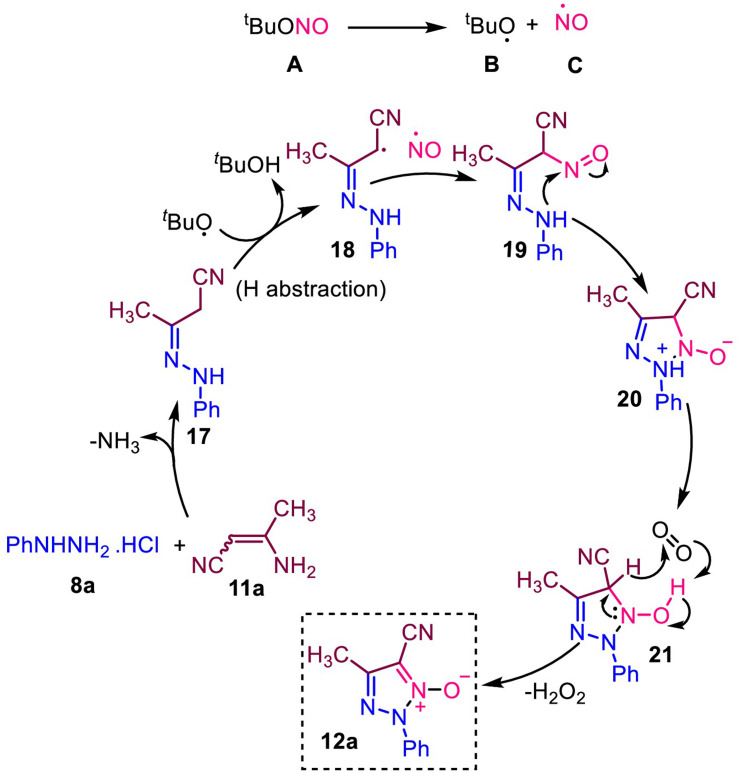
Possible reaction mechanism.

In Scheme 8, we explained possible reaction mechanisms based on the control experiments. The aza-Michael addition reaction of phenylhydrazine hydrochloride (8a) in 3-aminocrotononitrile (11a) forms hydrazone intermediate 17. The *tert*-butylnitrite (A) is degraded to form *tert*-butoxy (B) and NO (C) radicals. The *tert*-butoxy radical (B) removes hydrogen from the intermediate 17 to form a radical intermediate 18. The radical intermediate 18 combined with NO radical to form compound 20. Subsequent cyclization involving a nucleophilic nitrogen atom in NO led to the formation of triazole-N-oxide 12a.

In conclusion, we developed an efficient method for synthesizing 5-cyano-4-methyl-2-phenyl-2*H*-1,2,3-triazole 1-oxide under mild reaction conditions. *Tert*-butyl nitrite was successfully employed as a nitric oxide (NO) source, with phenylhydrazine hydrochloride and 3-aminocrotononitrile serving as readily available starting materials. The procedure utilizes environmentally friendly and easily accessible components, demonstrating a broad substrate scope and versatile synthetic transformations. Additionally, control experiments provided insight into a plausible reaction mechanism. This method enabled the synthesis of various triazole-N-oxide derivatives with moderate to good yields.

## Data availability

^1^H, ^13^C{^1^H} NMR and HRMS spectra of all known and unknown compounds, ORTEP table of compounds 12b, included in the ESI.†

## Conflicts of interest

There are no conflicts to declare.

## Supplementary Material

RA-015-D5RA01327E-s001

RA-015-D5RA01327E-s002

## References

[cit1] Gao H., Shreeve J. M. (2011). Chem. Rev..

[cit2] Zhou J., Zhang J., Wang B., Qiu L., Xu R., Sheremetev A. B. (2021). FirePhysChem.

[cit3] Vishnevskiy Y. V., Tikhonov D. S., Schwabedissen J., Stammler H., Moll R., Krumm B., Klapotke T. M., Mitzel N. W. (2017). Angew. Chem., Int. Ed..

[cit4] Lai W., Lian P., Ge Z., Liu Y., Yu T., Lv J. (2016). J. Mol. Modell..

[cit5] Dekorver K. A., Li H., Lohse A. G., Hayashi R., Lu Z., Zhang Y., Hsung R. P. (2010). Chem. Rev..

[cit6] Yin X., Liu X., Wu X., Liu X., Tian Q., Luo Q., Li Y. (2024). J. Agric. Food Chem..

[cit7] Mfuh A. M., Larionov O. V. (2015). Curr. Med. Chem..

[cit8] Ibrahim H., Furiga A., Najahi E., Pigasse Henocq C., Nallet J. P., Roques C., Aubouy A., Sauvain M., Constant P., Daffe M., Nepveu F. (2012). J. Antibiot..

[cit9] Buhl A. E., Waldon D. J., Baker C. A., Johnson G. A., Invest J. (1990). Dermatol.

[cit10] Bisby R. H. (1990). Biochem. Pharmacol..

[cit11] Vermeire K., Schols D., Bell T. W. (2004). Curr. Pharm. Des..

[cit12] Fang L., Zhang Y., Lehmann J., Wang Y., Ji H., Ding D. (2007). Bioorg. Med. Chem. Lett..

[cit13] Khaligh N. G. (2018). Mini-Rev. Org. Chem..

[cit14] Prat D., Hayler J., Wells J. A. (2014). Green Chem..

[cit15] Baruah B., Deb M. L. (2021). Org. Biomol. Chem..

[cit16] Winter J., Prenzel T., Wirtanen T., Schollmeyer D., Waldvogel S. R. (2022). Chem.–Eur. J..

[cit17] Titenkova K., Shuvaev A. D., Teslenko F. E., Zhilin E. S., Fershtat L. L. (2023). Green Chem..

[cit18] Mujahid M., Vara V., Arshad U., Gamidi R. K., Muthukrishnan M. (2024). J. Org. Chem..

[cit19] The CCDC deposition number for 12b = 2409286;† Unit Cell Parameters: a. 12.7279(5) b 11.0272(5) c 7.6786(4) P21/c

