# Supplementary material for: Catalyst-free synthesis of 1,2,3-triazole-N-oxide derivatives using tert-butyl nitrite: a novel strategy and synthetic applications
Source: RSC Adv. 2025 Apr 4;15(14):10574–81. doi: 10.1039/d5ra01327e (PMC11969441; doi:10.1039/d5ra01327e)

Supplementary Information (SI) for RSC Advances.
This journal is © The Royal Society of Chemistry 2025

Catalyst-Free Synthesis of 1,2,3-Triazole-N-Oxide Derivatives Using Tert-Butyl Nitrite: A Novel Strategy and Synthetic Applications

Karuppaiah Perumal[a], Markabandhu Shanthi[a], Vijayakumar Hemamalini[a], Bhaskaran Shankar[b], Subburethinam Ramesh[a]*

[a]Department of Chemistry, School of Chemical and Biotechnology, SASTRA Deemed University, Thanjavur 613 401, Tamil Nadu, India.

[b]Department of Chemistry, Thiagarajar College of Engineering, Madurai 625 015, Tamil Nadu, India.

Supporting information

S. No	Table of Content	Page No.
1.	General Information	2
2.	General procedure<br>2.1 General procedure for the synthesis of **12a**<br>2.2 General procedure for the synthesis of **13c**<br>2.3 General procedure for the synthesis of **14a**<br>2.4 General procedure for the synthesis of **15a**	2-4
3	Crystal data	5
4.	NMR and HRMS data	6-11
5.	References	11
6.	^{1}H & ^{13}C spectrum of the compounds along with HRMS data	12-53

1. General information:

All the reactions were carried out in oven-dried reaction vials. Thin-layer chromatography (TLC) was used to monitor reactions by Merck silica gel 60 F254 precoated plates. Silica mesh (60-120) from SRL Pvt. Ltd. And a hexane-ethyl acetate mixture were used for compound purification. ^{1}H and ^{13}C NMR spectra were recorded on a JEOL 600 MHz NMR instrument. $CDCl_3$ (δ 77.16 ppm, 7.26 ppm) and DMSO-d6 (δ 39.52 ppm, 2.5 ppm) solvent was used to take NMR data. Chemical shifts were reported in parts per million and multiplicities are as written as s (singlet), d (doublet), t (triplet), q (quartet), m (multiplet) and dd (doublet of doublet). Coupling constants (*J*) are reported in Hertz. Melting points were recorded on a Guna capillary melting point apparatus. High-resolution mass spectra (HRMS) were recorded on Waters - Xevo G2- XS - QToF. Single crystal X-ray diffraction data for the crystals of compound **12b** were measured at 296 K on a Rigaku Oxford XtaLAB Synergy diffractometer using a Mo Kα radiation [λ= 0.71073 Å]. The structures were solved by direct methods using SHELXS-97[1a] and refined using the SHELXL-2018/3 program[1b, 1c]. All non-hydrogen atoms were refined anisotropically. The solvents used were of laboratory grade and procured from pure chem (Dichloromethane, Hexane) and pure chem (Ethyl acetate). Various phenylhydrazine hydrochloride, 3-aminocrotononitrile, substituted benzonitriles, NaOH, and Tert-Butyl nitrite, *n*-Butyl nitrite, and *i*-Butyl nitrite were purchased from Alfa-aesar, Avra synthesis, Spectrochem, TCI and SRL.

2. General Procedure

2.1 General procedure for the synthesis of compound (12a)

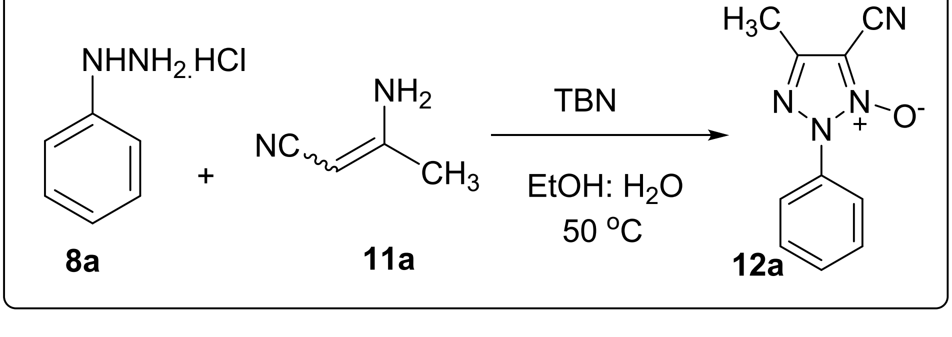

Phenylhydrazine hydrochloride (**8a**) (1 mmol) and 3-aminocrotonitrile (**11a**) (1.5 mmol) and EtOH (1.4 mL) were added to the reaction mixture and allowed to react for 30 minutes to form the intermediate, which was confirmed by thin layer chromatography (TLC). Tert-butyl nitrite (3 mmol) was then slowly added into the reaction mixture. The progress of the reaction was monitored by TLC, and upon completion, the reaction was quenched with water. The organic crude was extracted with ethyl acetate, washed with brine, and dried over anhydrous sodium sulfate. The crude product was subsequently purified by column chromatography (60-120 mesh) to yield the desired product (**12a**).

2.2 General procedure for the synthesis of compound 13c

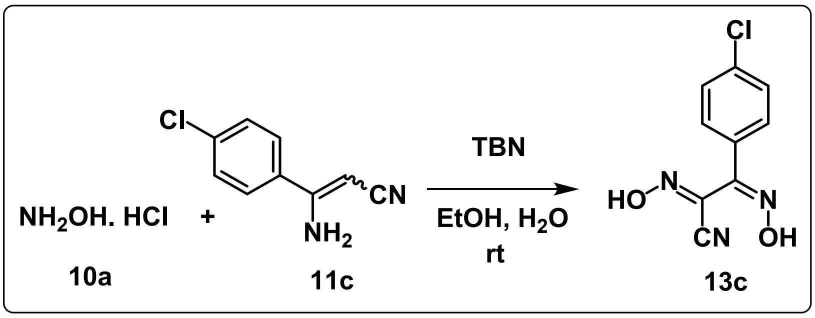

In a 5 mL glass vial, 1 mmol of hydroxylamine hydrochloride (**10a**) and crotononitrile **11c** were taken, followed by the addition of ethanol as a solvent. The reaction mixture was stirred for 30 minutes. After confirming the formation of the intermediate by TLC, TBN was added slowly to the reaction mass under ice-cold conditions. After, the completion of addition the reaction was changed into the room temperature. The reaction was monitored by TLC. Upon completion, the reaction was quenched with ice-cold water, and the reaction mass was extracted using ethyl acetate and water. The organic layer was separated, and the solvent was evaporated using a rotary evaporator. The resulting crude mass was subjected to column chromatography using silica gel (mesh size 60–120) as the stationary phase. A mobile phase of 10% ethyl acetate in hexanes was used for purification.

2.3 General procedure for the synthesis of compound 14a

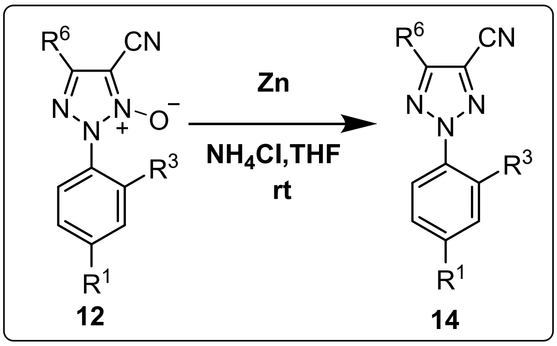

In an oven-dried glass vial, 1 mmol of **12a**, 6 mmol of zinc metal, and 30% NH_4Cl were added, with THF used as the solvent. The reaction was monitored by TLC. Upon completion, the reaction mass was filtered through Celite and extracted using ethyl acetate and water. The organic layer was separated, and the solvent was evaporated using a rotary evaporator. The resulting crude mass was purified by column chromatography using silica gel (mesh size 60–120) as the stationary phase, with 5% ethyl acetate in hexanes as the mobile phase. The same procedure was followed for the preparation of the corresponding derivatives.

2.4 General procedure for the synthesis of compound 15a

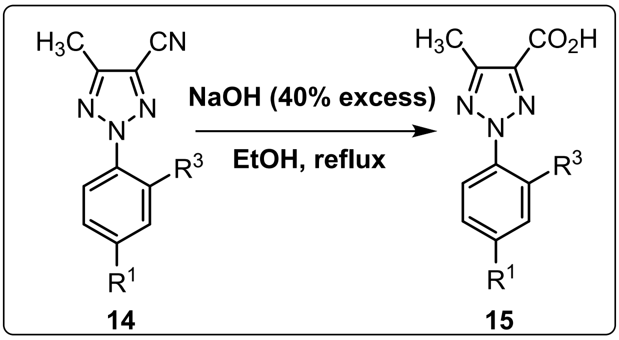

The compound **14a** was taken in a 25 mL round-bottom flask, to which 40% NaOH was added. Ethanol was used as the solvent, and the reaction was carried out under reflux at 80°C. The reaction progress was monitored by TLC. Upon completion, the reaction mass was quenched with 3M of HCl solution. The organic layer was then separated using ethyl acetate, and the crude product was recrystallized from ethanol.

3. Crystal data

Table 1. Crystal data and structure refinement for **12b**

Identification code	**12b**	
Empirical formula	C_{10} H_7 Br N_4 O	
Formula weight	279.11	
Temperature	296(2) K	
Wavelength	0.71073 Å	
Crystal system	Monoclinic	
Space group	P 21/c	
Unit cell dimensions	a = 12.7279(5) Å	α= 90°.
	b = 11.0272(5) Å	β= 95.347(4)°.
	c = 7.6786(4) Å	γ = 90°.
Volume	1073.03(9) Å^3	
Z	4	
Density (calculated)	1.728 Mg/m^3	
Absorption coefficient	3.814 mm^{-1}	
F(000)	552	
Crystal size	0.080 x 0.060 x 0.060 mm^3	
Theta range for data collection	3.215 to 24.999°.	
Index ranges	-15<=h<=14, -13<=k<=12, -9<=l<=9	
Reflections collected	10867	
Independent reflections	1876 [R(int) = 0.0466]	
Completeness to theta = 24.999°	99.9 %	
Absorption correction	Semi-empirical from equivalents	
Max. and min. transmission	1.00000 and 0.38688	
Refinement method	Full-matrix least-squares on F^2	
Data / restraints / parameters	1876 / 0 / 146	
Goodness-of-fit on F^2	1.057	
Final R indices [I>2sigma(I)]	R1 = 0.0359, wR2 = 0.0792	
R indices (all data)	R1 = 0.0539, wR2 = 0.0847	
Extinction coefficient	n/a	
Largest diff. peak and hole	0.300 and -0.457 e.Å^{-3}	

4. NMR data

5-Cyano-4-methyl-2-phenyl-2*H*-1,2,3-triazole 1-oxide (12a): Physical appearance = Light Yellow solid; Melting Point = 88-93 °C; Yield = 84%; Weight = 169.2 mg; R_f = 0.3 (10% Ethylacetate in hexane); ^{1}H NMR (600 MHz, $CDCl_3$) **δ** 7.83-7.81 (m, 2H), 7.49-7.47 (t, *J* = 6.0 Hz, 2H), 7.43 (t, *J* = 12 Hz, 1H), 2.42 (s, 3H); $^{13}C\{^1H\}$ NMR (150 MHz, $CDCl_3$) **δ** 143.77, 133.17, 129.07, 128.36, 121.71, 106.55, 105.39, 11.14; HRMS calculated for the molecular formula $C_{10}H_8N_4O$ = 201.0771([M+H]$^+$); found = 201.0773.

2-(4-Bromophenyl)-5-cyano-4-methyl-2*H*-1,2,3-triazole 1-oxide (12b): Physical appearance = Light yellow solid; Melting Point = 96-101 °C; Yield = 78%; Weight = 217.7 mg; R_f = 0.3 (10% Ethylacetate in hexane); ^{1}H NMR (600 MHz, $CDCl_3$) **δ** 7.83 (d, *J* = 8.94 Hz, 2H), 7.67 (d, *J* = 8.94 Hz, 2H), 2.48 (s, 3H); $^{13}C\{^1H\}$ NMR (150 MHz, $CDCl_3$) **δ** 145.04, 133.15, 132.54, 124.02, 123.75, 107.34, 106.55, 12.15; HRMS calculated for the molecular formula $C_{10}H_7BrN_4O$ = 278.9876 ([M+H]$^+$); found = 278.9803.

2-(4-Chlorophenyl)-5-cyano-4-methyl-2*H*-1,2,3-triazole 1-oxide (12c): Physical appearance = Light Yellow solid; Melting Point = 110-105 °C; Yield = 49%; Weight = 115.9 mg; R_f = 0.3 (10% Ethylacetate in hexane); ^{1}H NMR (600 MHz, $CDCl_3$) **δ** 7.82 (d, *J* = 9.0 Hz, 2H), 7.45 (d, *J* = 9.0 Hz, 2H), 2.41 (s, 3H); $^{13}C\{^1H\}$ NMR (150 MHz, $CDCl_3$) **δ** 144.03, 134.98, 131.61, 128.59, 122.66, 106.36, 105.54, 11.16; HRMS calculated for the molecular formula $C_{10}H_7ClN_4O$ = 235.0381 ([M+H]$^+$); found = 235.0308.

5-Cyano-2-(4-fluorophenyl)-4-methyl-2*H*-1,2,3-triazole 1-oxide (12d): Physical appearance = Yellow solid; Melting Point = 105-110 °C; Yield = 59%; Weight = 127.6 mg; R_f = 0.3 (10% Ethylacetate in hexane); ^{1}H NMR (600 MHz, $CDCl_3$) **δ** 7.86-7.79 (m, 2H), 7.18-7.15 (m, 2H), 2.41 (s, 3H); $^{13}C\{^1H\}$ NMR (150 MHz, $CDCl_3$) **δ** 162.74, 161.07, 143.89, 129.18, 129.16, 124.09, 124.03, 115.58, 115.42, 106.42, 105.34, 11.14; ^{19}F NMR ($CDCl_3$, 565 MHz, δ ppm) -108.59 to -108.64 (1F, m) HRMS calculated for the molecular formula $C_{10}H_7FN_4O$ = 219.0677 ([M+H]$^+$); found = 219.0613.

5-Cyano-4-methyl-2-(p-tolyl)-2H-1,2,3-triazole 1-oxide (12e): Physical appearance = Yellow solid; Melting Point = 98-103 °C; Yield = 72%; Weight = 153.2 mg; R_f = 0.3 (10% Ethylacetate in hexane); ^{1}H NMR (600 MHz, $CDCl_3$) **δ** 7.74 (d, *J* = 8.52 Hz, 2H), 7.34 (d, *J* = 8.16 Hz, 2H), 2.47 (s, 3H), 2.43 (s, 3H); $^{13}C\{^1H\}$NMR (150 MHz, $CDCl_3$) **δ** 144.56, 140.59, 131.68, 129.86, 122.69, 107.62, 106.16, 21.29, 12.10; HRMS calculated for the molecular formula $C_{11}H_{10}N_4O$ = 215.0927 ([M+H]$^+$); found = 215.0856.

5-Cyano-2-(4-methoxyphenyl)-4-methyl-2*H*-1,2,3-triazole 1-oxide (12f): Physical

appearance = Yellow solid; Melting Point = 110-115 °C; Yield = 49%; Weight = 113.7 mg; R_f= 0.3 (10% Ethylacetate in hexane); ^{1}H NMR (600 MHz, $CDCl_3$) **δ** 7.76 (dd, *J* = 2.16, 6.96 Hz, 2H), 7.02 (dd, *J* = 2.16, 6.96 Hz, 2H), 3.87 (s, 3H), 2.47 (s, 3H); $^{13}C\{^1H\}$ NMR (150 MHz, $CDCl_3$) **δ** 160.68, 144.45, 126.97, 124.78, 114.43, 107.66, 105.93, 55.64, 12.10; HRMS calculated for the molecular formula $C_{11}H_{10}N_4O_2$ = 231.0877 ([M+H]$^+$); found = 231.0804.

5-Cyano-4-methyl-2-(4-nitrophenyl)-2*H*-1,2,3-triazole 1-oxide (12g): Physical appearance = Yellow solid; Melting Point = 150-155 °C; Yield = 72%; Weight = 175.3 mg; R_f= 0.3 (10% Ethylacetate in hexane); ^{1}H NMR (600 MHz, $CDCl_3$) **δ** 8.42-8.40 (m, 2H), 8.30-8.28 (m, 2H), 2.52 (s, 3H); $^{13}C\{^1H\}$ NMR (150 MHz, $CDCl_3$) **δ** 147.32, 145.90, 138.51, 124.88, 122.05, 107.23, 106.98, 12.22; HRMS calculated for the molecular formula $C_{10}H_7N_5O_3$ = 246.0622 ([M+H]$^+$); found = 246.0626.

5-Cyano-2-(4-cyanophenyl)-4-methyl-2H-1,2,3-triazole 1-oxide (12h): Physical appearance = Light Yellow solid; Melting Point = 98-103 °C; Yield = 66%; Weight = 149.3 mg; R_f= 0.3 (10% Ethylacetate in hexane); ^{1}H NMR (600 MHz, $CDCl_3$) **δ** 8.20 (dd, *J* = 2.04, 6.96 Hz, 2H), 7.84 (dd, *J* = 1.98, 6.96 Hz, 2H), 2.51 (s, 3H); $^{13}C\{^1H\}$ NMR (150 MHz, $CDCl_3$) **δ** 145.71, 137.20, 133.32, 121.95, 117.40, 113.24, 107.10, 107.02, 12.19; HRMS calculated for the molecular formula $C_{11}H_7N_5O$ = 226.0723 ([M+H]$^+$); found = 226.0771.

2-(3-Bromophenyl)-5-cyano-4-methyl-2H-1,2,3-triazole 1-oxide (12i): Physical appearance = Light Yellow solid; Melting Point = 95-100 °C; Yield = 69%; Weight = 192.3 mg; R_f= 0.3 (10% Ethylacetate in hexane); ^{1}H NMR (600 MHz, $CDCl_3$) **δ** 8.04 (t, *J* = 1.92, 1.98 Hz, 1H), 7.86 (m, 1H), 7.56 (m, 1H), 7.35 (t, *J* = 8.16, 8.16 Hz, 1H), 2.42 (s, 3H); $^{13}C\{^1H\}$ NMR (150 MHz, $CDCl_3$) **δ** 145.30, 134.02, 133.42, 132.61, 130.14, 128.59, 107.42, 105.29, 12.33; HRMS calculated for the molecular formula $C_{10}H_7BrN_4O$ = 278.9876 ([M+H]$^+$); found = 278.9880.

2-(3-Chlorophenyl)-5-cyano-4-methyl-2*H*-1,2,3-triazole 1-oxide (12j): Physical appearance = Light Yellow solid; Melting Point = 94-99 °C; Yield = 55%; Weight = 128.1 mg; R_f= 0.3 (10% Ethylacetate in hexane); ^{1}H NMR (600 MHz, $CDCl_3$) **δ** 7.98-7.97 (m, 1H), 7.89-7.87 (m, 1H), 7.49-7.47 (m, 2H), 2.49 (s, 3H); $^{13}C\{^1H\}$ NMR (150 MHz, $CDCl_3$) **δ** 144.14, 134.17, 133.92, 129.38, 129.04, 121.44, 119.28, 106.31, 105.65, 11.16; HRMS calculated for the molecular formula $C_{10}H_7ClN_4O$ = 235.0381 ([M+H]$^+$); found = 235.0377.

5-Cyano-4-methyl-2-(m-tolyl)-2*H*-1,2,3-triazole 1-oxide (12k): Physical appearance = Yellow solid; Melting Point = 92-97 °C; Yield = 55%; Weight = 117.0 mg; R_f= 0.3 (10%

Ethylacetate in hexane); ^{1}H NMR (600 MHz, $CDCl_3$) **δ** 7.61 (m, 2H), 7.35 (m, 1H), 7.24 (d, *J* = 7.62 Hz, 1H), 2.41 (s, 3H), 2.37 (s, 3H); $^{13}C\{^{1}H\}$ NMR (150 MHz, $CDCl_3$) **δ** 144.64, 139.65, 134.02, 130.89, 129.12, 123.22, 119.90, 107.58, 106.30, 21.37, 12.12; HRMS calculated for the molecular formula $C_{11}H_{10}N_4O$ = 215.0927 ($[M+H]^+$); found = 215.0929.

5-Cyano-2-(2-fluorophenyl)-4-methyl-2*H*-1,2,3-triazole 1-oxide (12l): Physical appearance = Pale yellow solid; Melting Point = 160-165 °C; Yield = 78%; Weight = 170.2 mg; R_f = 0.3 (10% Ethylacetate in hexane); ^{1}H NMR (600 MHz, DMSO-d6) **δ** 7.36 (t, *J* = 5.2 Hz, 1H), 7.08-7.01 (m, 2H), 6.80-6.77 (m, 1H), 2.04 (s, 3H); $^{13}C\{^{1}H\}$ NMR (150 MHz, DMSO-d6) **δ** 157.89, 156.20, 146.45, 134.98, 134.92, 130.13, 126.11, 126.09, 120.86, 120.77, 117.62, 117.50, 108.68, 105.74, 12.38; ^{19}F NMR (DMSO-d6, 565 MHz, δ ppm) -118.31 to -118.84 (1F, m); HRMS calculated for the molecular formula $C_{10}H_7FN_4O$ = 218.0604 ([M+]); found = 218.0634.

2-(2-Bromophenyl)-5-cyano-4-methyl-2*H*-1,2,3-triazole 1-oxide (12m): Physical appearance = Yellow solid; Melting Point = 105-110 °C; Yield = 46%; Weight = 127.0 mg; R_f = 0.3 (10% Ethylacetate in hexane); ^{1}H NMR (600 MHz, $CDCl_3$) **δ** 8.04 (t, *J* = 1.92, 1.98 Hz, 1H), 7.86 (m, 1H), 7.56 (m, 1H), 7.35 (t, J = 8.16 Hz, 1H), 2.42 (s, 3H); $^{13}C\{^{1}H\}$ NMR: (150 MHz, $CDCl_3$) **δ** 145.30, 134.02, 133.42, 132.61, 130.14, 128.59, 122.47, 107.42, 105.29, 12.33; HRMS calculated for the molecular formula $C_{10}H_7BrN_4O$ = 278.9876 ($[M+H]^+$); found = 278.9883.

2-(2-Chlorophenyl)-5-cyano-4-methyl-2*H*-1,2,3-triazole 1-oxide (12n): Physical appearance = Brown solid; Melting Point = 105-110 °C; Yield = 72%; Weight = 167.8 mg; R_f = 0.3 (10% Ethylacetate in hexane); ^{1}H NMR (600 MHz, DMSO-d6) **δ** 7.70 -7.64 (m, 2H), 7.61 (m, 1H), 7.50 (t, *J* = 7.74 Hz,1H), 2.31 (s, 3H); $^{13}C\{^{1}H\}$ NMR (150 MHz, DMSO-d6) **δ** 145.71, 134.11, 131.90, 130.86, 130.58, 130.44, 128.76, 108.22, 104.96, 11.96; HRMS calculated for the molecular formula $C_{10}H_7ClN_4O$ = 235.0381 ($[M+H]^+$); found = 235.0364.

5-Cyano-4-methyl-2-(o-tolyl)-2*H*-1,2,3-triazole 1-oxide (12o): Physical appearance = Brown solid; Melting Point = 99-104 °C; Yield = 57%; Weight = 169.7 mg; R_f = 0.3 (10% Ethylacetate in hexane); ^{1}H NMR (600 MHz, $CDCl_3$) **δ** 7.92-7.87 (m, 4H), 7.54 -7.48 (m, 3H), 7.46-7.44 (m, 2H); $^{13}C\{^{1}H\}$ NMR (150 MHz, $CDCl_3$) **δ** 161.74, 160.07, 143.89, 129.18, 129.16, 124.09, 124.03, 115.58, 115.42, 106.42, 105.34, 11.14; HRMS calculated for the molecular formula $C_{11}H_{10}N_4O$ = 215.0927 ($[M+H]^+$); found = 215.0935.

5-Cyano-2-(3,4-dimethylphenyl)-4-methyl-2*H*-1,2,3-triazole 1-oxide (12p): Physical appearance = Orange solid; Melting Point = 94-99 °C; Yield = 57%; Weight = 130.6 mg; R_f

= 0.3 (10% Ethylacetate in hexane); ^{1}H NMR (600 MHz, $CDCl_3$) **δ** 7.53 (s, 1H), 7.50 (m, 1H), 7.20 (d, *J* = 8.16 Hz, 1H), 2.40 (s, 3H), 2.26 (s, 6H); $^{13}C\{^1H\}$ NMR (150 MHz, $CDCl_3$) **δ** 143.49, 138.39, 137.09, 130.82, 129.29, 122.76, 119.33, 106.67, 105.13, 18.82, 18.66, 11.17; HRMS calculated for the molecular formula $C_{12}H_{12}N_4O$ = 229.1084 ($[M+H]^+$); found = 229.1105.

5-Cyano-2,4-diphenyl-2*H*-1,2,3-triazole 1-oxide (12t): Physical appearance = Brown solid; Melting Point = 154-159 °C; Yield = 64%; Weight = 167.1 mg; R_f = 0.3 (10% Ethylacetate in hexane); ^{1}H NMR (600 MHz, $CDCl_3$) **δ** 7.94 (m, 3H), 7.92 (s, 1H), 7.51 (t, J = 7.2, 7.98 Hz, 2H), 7.47 (m, 4H); $^{13}C\{^1H\}$ NMR (150 MHz, $CDCl_3$) **δ** 145.35, 134.23, 131.25, 130.33, 129.46, 129.36, 126.88, 126.32, 122.87, 108.67, 104.16; HRMS calculated for the molecular formula $C_{15}H_{10}N_4O$ = 263.0927 ($[M+H]^+$); found = 263.0930.

4-(4-Chlorophenyl)-5-cyano-2-phenyl-2*H*-1,2,3-triazole 1-oxide (12u): Physical appearance = Brown colour solid; Melting Point = 120-125 °C; Yield = 57%; Weight = 169.7 mg; R_f = 0.3 (10% Ethylacetate in hexane); ^{1}H NMR (600 MHz, $CDCl_3$) **δ** 8.03 (m, 2H), 7.99 (d, *J* = 1.40 Hz, 1H), 7.98 (m, 1H), 7.59 (m, 2H), 7.56 (m, 1H), 7.26 (d, *J* = 1.44 Hz, 1H), 7.24 (m, 1H); $^{13}C\{^1H\}$ NMR (150 MHz, $CDCl_3$) **δ** 160.18, 159.50, 144.42, 134.10, 130.37, 129.44, 128.47, 128.42, 122.85, 116.70, 116.55, 108.54, 103.92; HRMS calculated for the molecular formula $C_{15}H_9ClN_4O$ = 297.0538 ([M+H]+); found = 297.0539.

5-Cyano-4-(4-fluorophenyl)-2-phenyl-2*H*-1,2,3-triazole 1-oxide (12v): Physical appearance = Yellow solid; Melting Point = 170-175 °C; Yield = 83%; Weight = 233.2 mg; R_f = 0.3 (10% Ethylacetate in hexane); ^{1}H NMR (600 MHz, $CDCl_3$) **δ** 8.03 (m, 2H), 7.99 (d, *J* = 1.40 Hz, 1H), 7.98 (m, 1H), 7.59 (m, 2H), 7.56 (m, 1H), 7.26 (d, *J* = 1.44 Hz, 1H), 7.24 (m, 1H); $^{13}C\{^1H\}$ NMR (150 MHz, $CDCl_3$) **δ** 165.18, 163.50, 144.42, 134.10, 130.37, 129.44, 128.47, 128.42, 122.85, 116.70, 116.55, 108.54, 103.92; HRMS calculated for the molecular formula $C_{15}H_9FN_4O$ = 281.0833 ($[M+H]^+$); found = 281.0828.

(1Z,2Z)-2-(4-Chlorophenyl)-N-hydroxy-2-(hydroxyimino)acetimidoyl cyanide (13c): Physical appearance = White solid; Melting Point = 98-103 °C; Yield = 13%; Weight = 29.1 mg; R_f = 0.3 (10% Ethylacetate in hexane); ^{1}H NMR (600 MHz, DMSO-d6) **δ** 13.82 (s, 1H), 12.53 (s, 1H), 7.50 (d, *J* = 8.5 Hz, 2H), 7.40 (d, *J* = 8.5 Hz, 2H), 2.55 – 2.45 (m, 4H); $^{13}C\{^1H\}$ NMR (150 MHz, DMSO-d6) **δ** 150.73, 134.33, 132.10, 131.61, 128.57, 128.49, 109.48; HRMS calculated for the molecular formula $C_9H_6ClN_3O_2$ = 224.0221 ($[M+H]^+$); found = 224.0225.

5-Methyl-2-phenyl-2*H*-1,2,3-triazole-4-carbonitrile (14a): Physical appearance = White

solid; Melting Point = 97-102 °C; Yield = 99%; Weight = 182.0 mg; R_f = 0.3 (10% Ethylacetate in hexane); ^1H NMR (600 MHz, $CDCl_3$) **δ** 7.95 (d, J = 8.04 Hz, 2H), 7.44- 7.41 (m, J = 7.68 Hz, 2H), 7.34 (t, J = 4.9 Hz, 1H), 2.45 (s, 3H); $^{13}C\{^1H\}$ NMR (150 MHz, $CDCl_3$) **δ** 149.35, 137.88, 128.49, 127.92, 120.97, 118.26, 110.58, 9.31; HRMS calculated for the molecular formula $C_{10}H_8N_4$ = 185.0822 ($[M+H]^+$); found = 185.0815.

2-(4-Bromophenyl)-5-methyl-2*H*-1,2,3-triazole-4-carbonitrile (14b): Physical appearance = White solid; Melting Point = 95-100 °C; Yield = 94%; Weight = 246.3 mg; R_f = 0.3 (10% Ethylacetate in hexane); ^1H NMR (600 MHz, $CDCl_3$) **δ** 7.86 – 7.79 (m, 2H), 7.17 (dd, J = 7.98, 9.12 Hz, 2H), 2.41 (s, 3H); $^{13}C\{^1H\}$ NMR (150 MHz, $CDCl_3$) **δ** 144.03, 134.98, 131.61, 128.59, 122.66, 106.36, 105.54, 11.16; HRMS calculated for the molecular formula $C_{10}H_7BrN_4$ = 262.9927 ($[M+H]^+$); found = 262.9936.

2-(4-Chlorophenyl)-5-methyl-2*H*-1,2,3-triazole-4-carbonitrile (14c): Physical appearance = White solid; Melting Point = 88-93 °C; Yield = 99%; Weight = 216.0 mg; R_f = 0.3 (10% Ethylacetate in hexane); ^1H NMR (600 MHz, $CDCl_3$) **δ** 7.79 – 7.72 (m, 2H), 7.17 (dd, J = 7.98, 9.12 Hz, 2H), 2.41 (s, 3H); $^{13}C\{^1H\}$ NMR (150 MHz, $CDCl_3$) **δ** 145.04, 133.11, 132.54, 124.9, 123.75, 107. 35, 106.56, 12.15; HRMS calculated for the molecular formula $C_{10}H_7ClN_4$ = 219.0432 ($[M+H]^+$); found = 219.0384.

5-Methyl-2-(m-tolyl)-2*H*-1,2,3-triazole-4-carbonitrile (14d): Physical appearance =White solid; Melting Point = 85-90 °C; Yield = 94%; Weight = 185.5 mg; R_f = 0.3 (10% Ethylacetate in hexane); ^1H NMR (600 MHz, $CDCl_3$) **δ** 7.74 (d, J = 8.52 Hz, 2H), 7.34 (d, J = 8.3 Hz, 2H), 2.47 (s, 3H), 2.43 (s, 3H); $^{13}C\{^1H\}$ NMR (150 MHz, $CDCl_3$) **δ** 144.56, 140.59, 131.68, 129.86, 122.69, 107.62, 106.16, 21.29, 12.10; HRMS calculated for the molecular formula $C_{11}H_{10}N_4$ = 199.0978 ($[M+H]^+$); found = 199.0984.

2-(2-Chlorophenyl)-5-methyl-2*H*-1,2,3-triazole-4-carbonitrile (14e): Physical appearance =Brown liquid; Yield = 78%; Weight = 170.5 mg; R_f = 0.3 (10% Ethylacetate in hexane); ^1H NMR (600 MHz, $CDCl_3$) **δ** 7.51 (m, 2H), 7.41 (m, 1H), 7.36 (m, 1H), 2.49 (s, 3H); $^{13}C\{^1H\}$ NMR (150 MHz, $CDCl_3$) **δ** 144.14, 134.17, 133.92, 129.03, 121.44, 119.28, 106.31, 105.65, 11.16; HRMS calculated for the molecular formula $C_{10}H_7ClN_4$ = 219.0432 ($[M+H]^+$); found = 219.0436.

5-Methyl-2-phenyl-2*H*-1,2,3-triazole-4-carboxylic acid (16a): Physical appearance =Brown solid; Melting Point = 110-115 °C; Yield = 86%; Weight = 174.3 mg; R_f = 0.9 (50% Ethylacetate in hexane); ^1H NMR (600 MHz, DMSO-d6) **δ** 13.55 (s, 1H), 8.07-8.04 (m, 2H), 7.65-7.63 (m, 2H), 7.53-7.50 (m, 1H), 2.57 (s, 3H); $^{13}C\{^1H\}$ NMR (150 MHz, DMSO-d6) **δ**

162.57, 148.80, 139.16, 139.11, 130.25, 128.87, 119.16, 11.79. The spectral data exactly matches the literature data. [2]

2-(2-Chlorophenyl)-5-methyl-2*H*-1,2,3-triazole-4-carboxylic acid (16b): Physical appearance = Light yellow solid; Melting Point = 125-130 °C; Yield = 75%; Weight = 179.2 mg; R_f = 0.9 (50% Ethylacetate in hexane); ^{1}H NMR: (600 MHz, DMSO-d6) **δ** 13.73 (s, 1H), 7.76-7.70 (m, 2H), 7.63-7.61 (m, 1H), 7.57 (t, *J* = 7.68 Hz, 1H), 2.51 (s, 3H); $^{13}C\{^1H\}$ NMR (150 MHz, DMSO-d6) **δ** 162.58, 148.81, 139.16, 139.11, 130.25, 128.87, 119.16, 40.59, 11.79. The spectral data exactly matches the literature data. [3]

5. References

1. a) Sheldrick, G. M. *Program for Crystal Structure Solution. SHELXS-97*; University of Göttingen: Göttingen, Germany, 1997. b) Sheldrick, G. M. A short history of SHELX. *Acta Crystallogr., Sect. A: Found. Crystallogr.* 2008, **64**, 112– 122. c) Sheldrick, G. M. Crystal structure refinement with SHELXL. *Acta Crystallogr., Sect. C: Struct. Chem.* 2015, **71**, 3–8.
2. M. Mujahid, V. Vara, U. Arshad, R. K. Gamidi, M. Muthukrishnan, *J. Org. Chem*. 2024, 89, **23**, 16990–16998.
3. S. K. Singh, L. A. Summers, *J. Heterocycl. Chem*, 1987, **24** (4), 933–939.

^{1}H NMR of Compound 12a

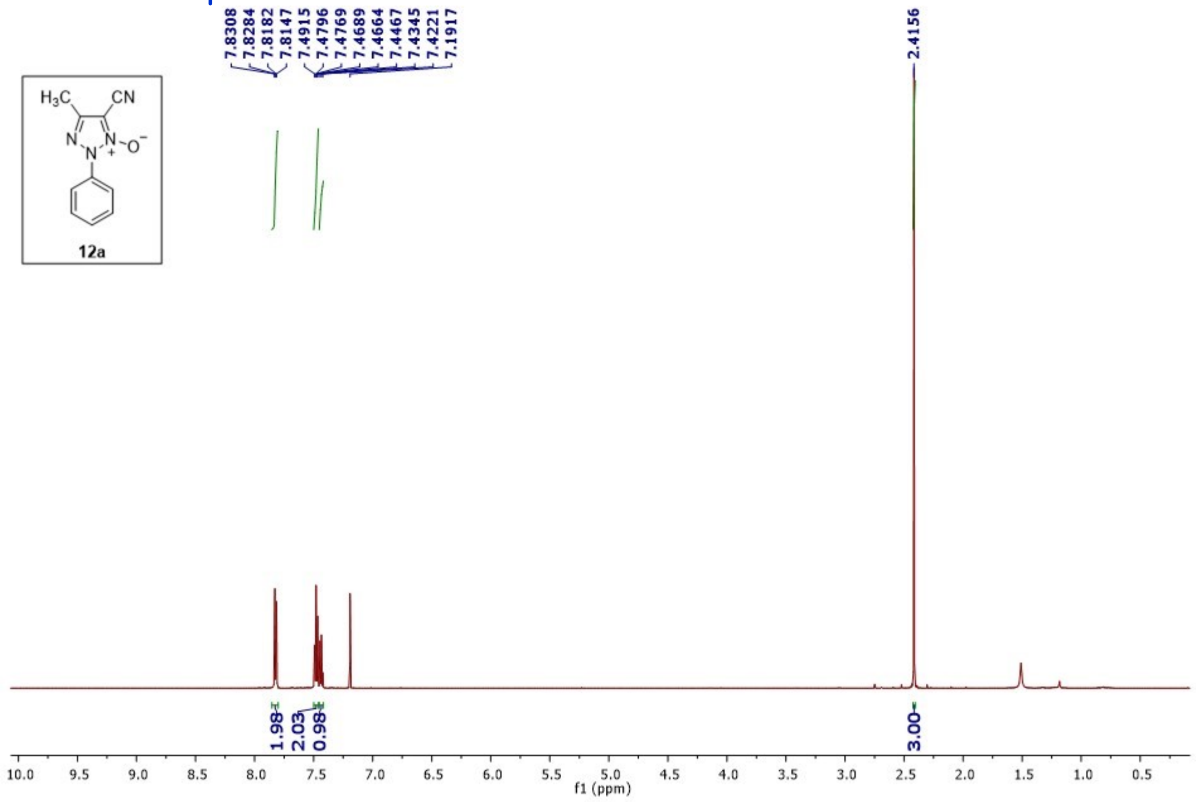

^{13}C NMR of Compound 12a

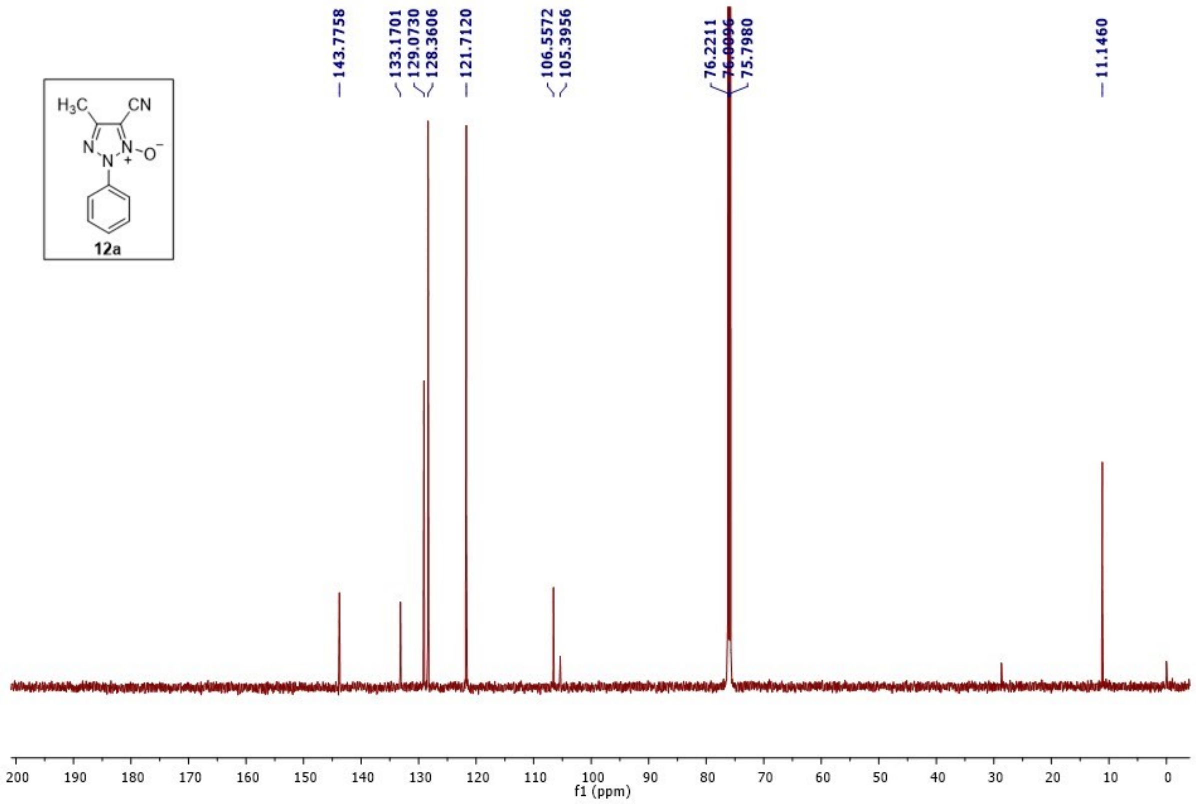

HRMS of Compound 12a

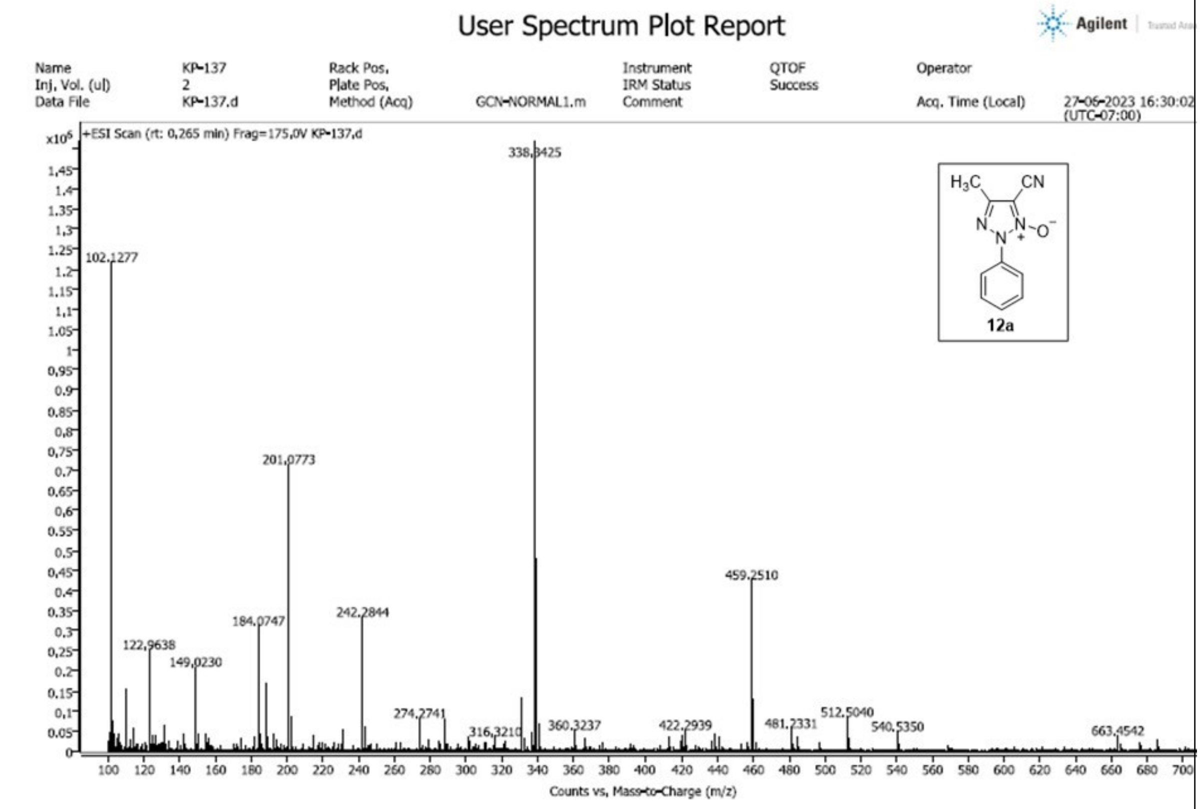

^{1}H NMR of Compound 12b

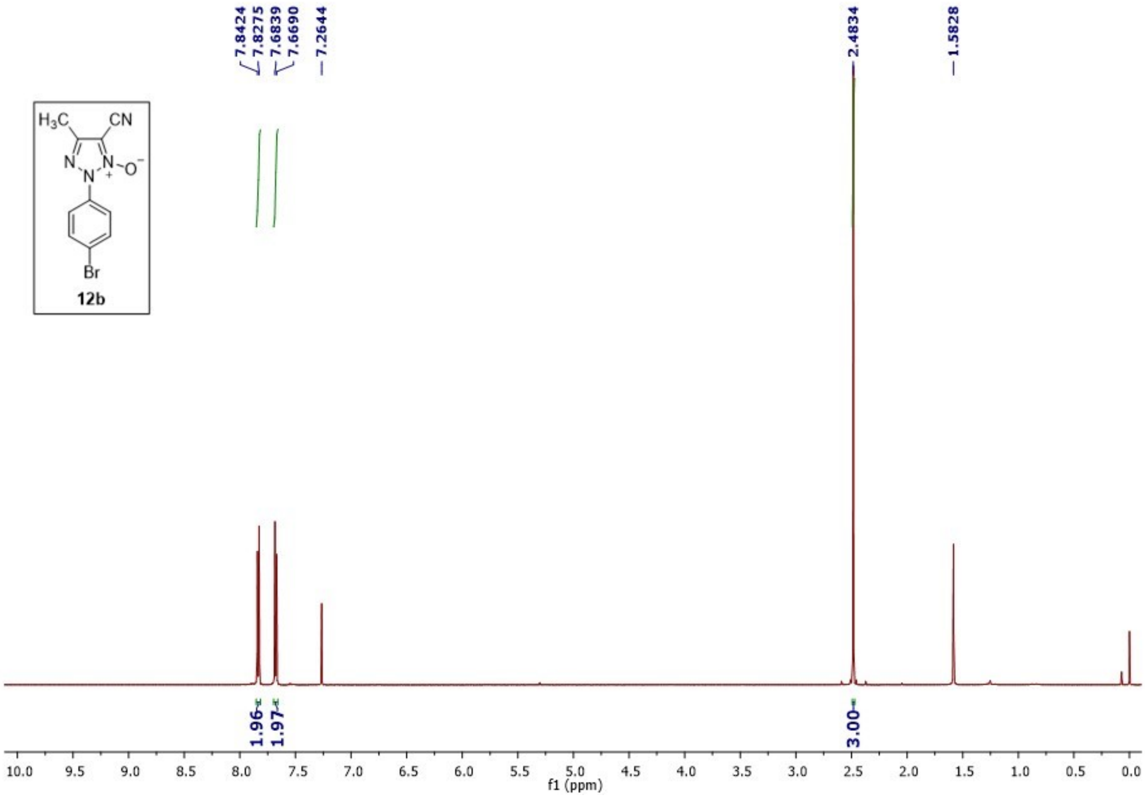

C NMR of Compound 12b

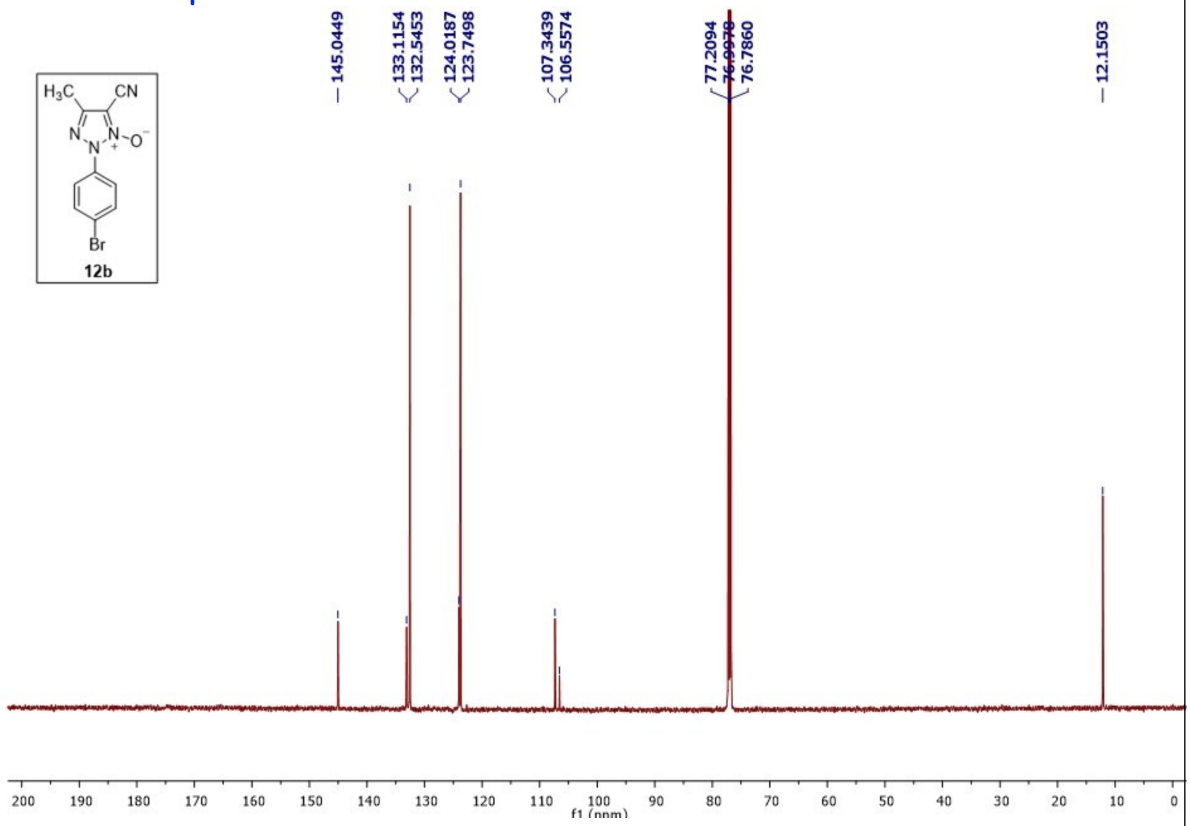

HRMS of Compound 12b

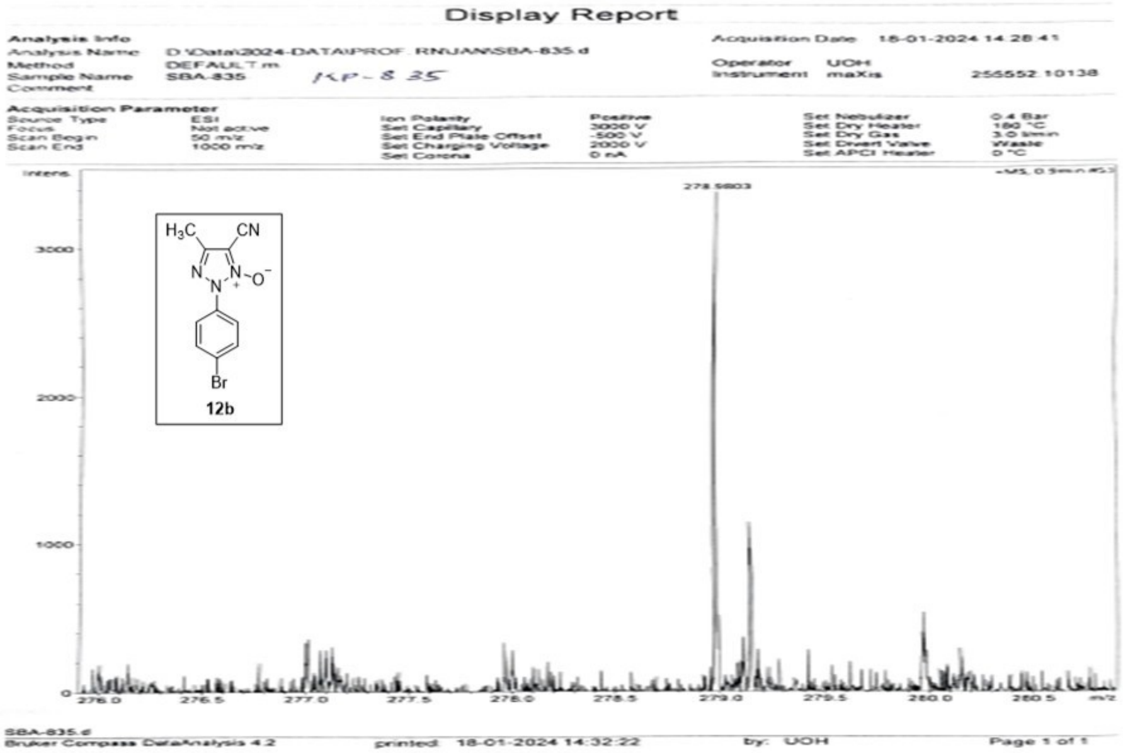

¹H NMR of Compound 12c

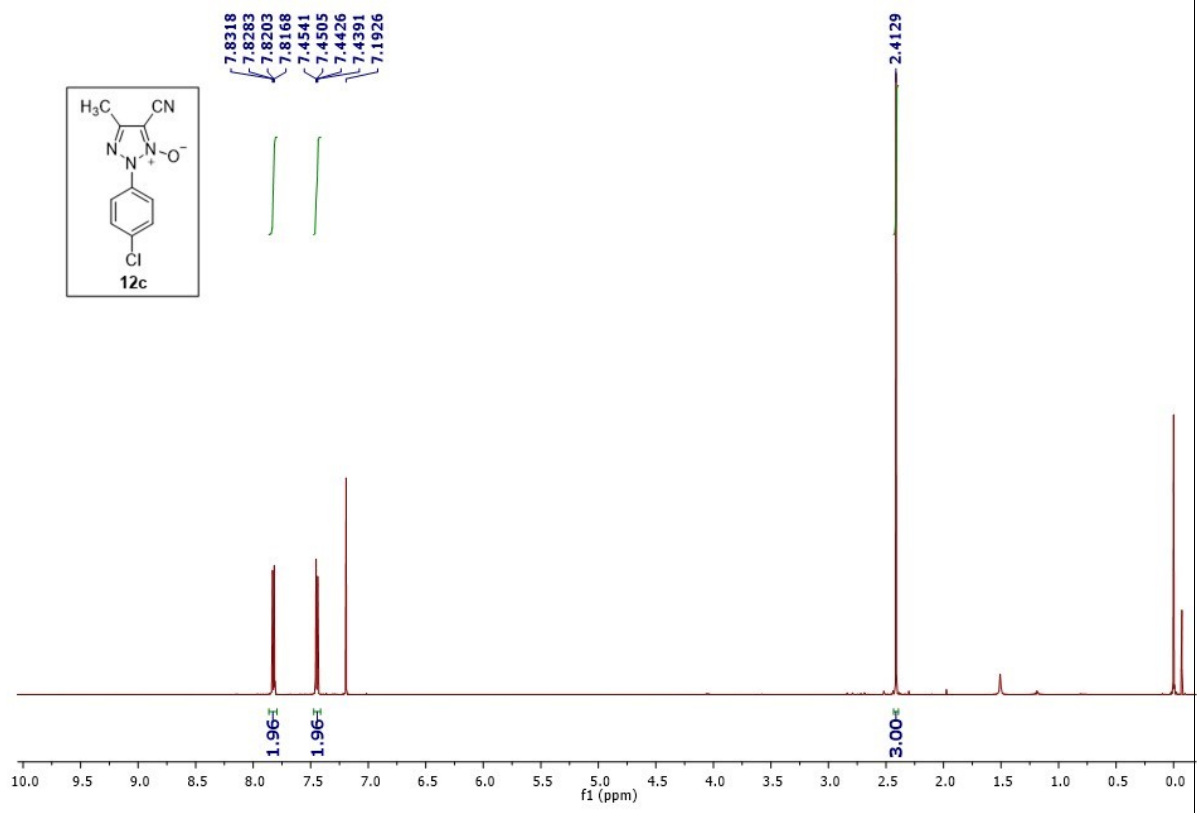

¹³C NMR of Compound 12c

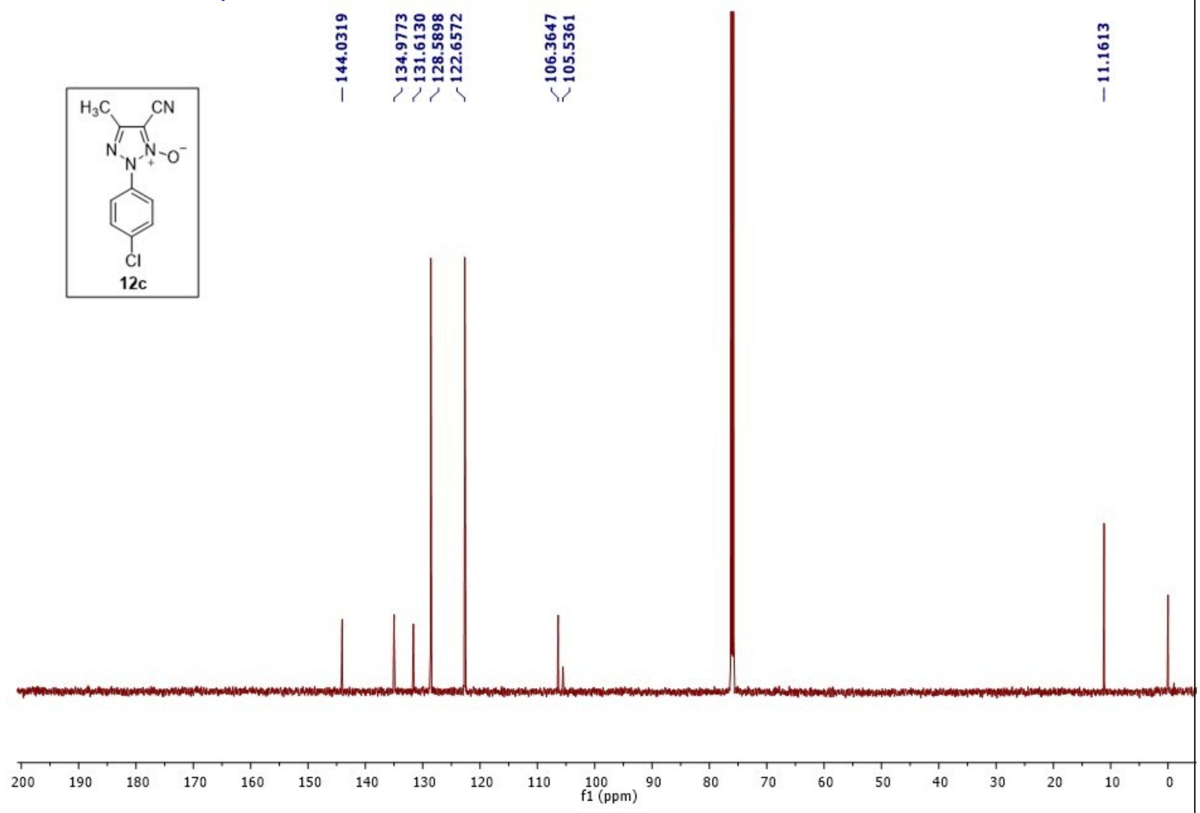

HRMS NMR of Compound 12c

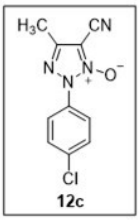

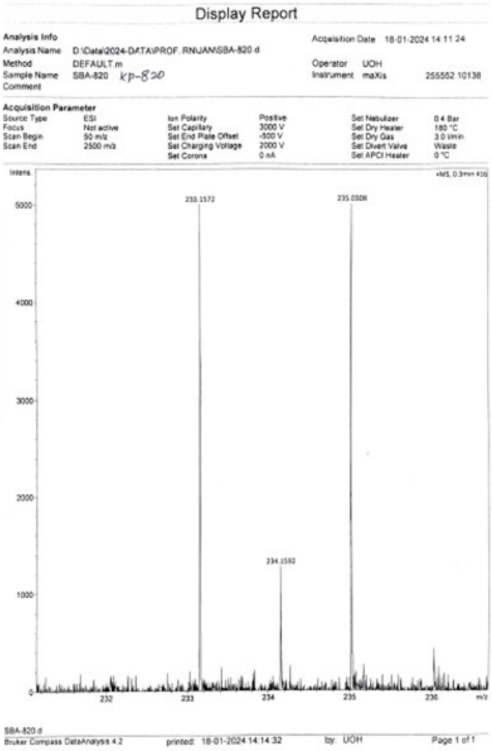

^{1}H NMR of Compound 12d

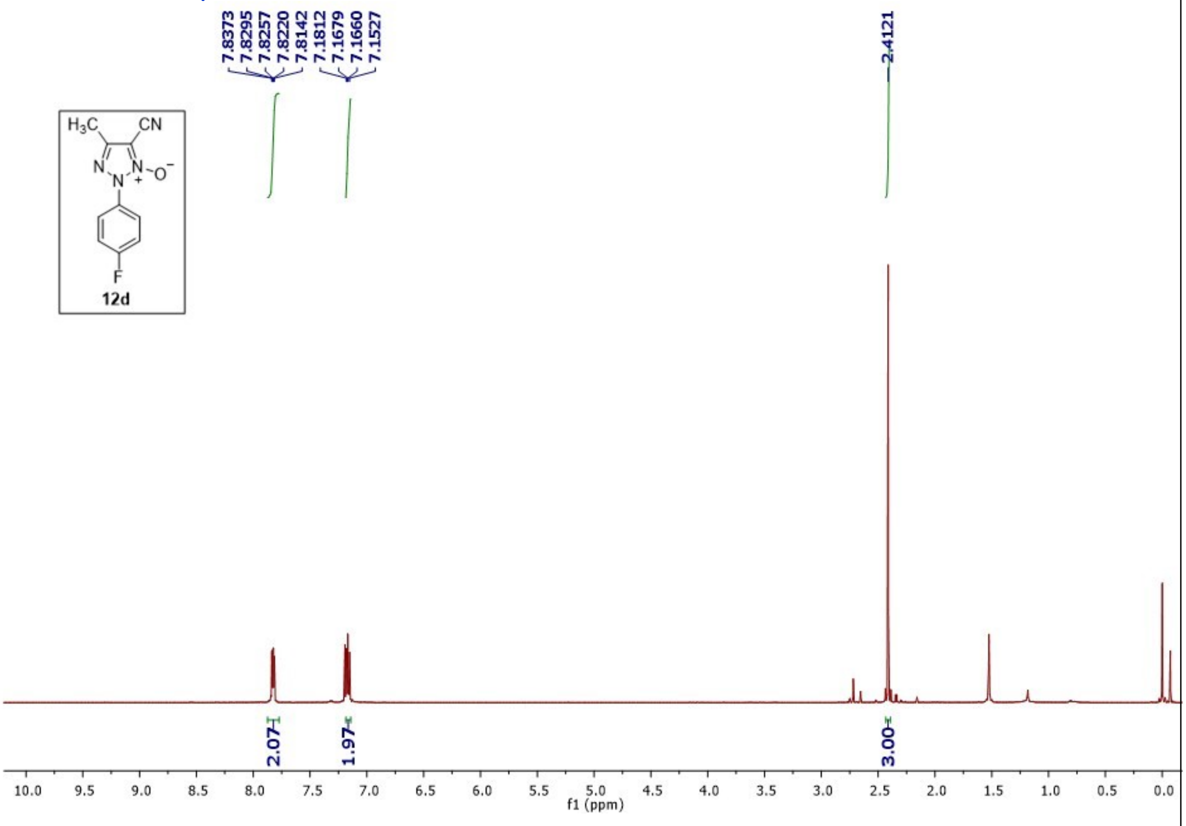

^{13}C NMR of Compound 12d

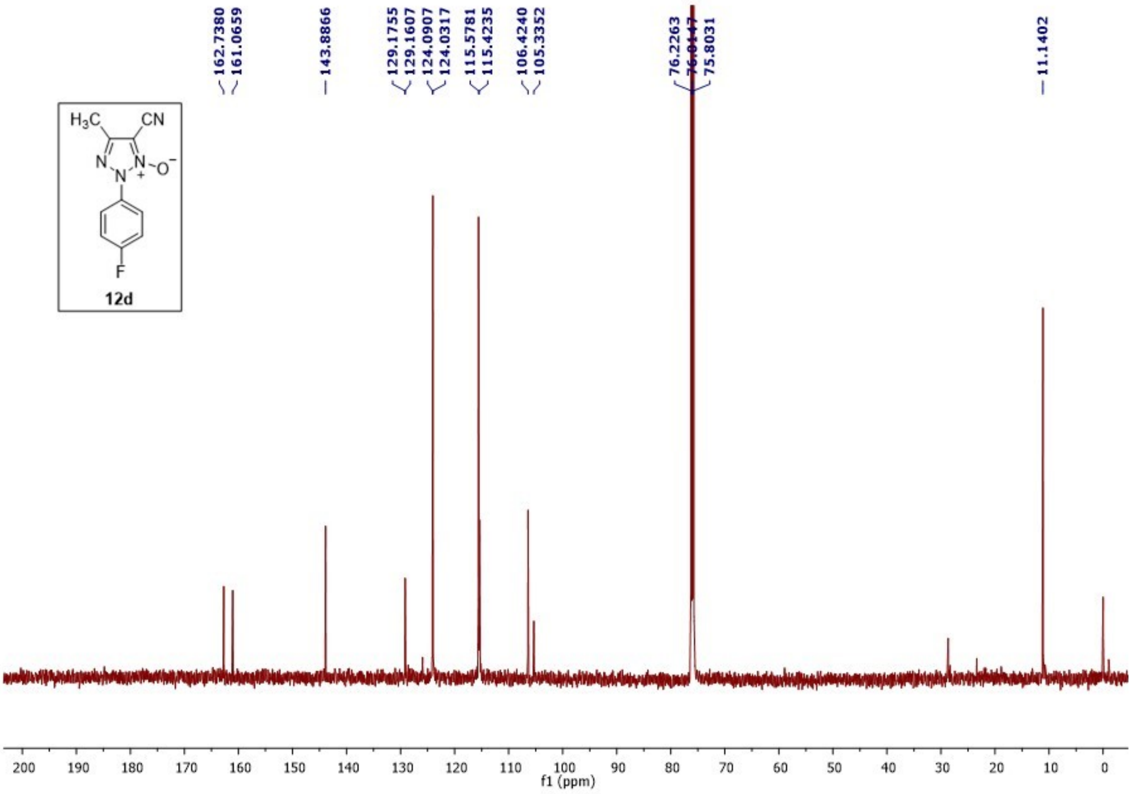

^{19}F NMR of Compound 12d

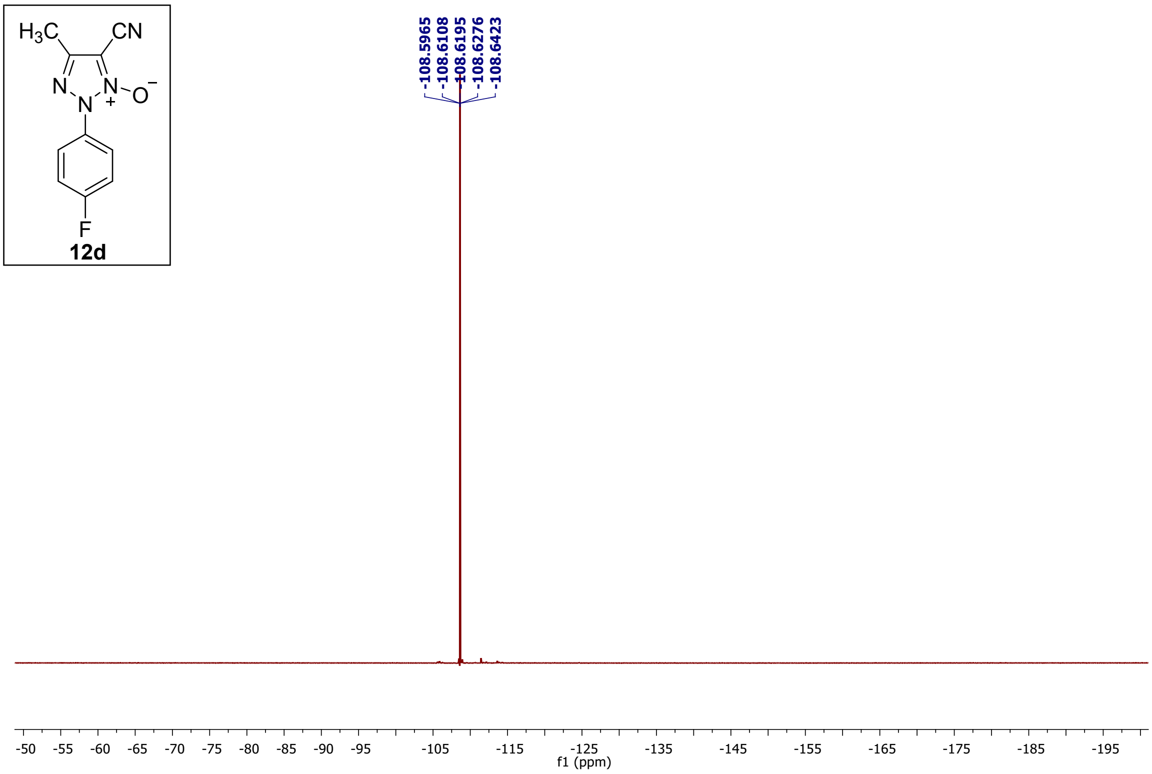

HRMS of Compound 12d

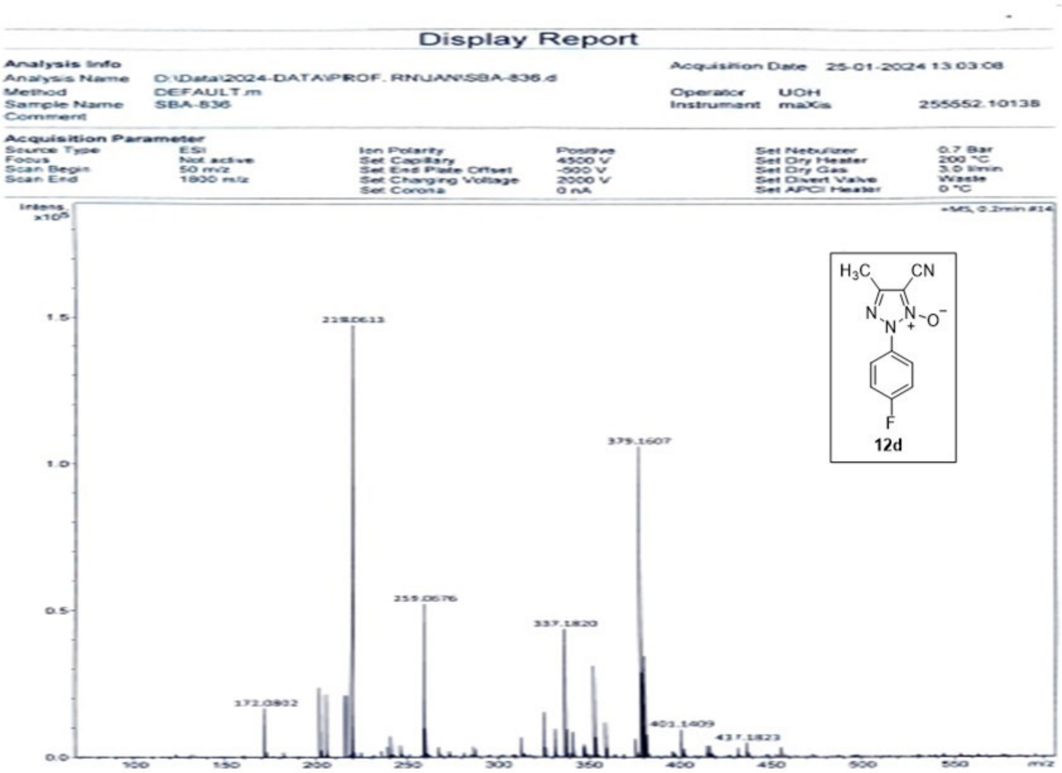

^{1}H NMR of Compound 12e

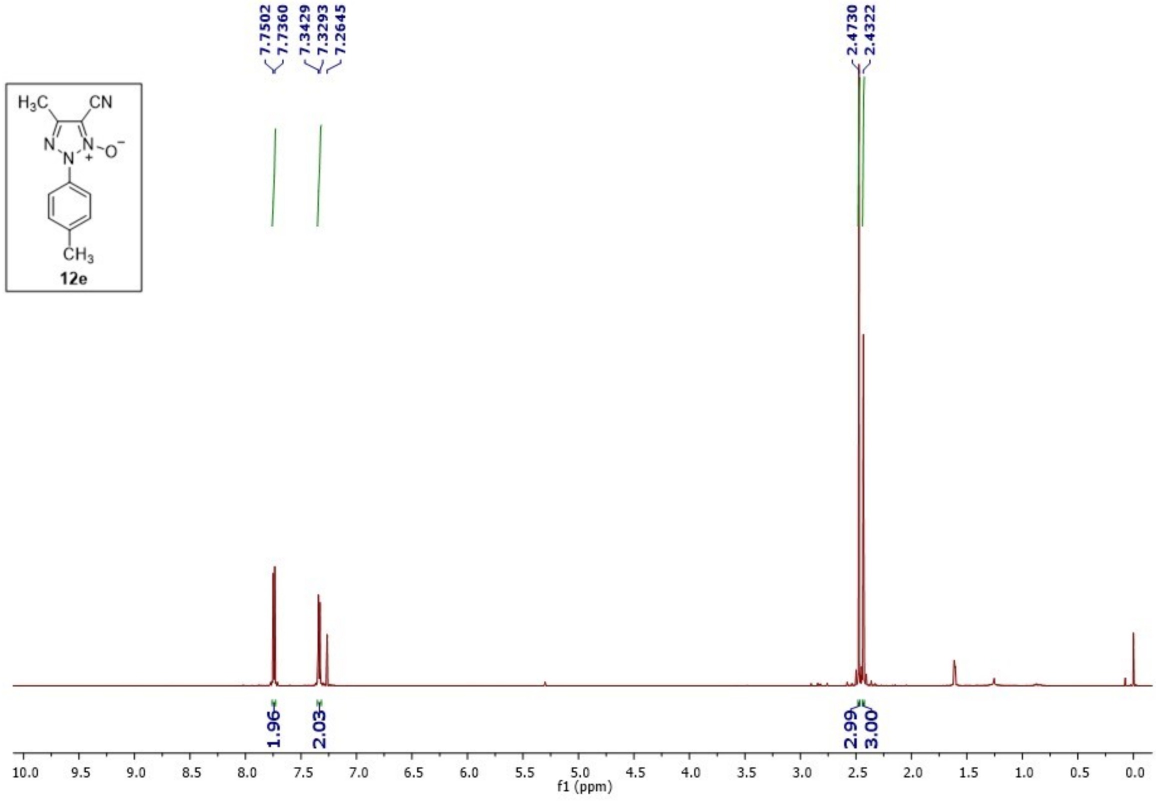

^{13}C NMR of Compound 12e

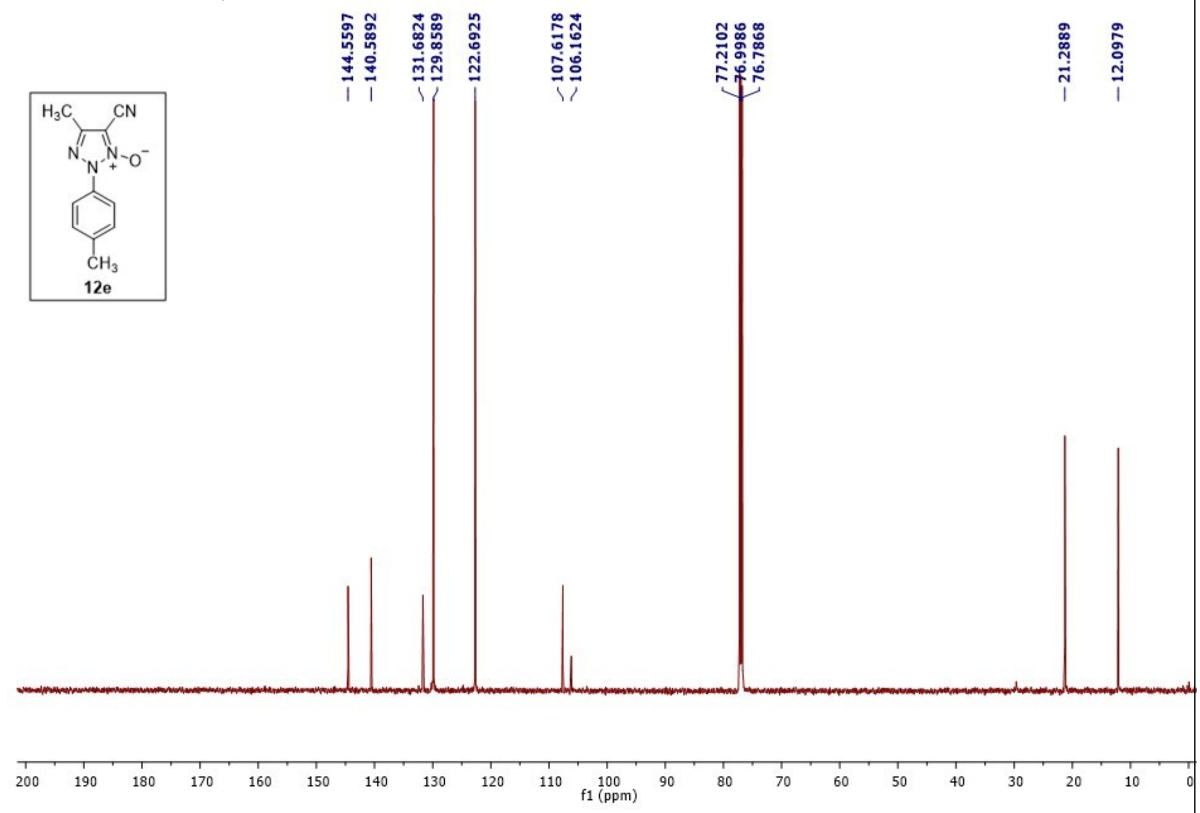

HRMS of Compound 12e

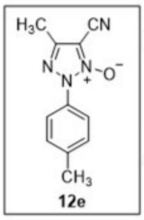

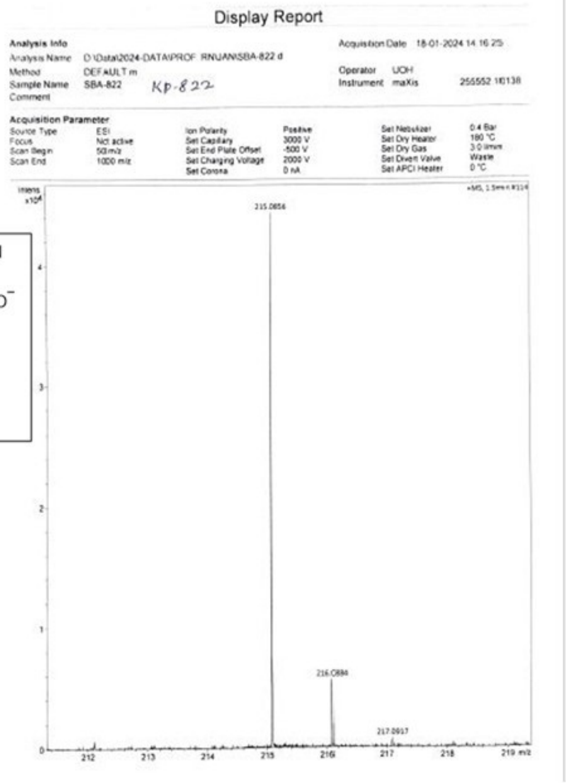

^{1}H NMR of Compound 12f

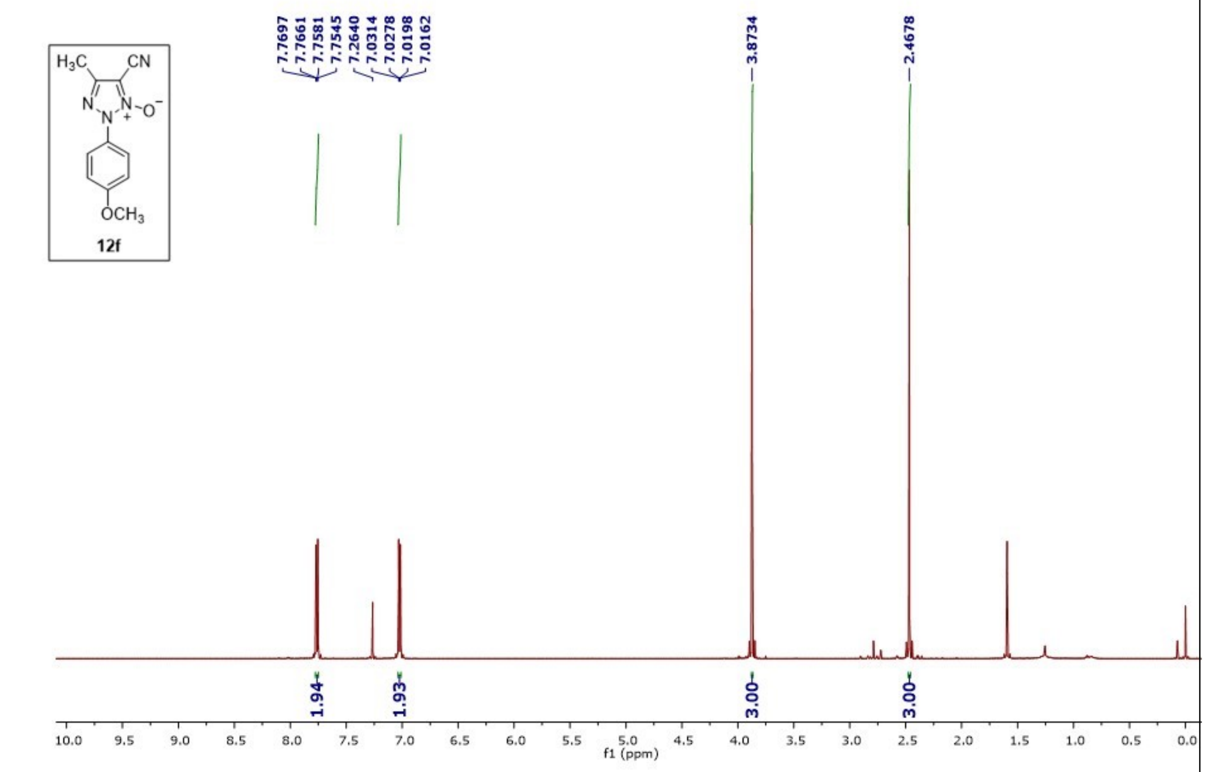

^{13}C NMR of Compound 12f

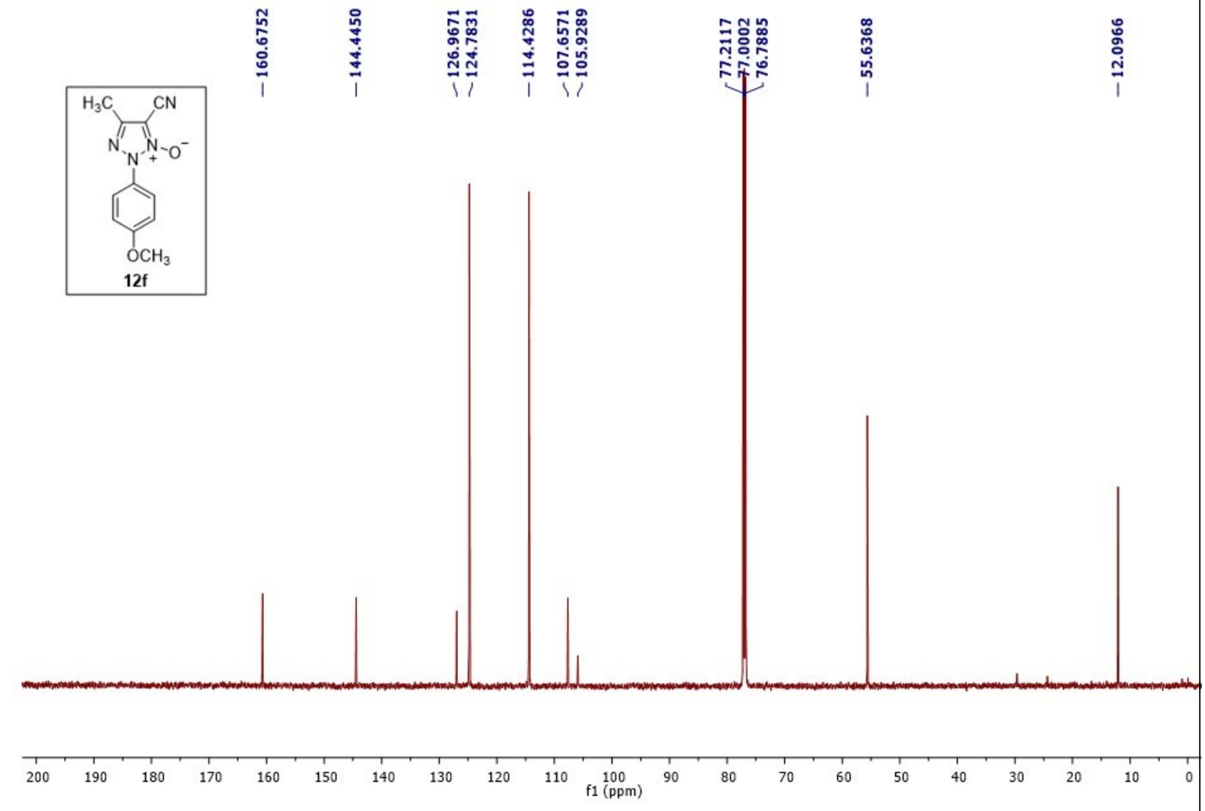

HRMS of Compound 12f

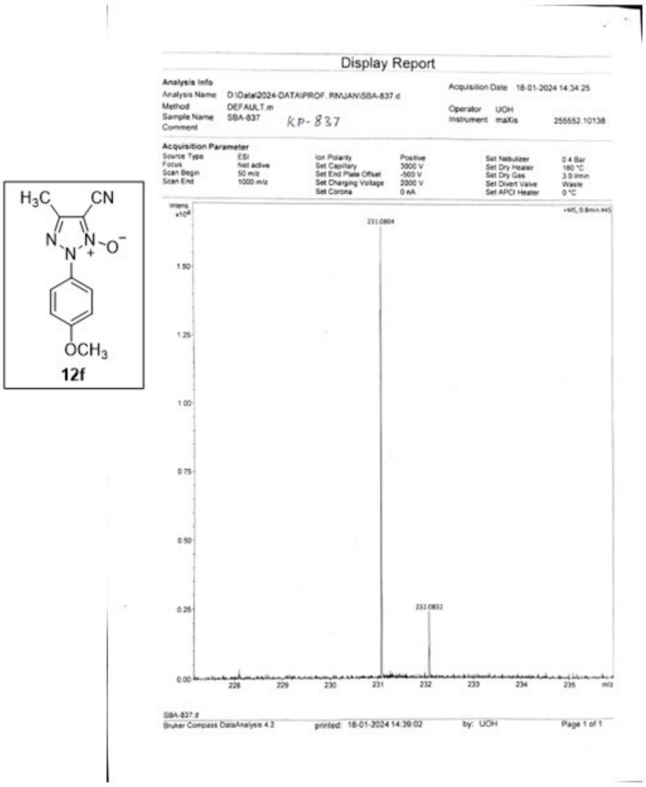

^1H NMR of Compound 12g

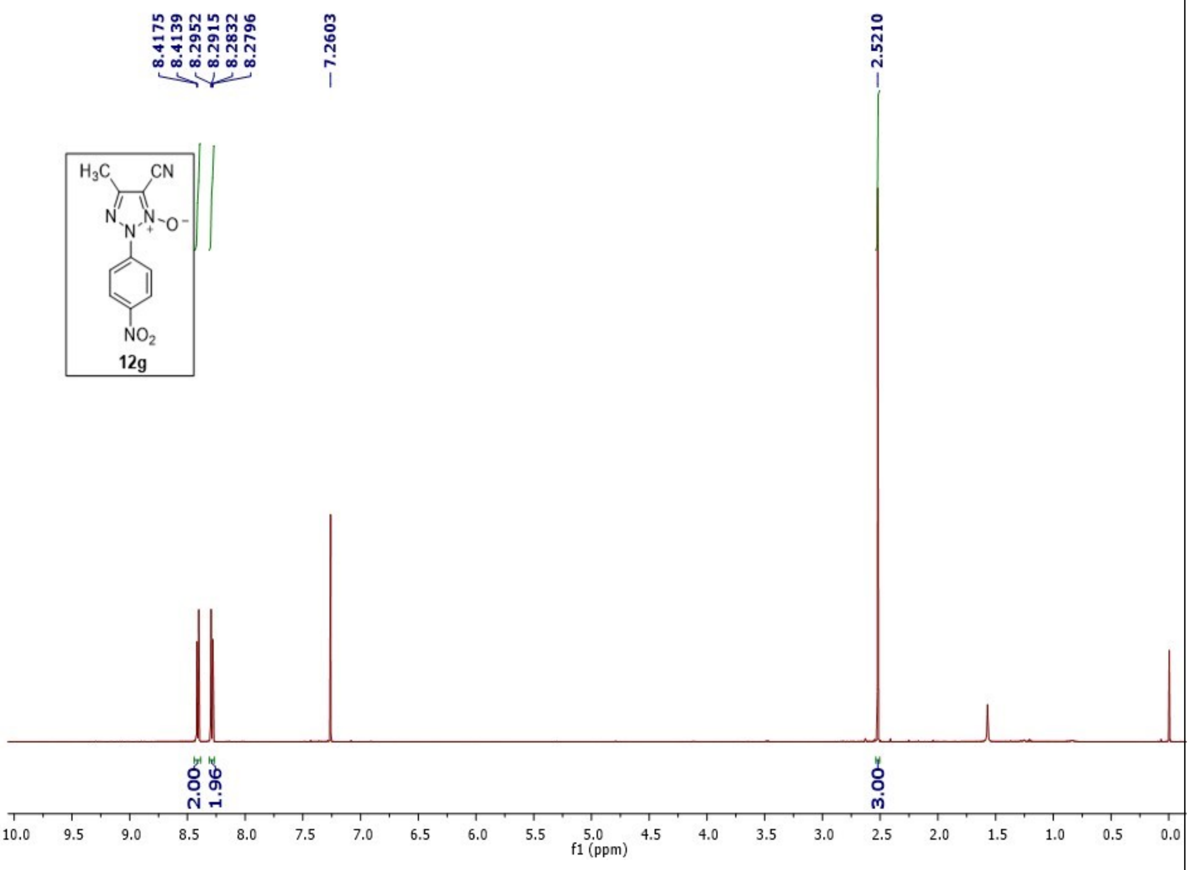

^{13}C NMR of compound 12g

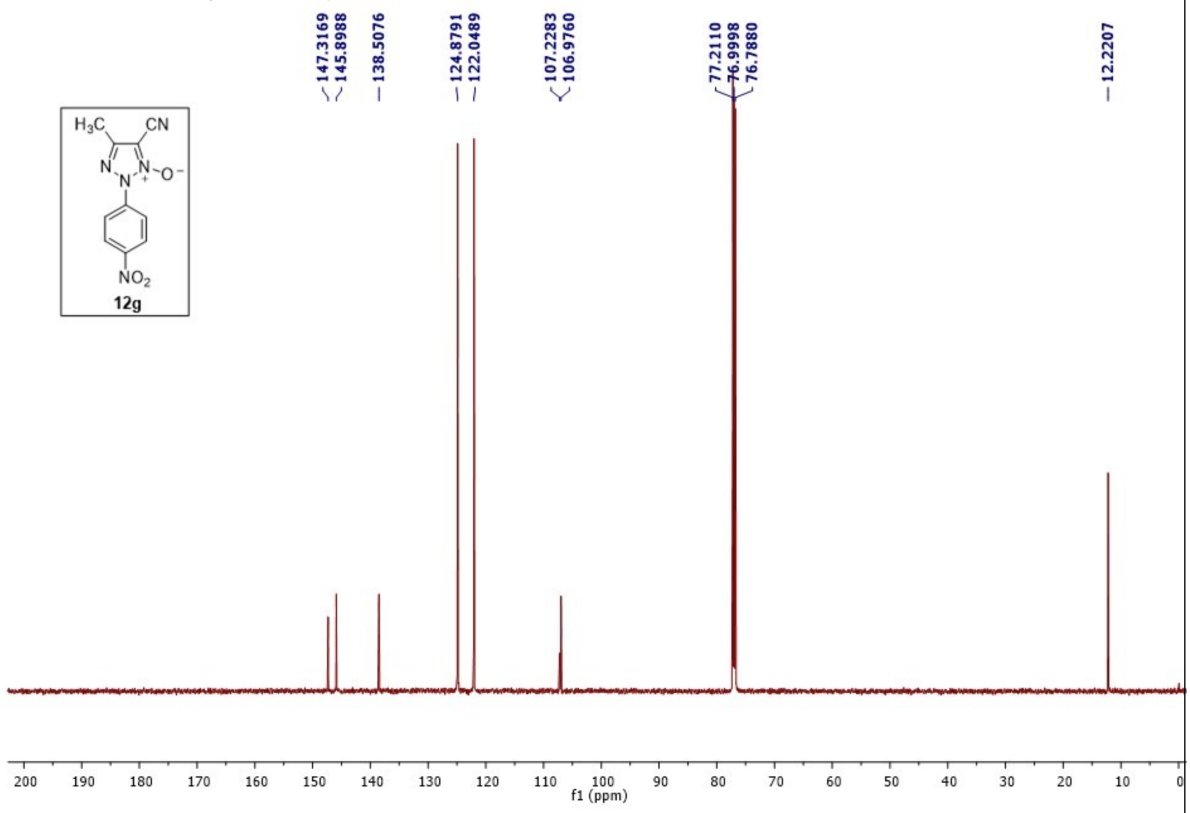

HRMS of Compound 12g

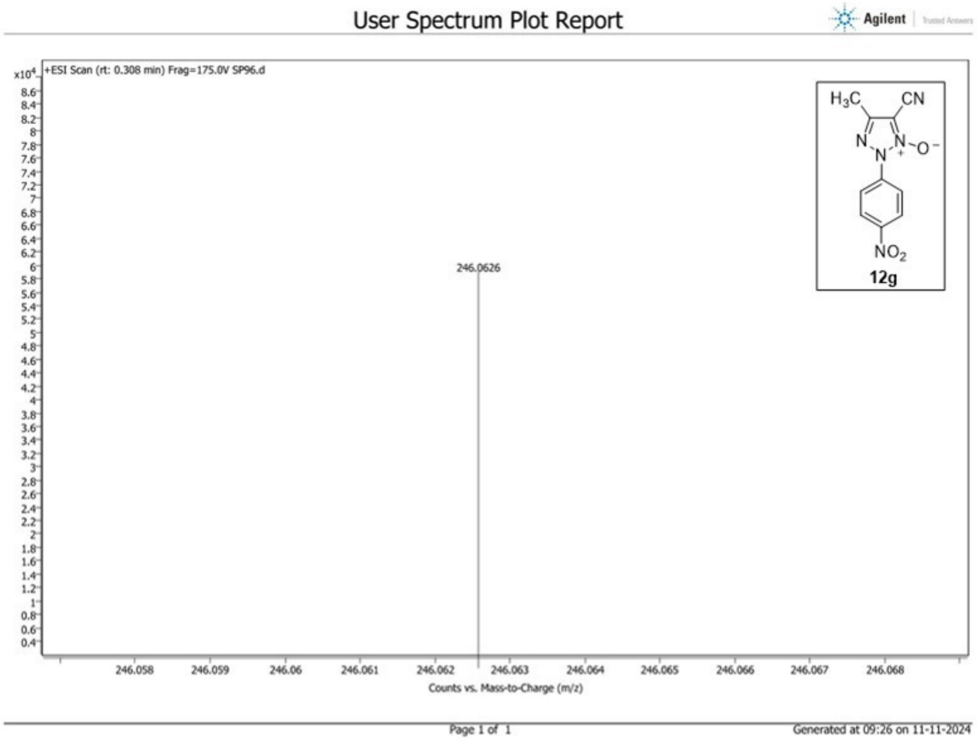

^{1}H NMR of Compound 12h

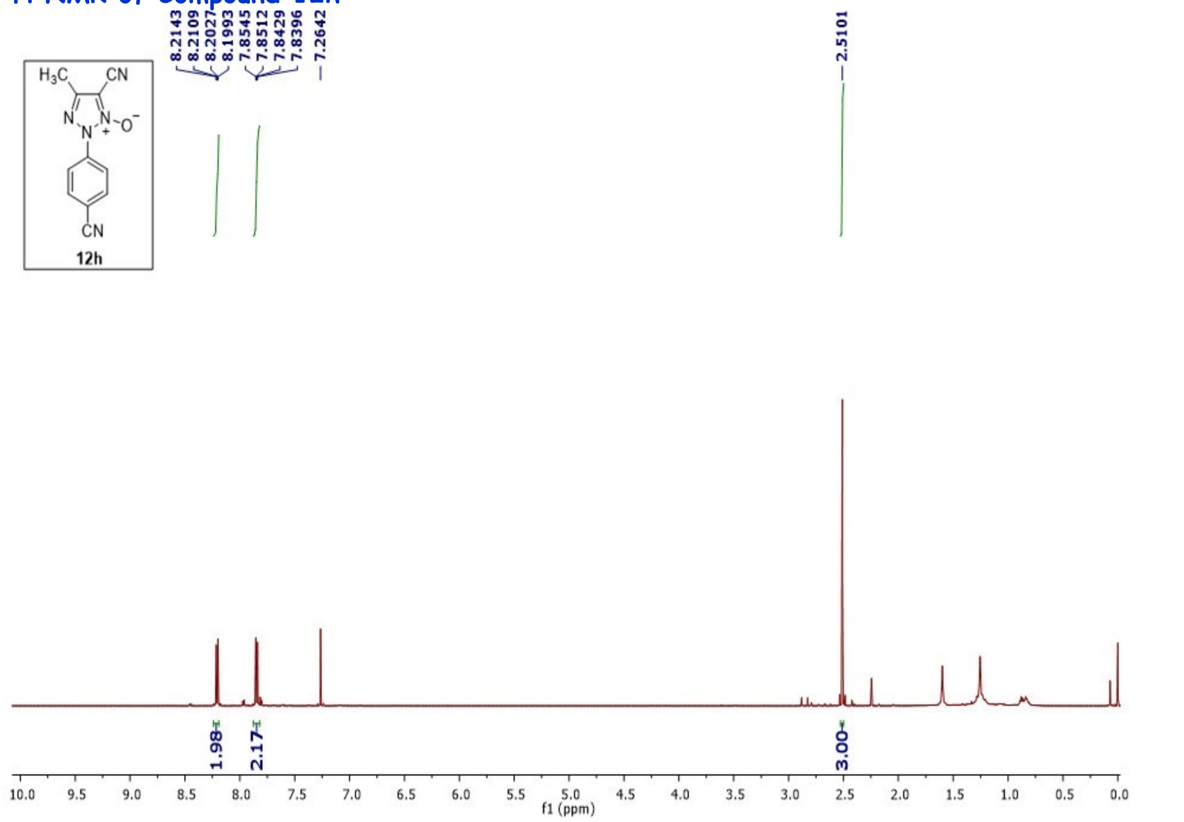

^{13}C NMR of Compound 12h

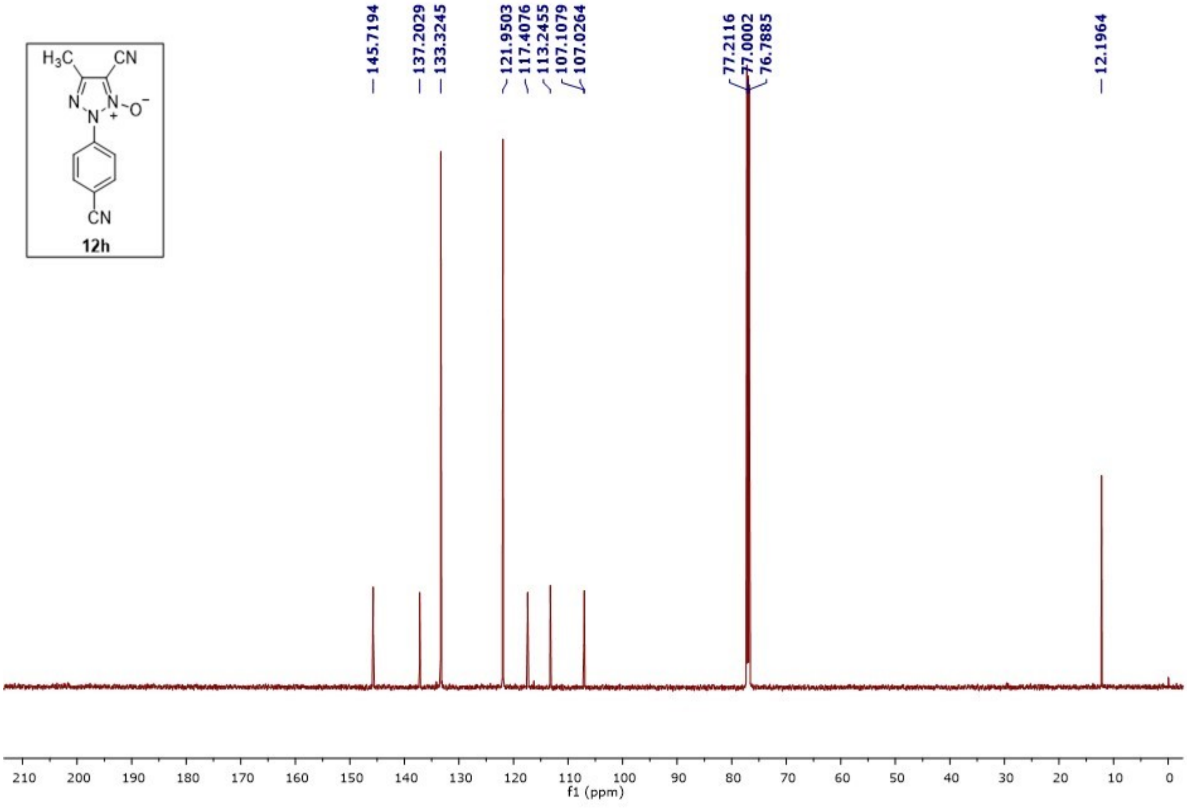

HRMS of Compound 12h

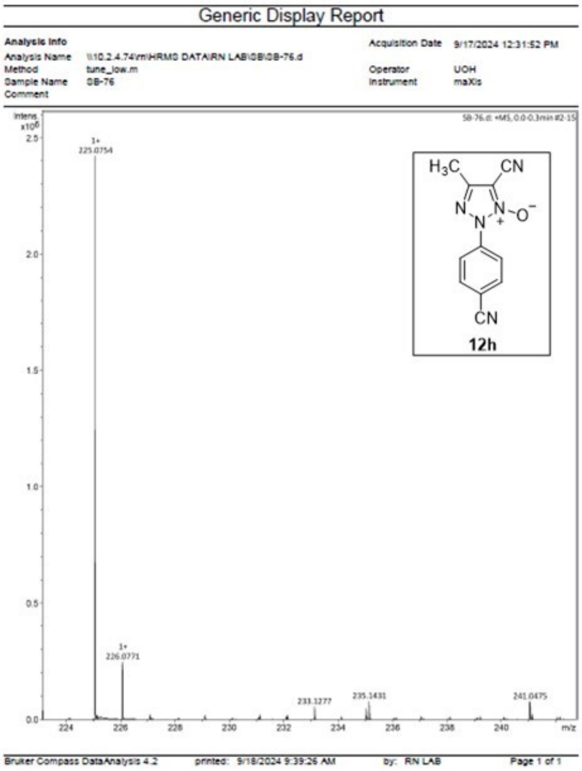

^{1}H NMR of Compound 12i

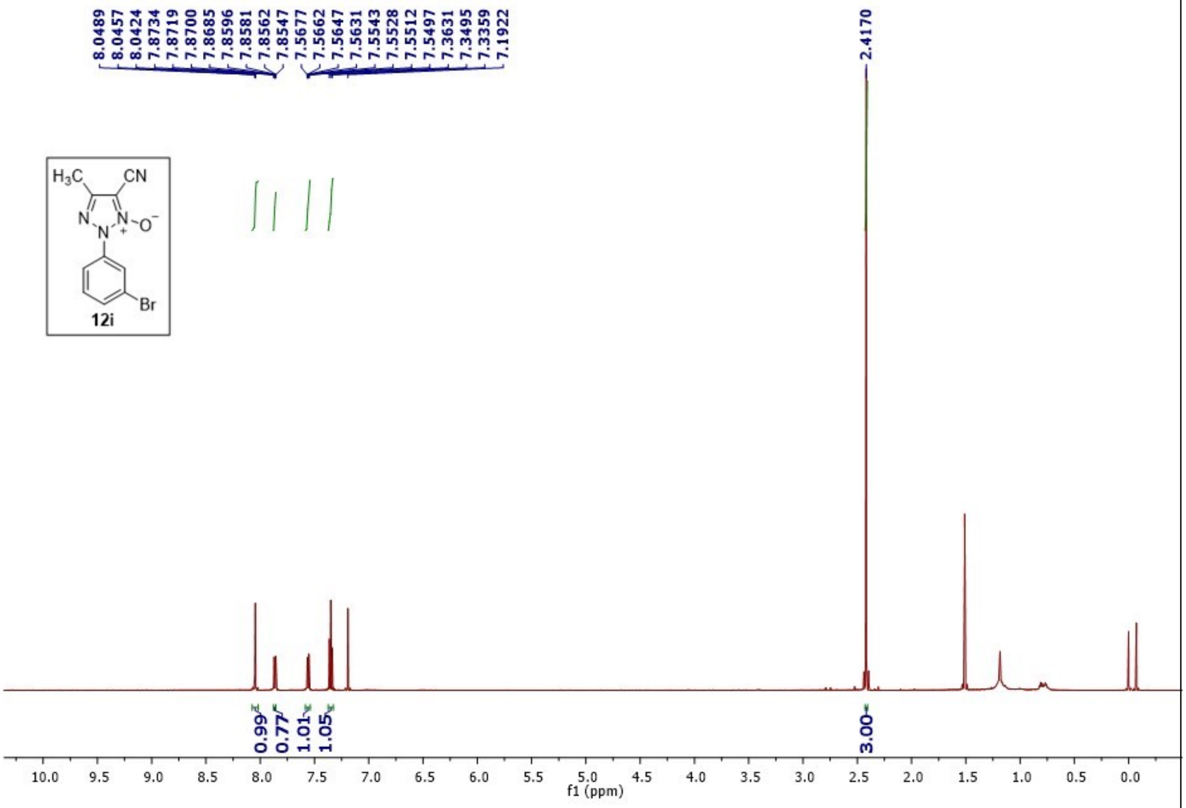

^{13}C NMR of Compound 12i

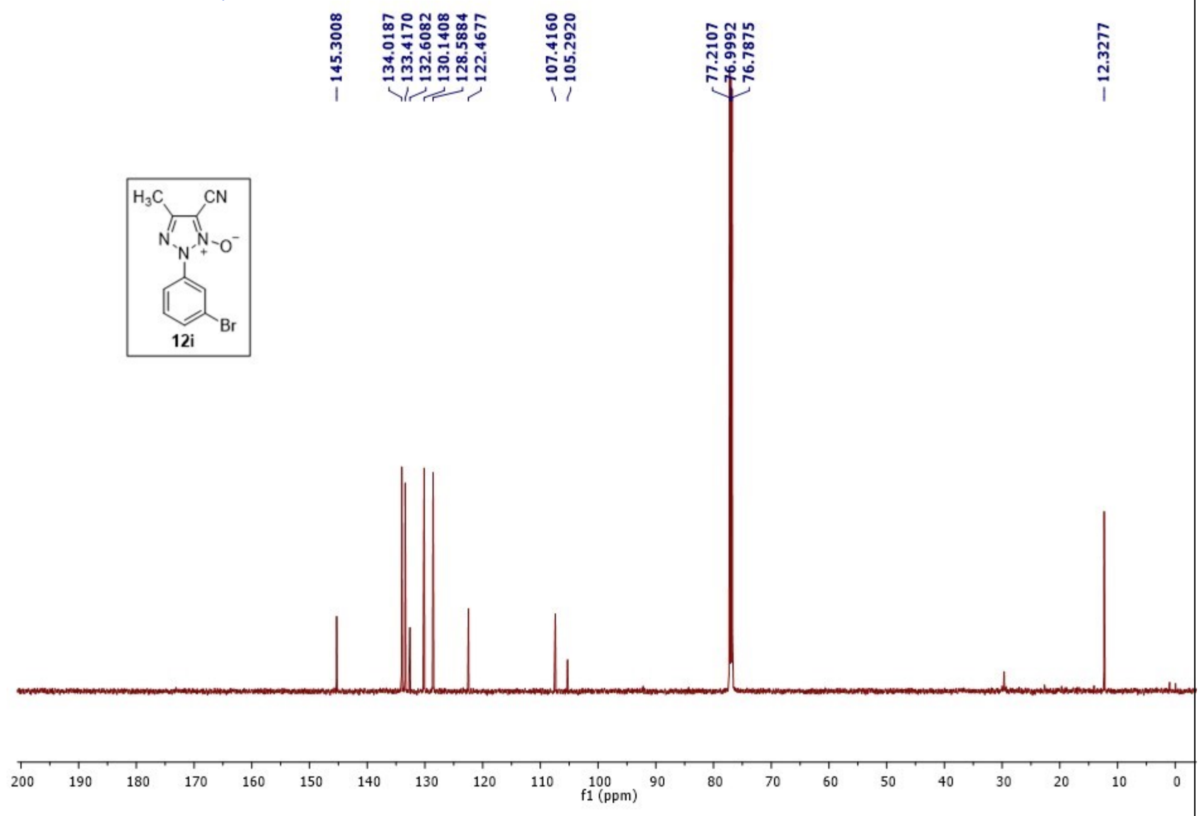

HRMS of Compound 12i

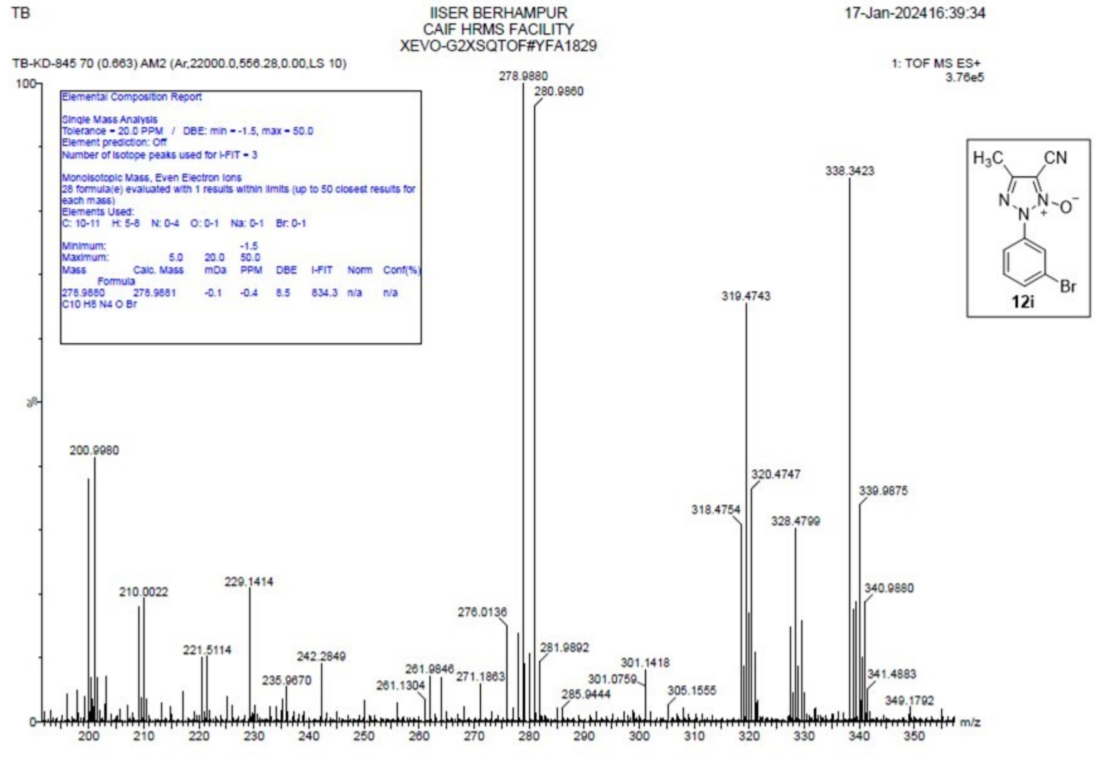

^{1}H NMR of Compound 12j

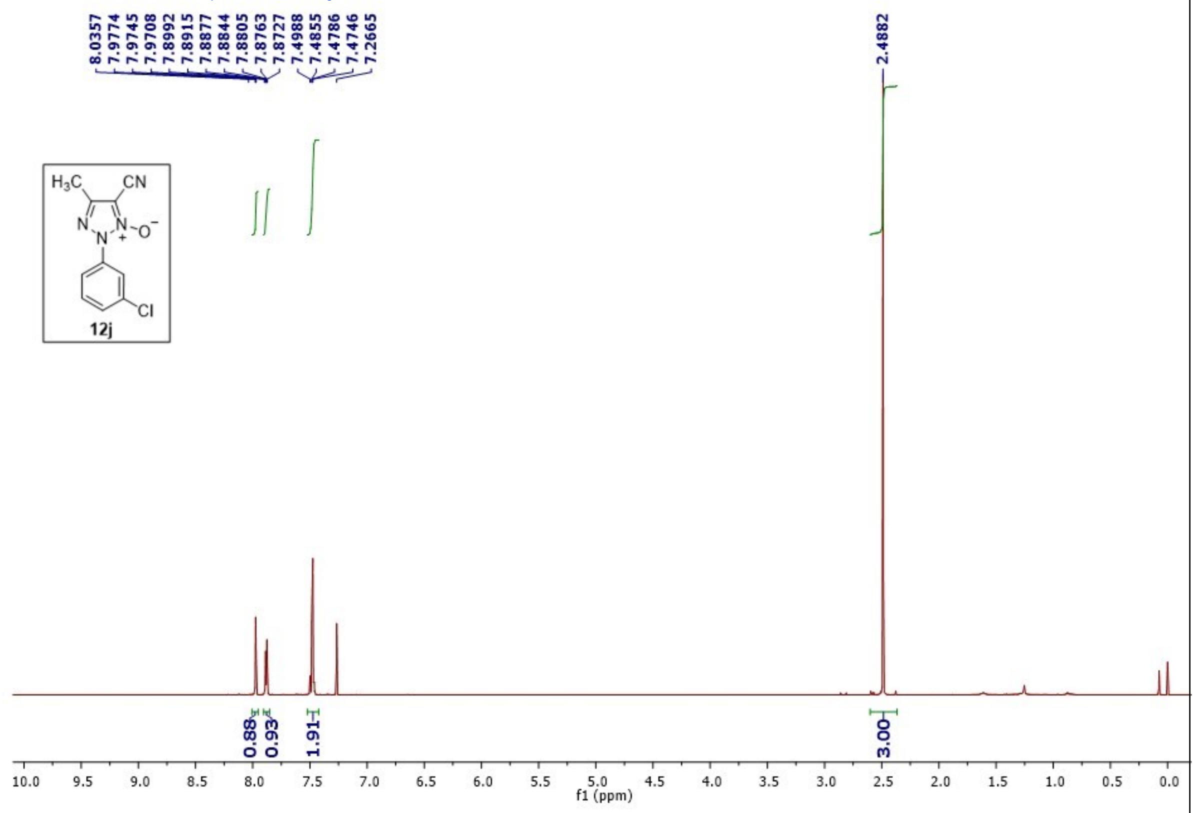

^{13}C NMR of Compound 12j

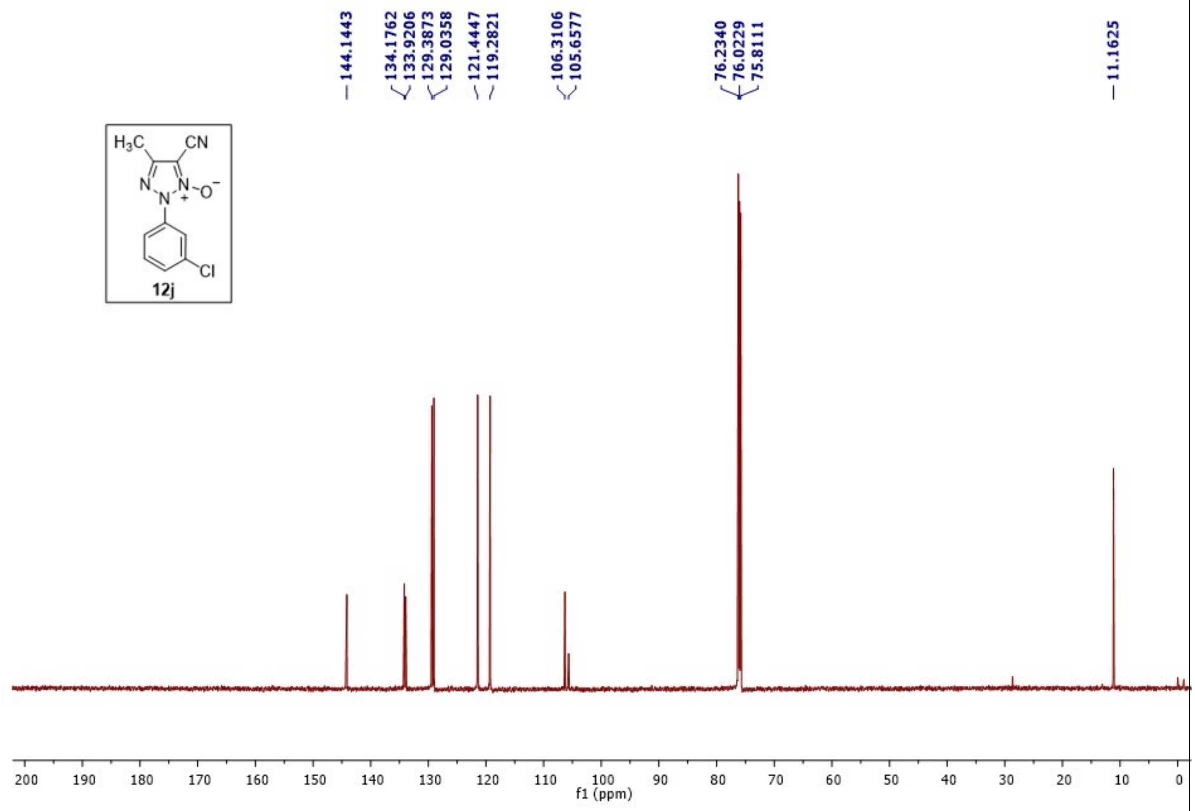

HRMS of Compound 12j

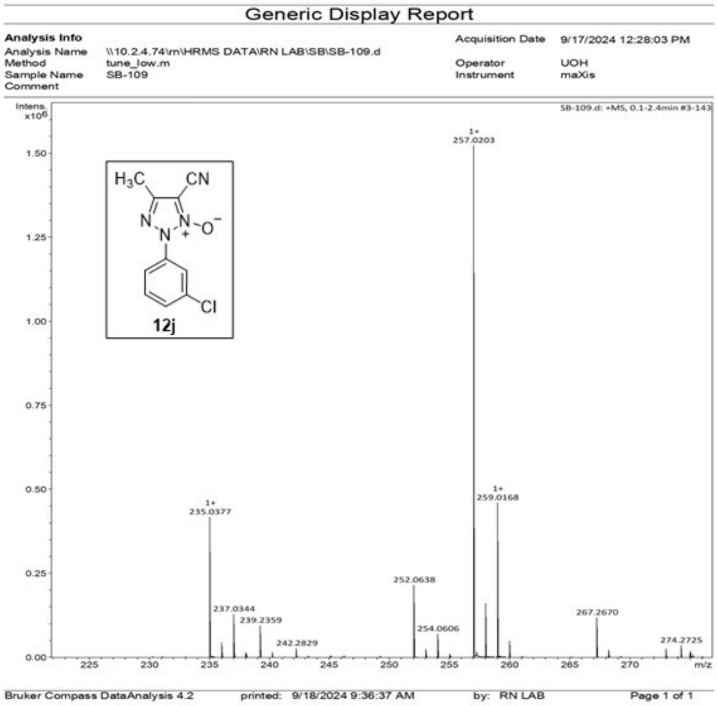

^{1}H NMR of Compound 12k

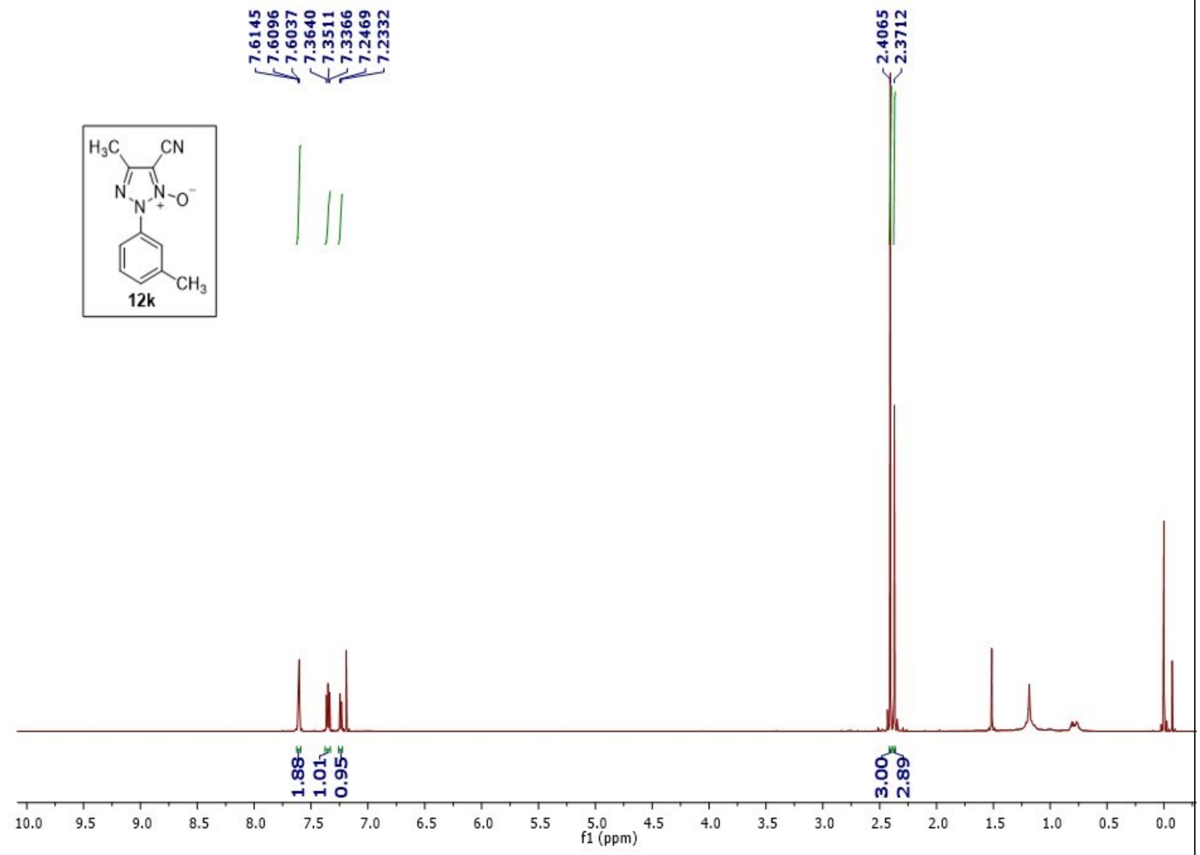

^{13}C NMR of Compound 12k

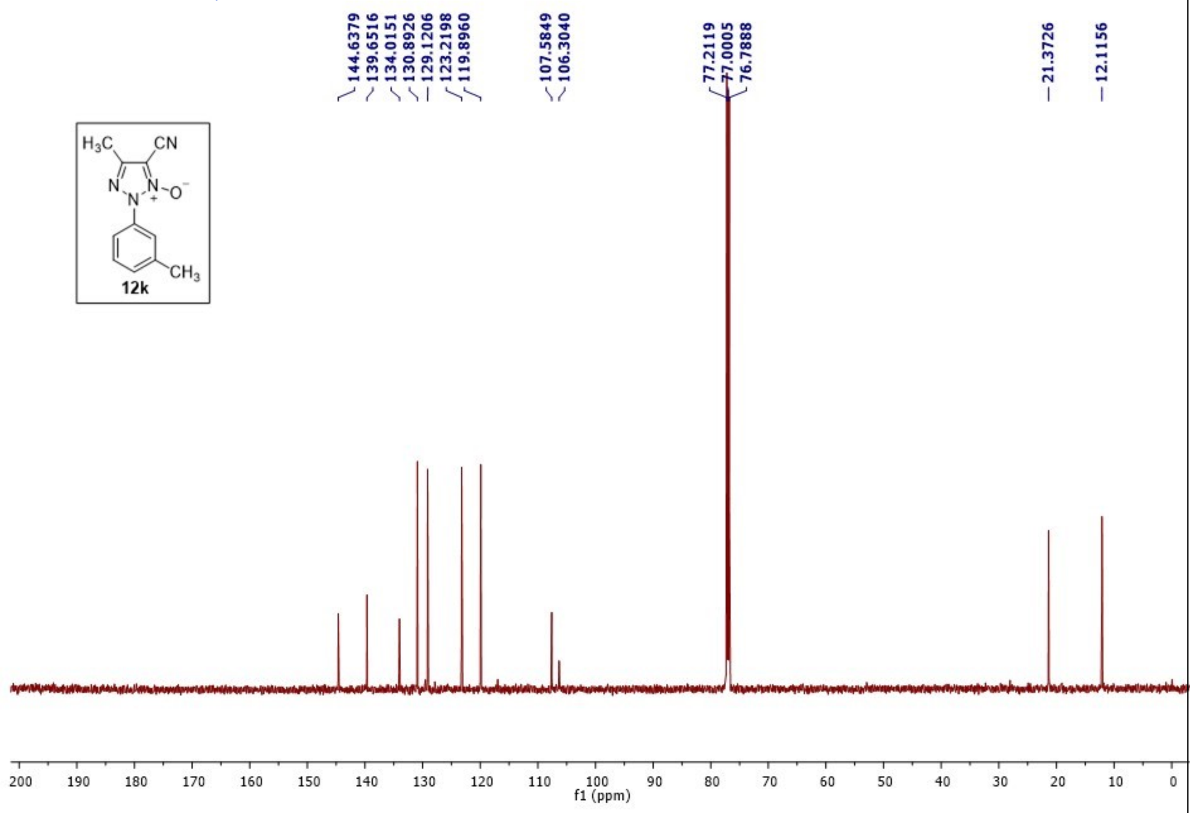

HRMS of Compound 12k

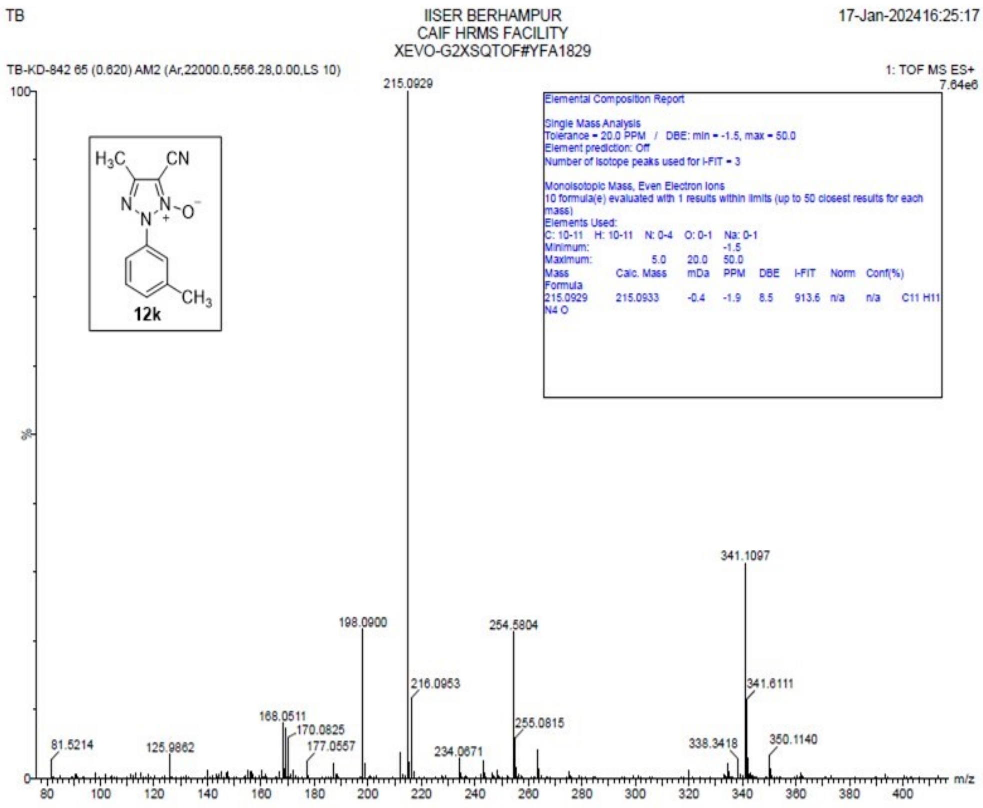

^{1}H NMR of Compound 12l

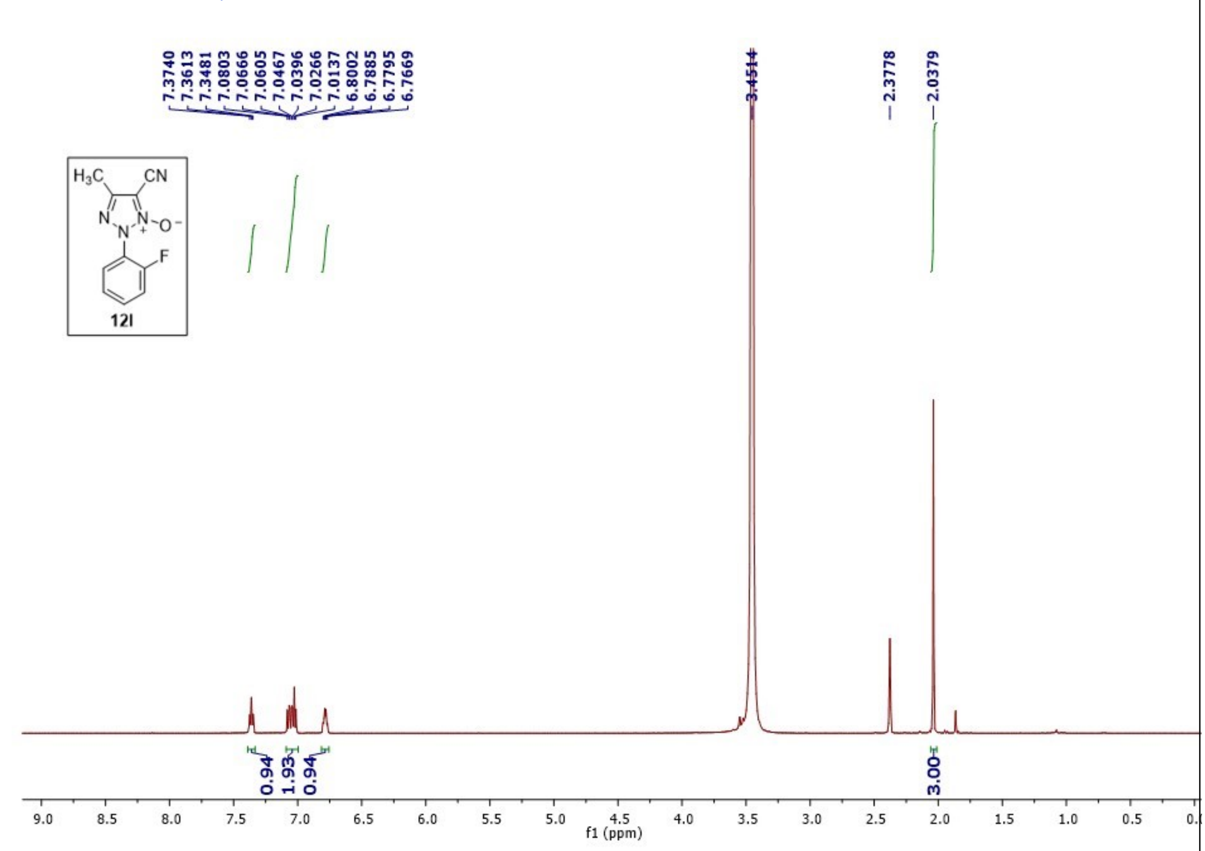

^{13}C NMR of Compound 12l

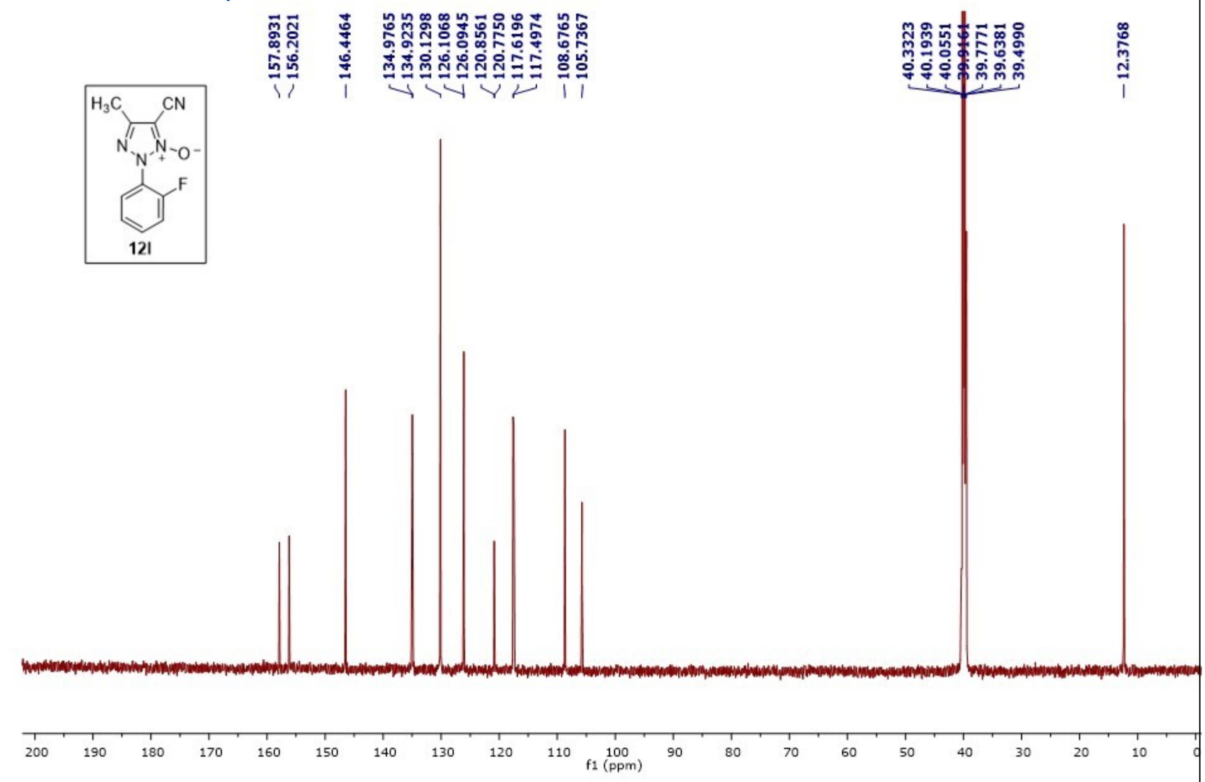

^{19}F NMR of Compound 12l

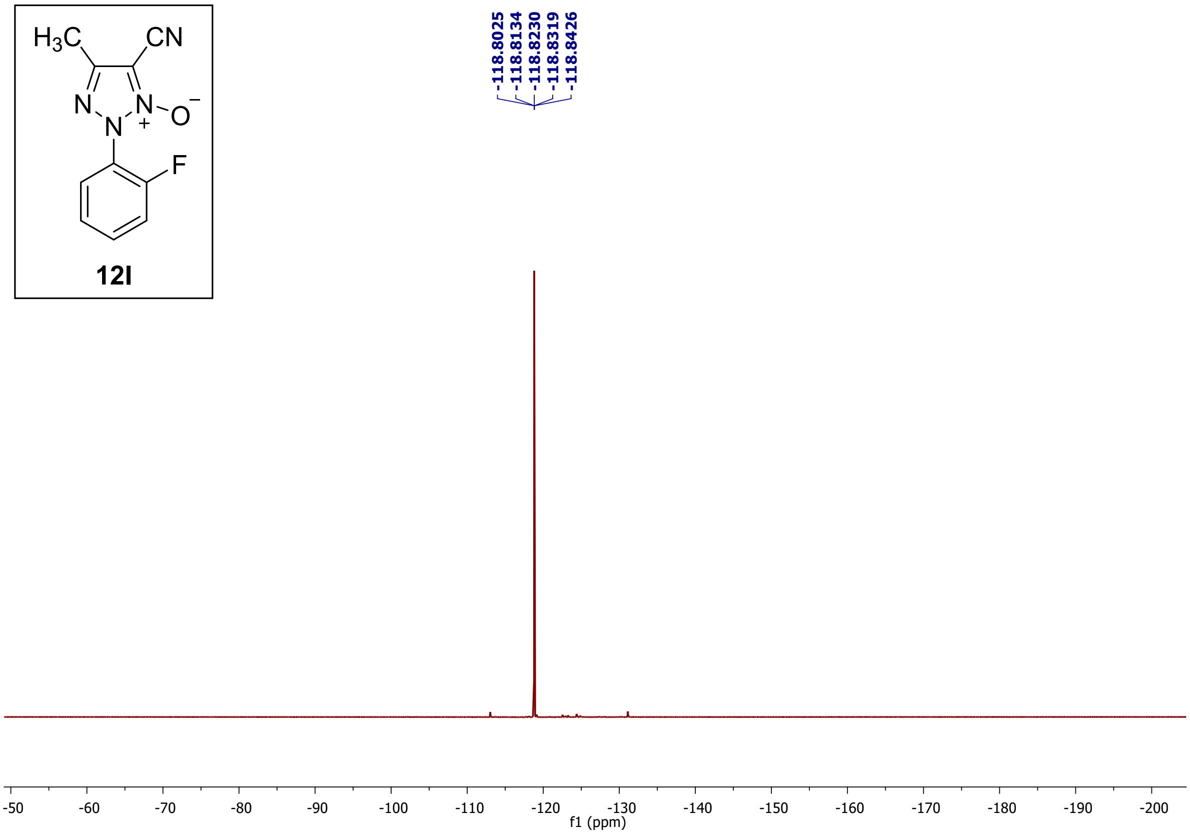

HRMS of Compound 12l

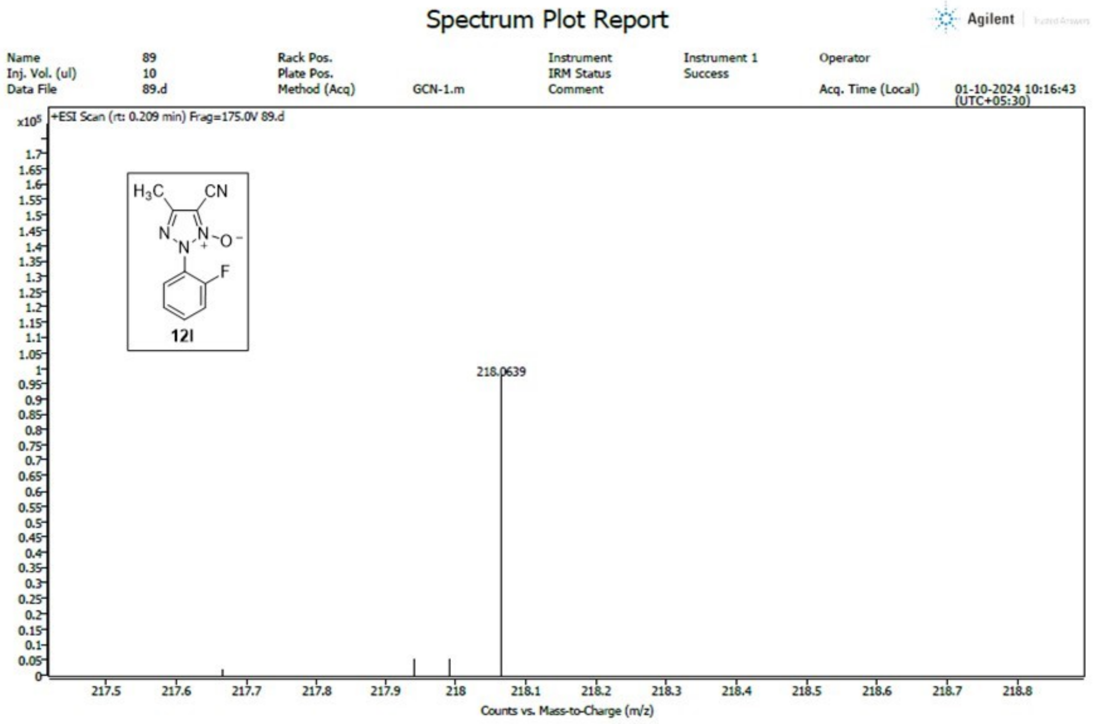

^{1}H NMR of Compound 12m

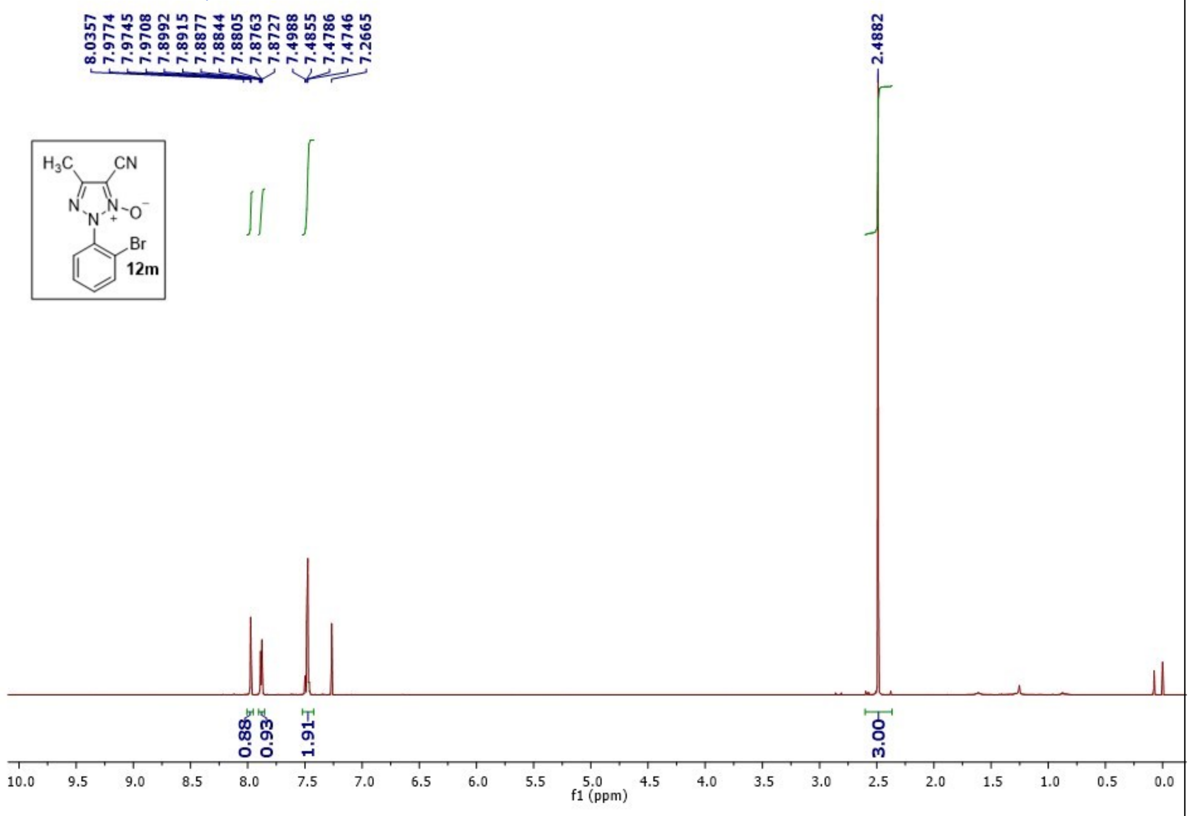

^{13}C NMR of Compound 12m

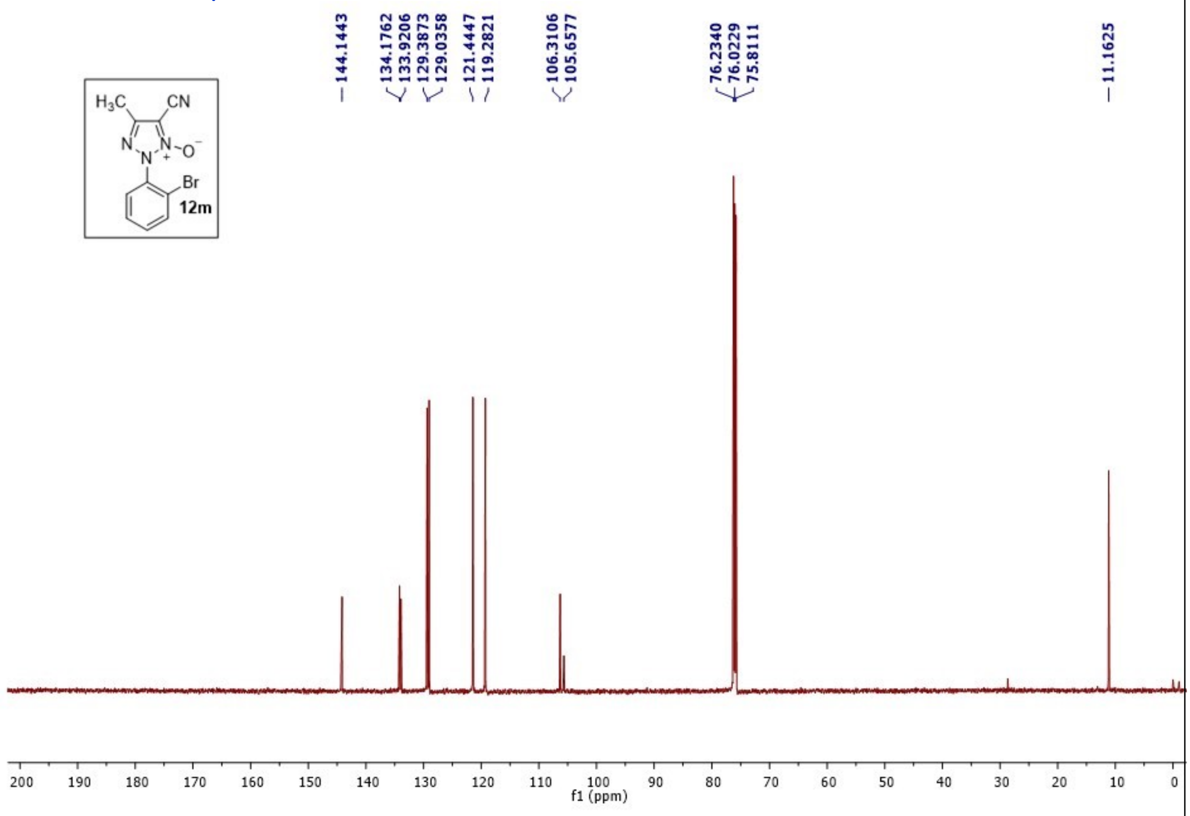

HRMS of Compound 12m

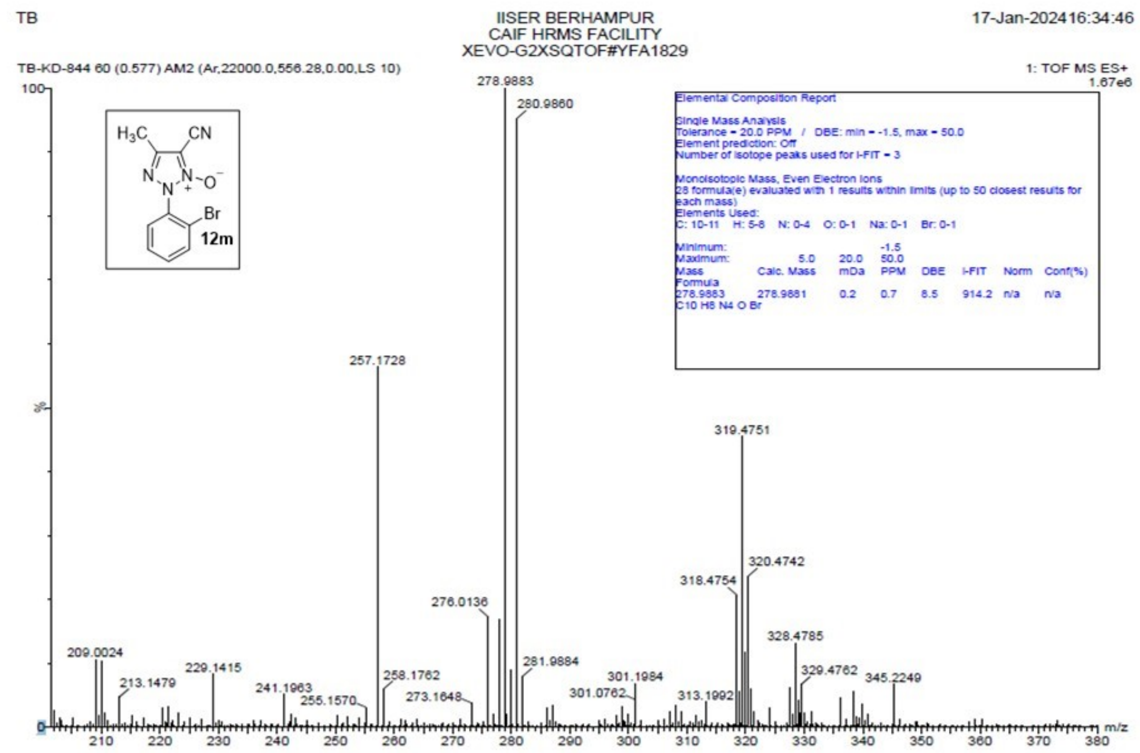

¹H NMR of compound 12n

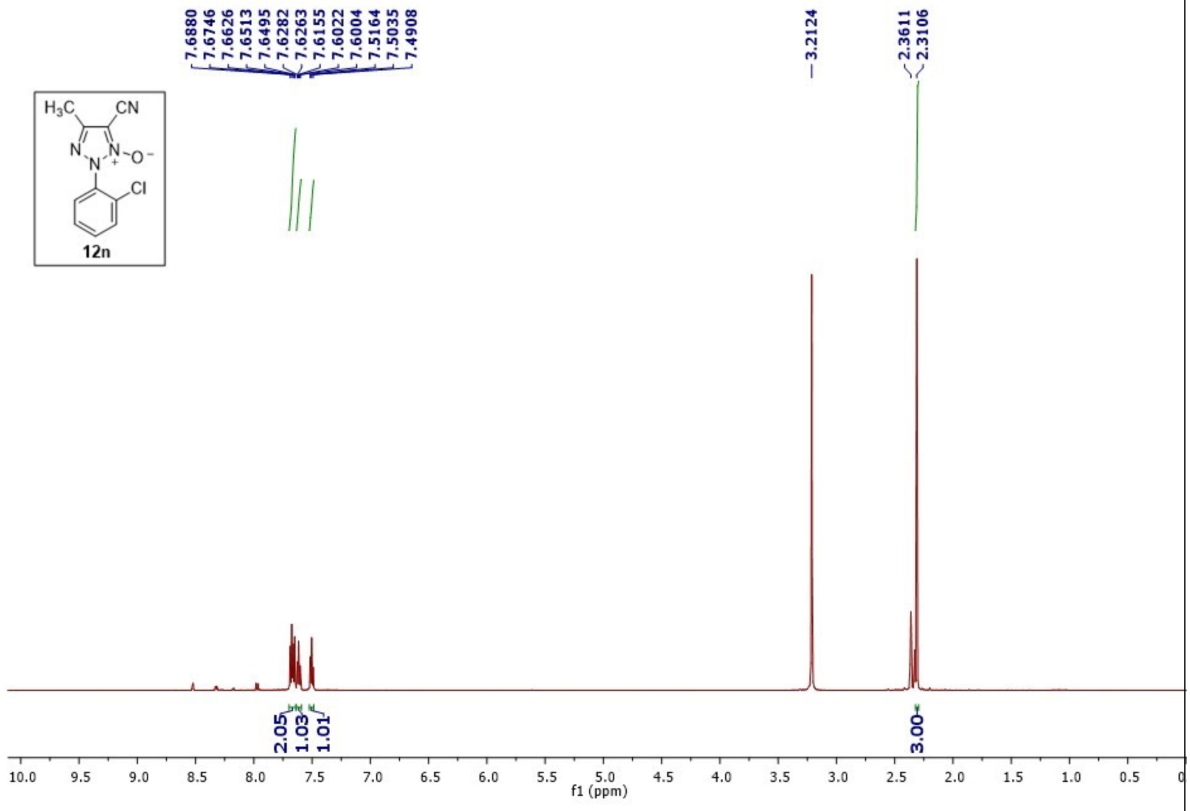

^{13}C NMR of Compound 12n

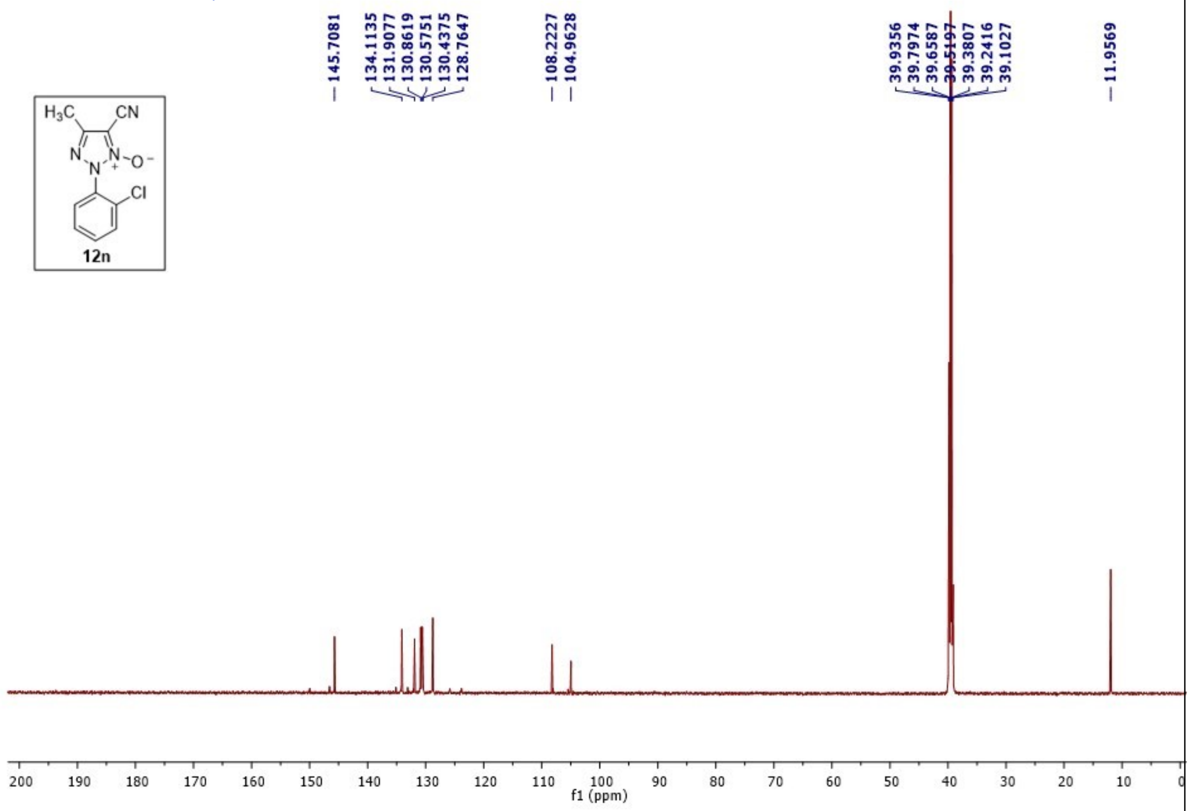

HRMS of Compound 12n

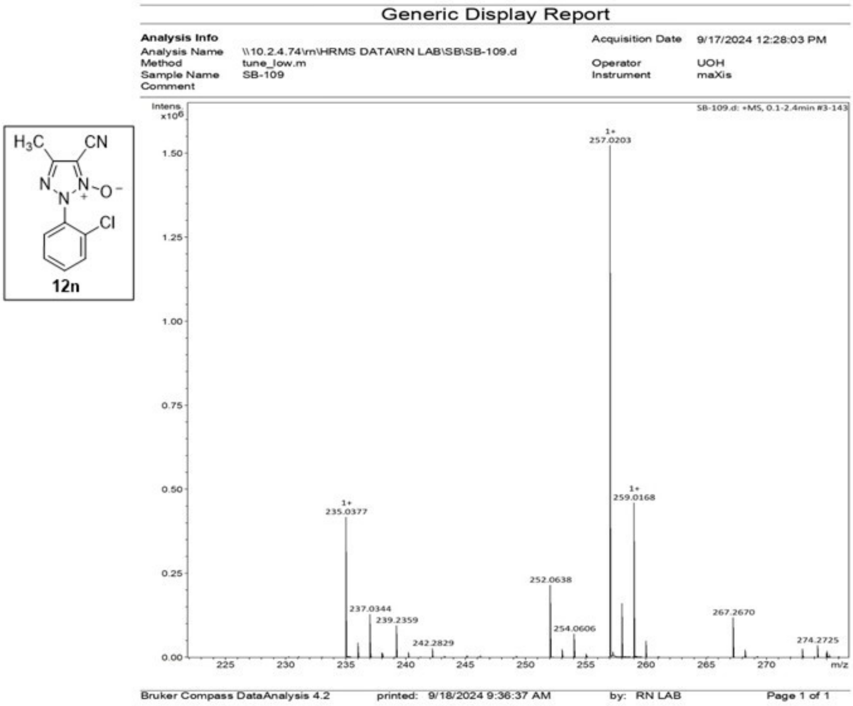

^{1}H NMR of Compound 12o

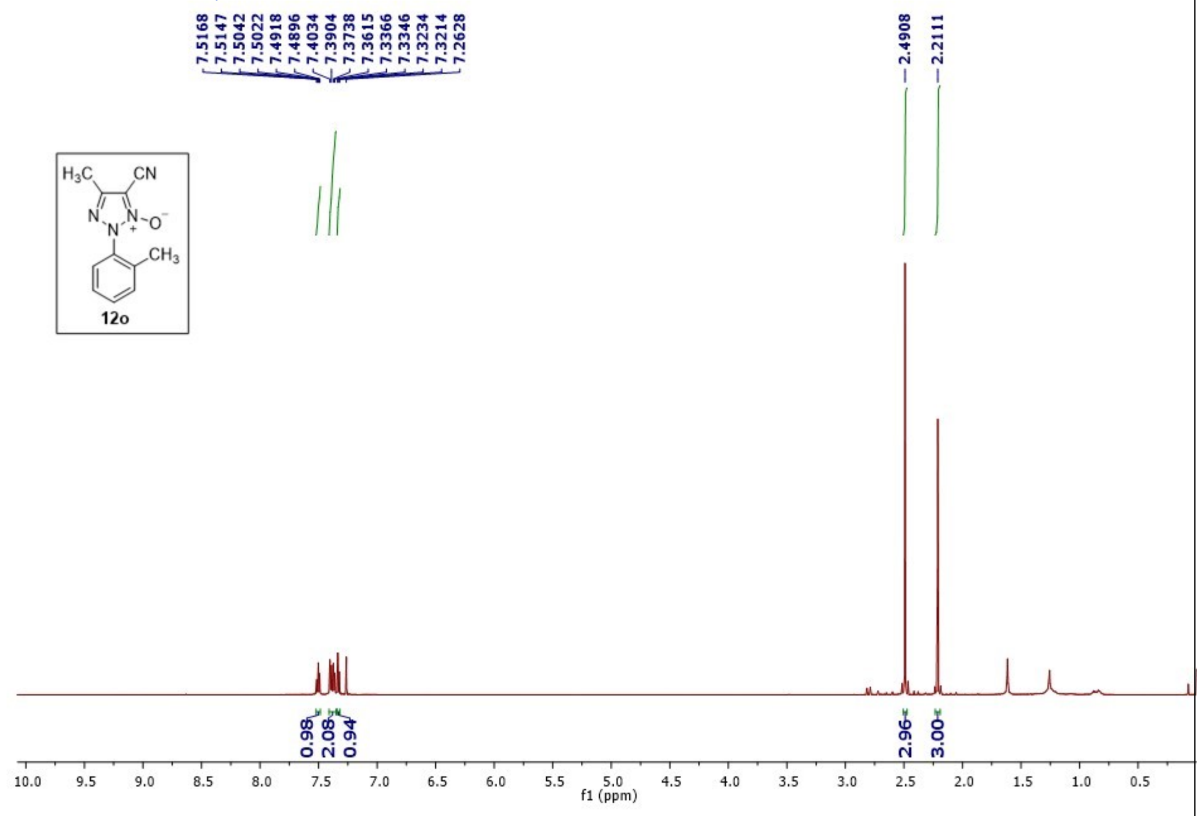

^{13}C NMR of Compound 12o

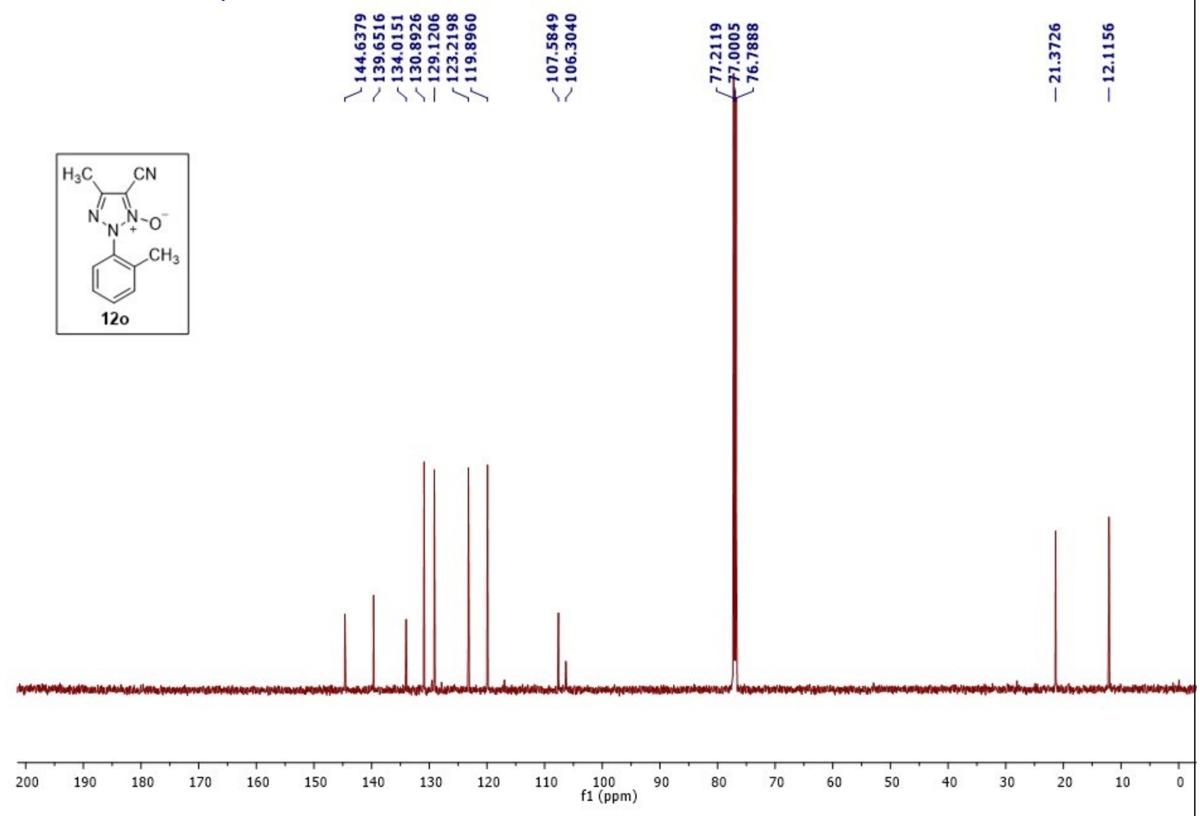

HRMS of Compound 12o

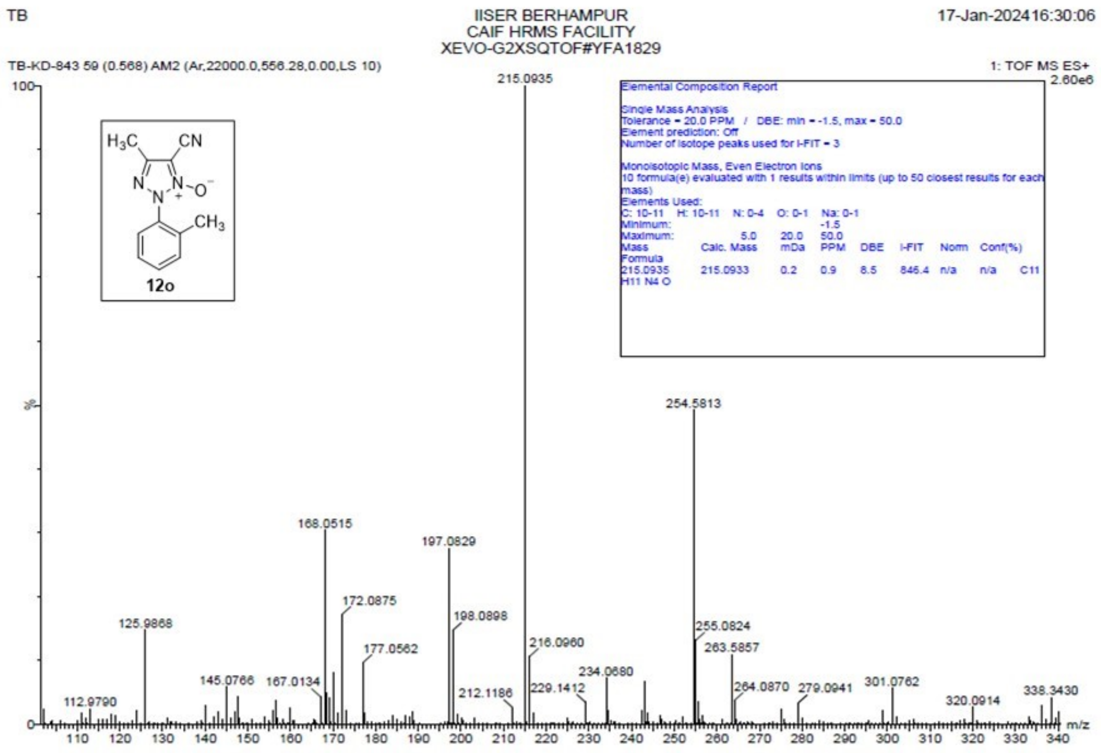

¹H NMR of Compound 12p

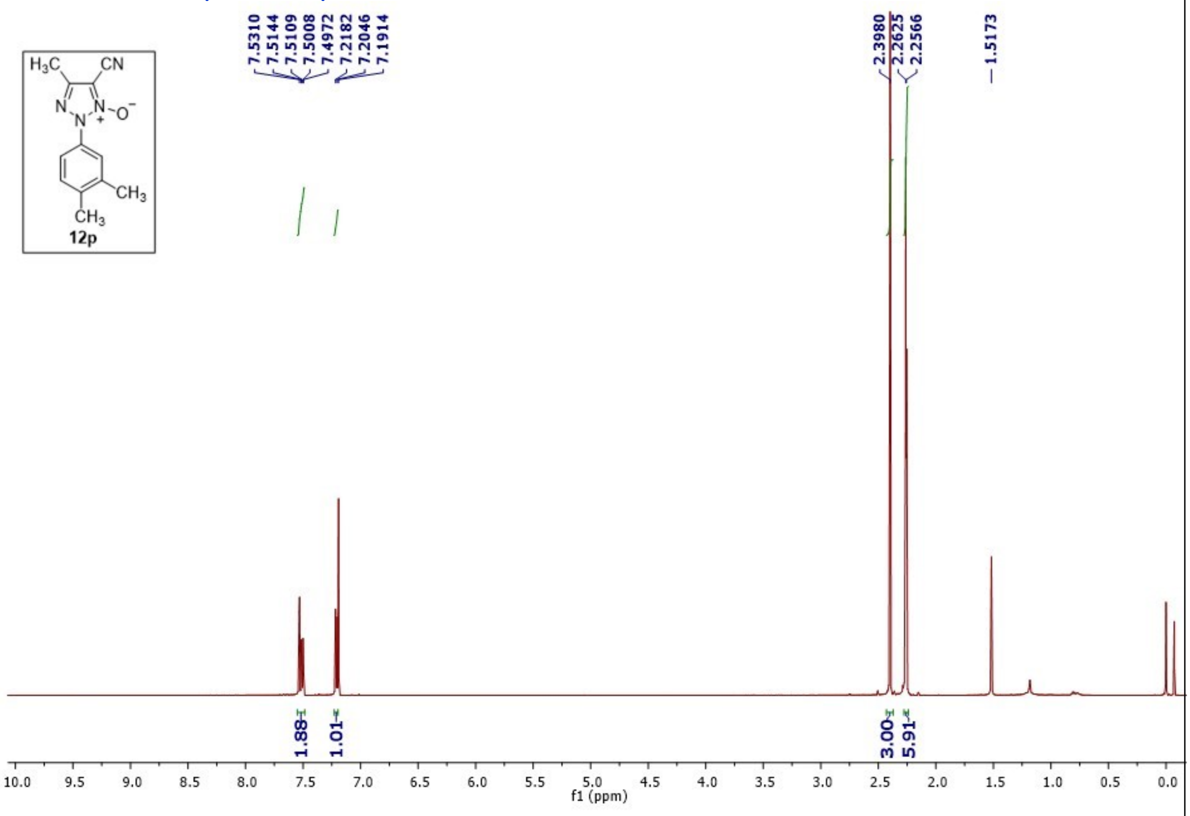

^{13}C NMR of Compound 12p

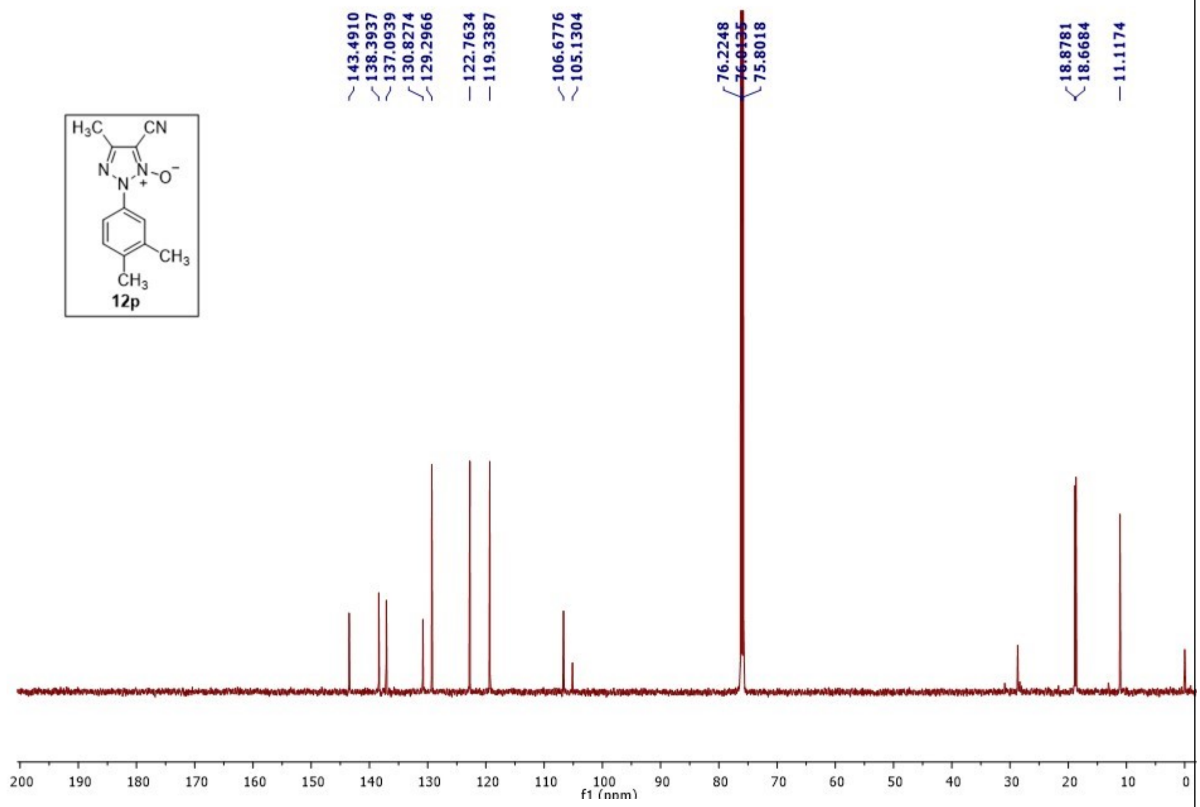

HRMS of Compound 12p

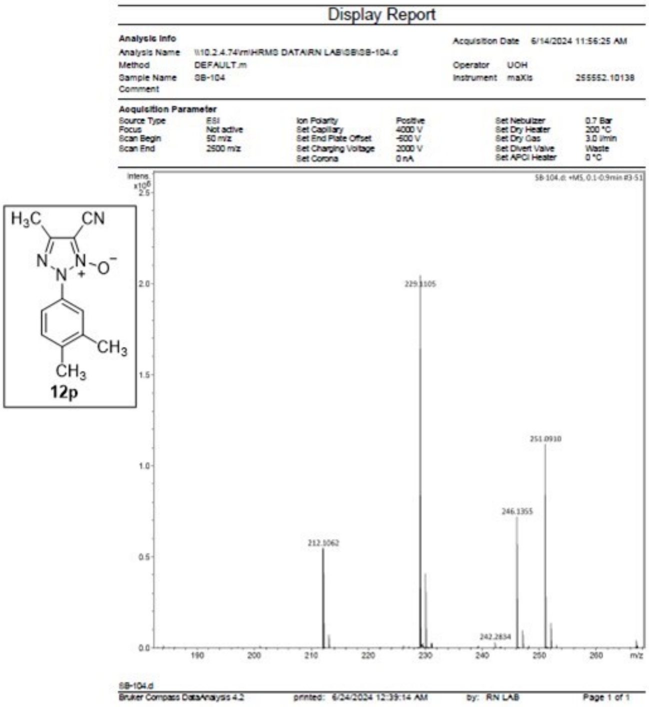

^{1}H NMR of Compound 12t

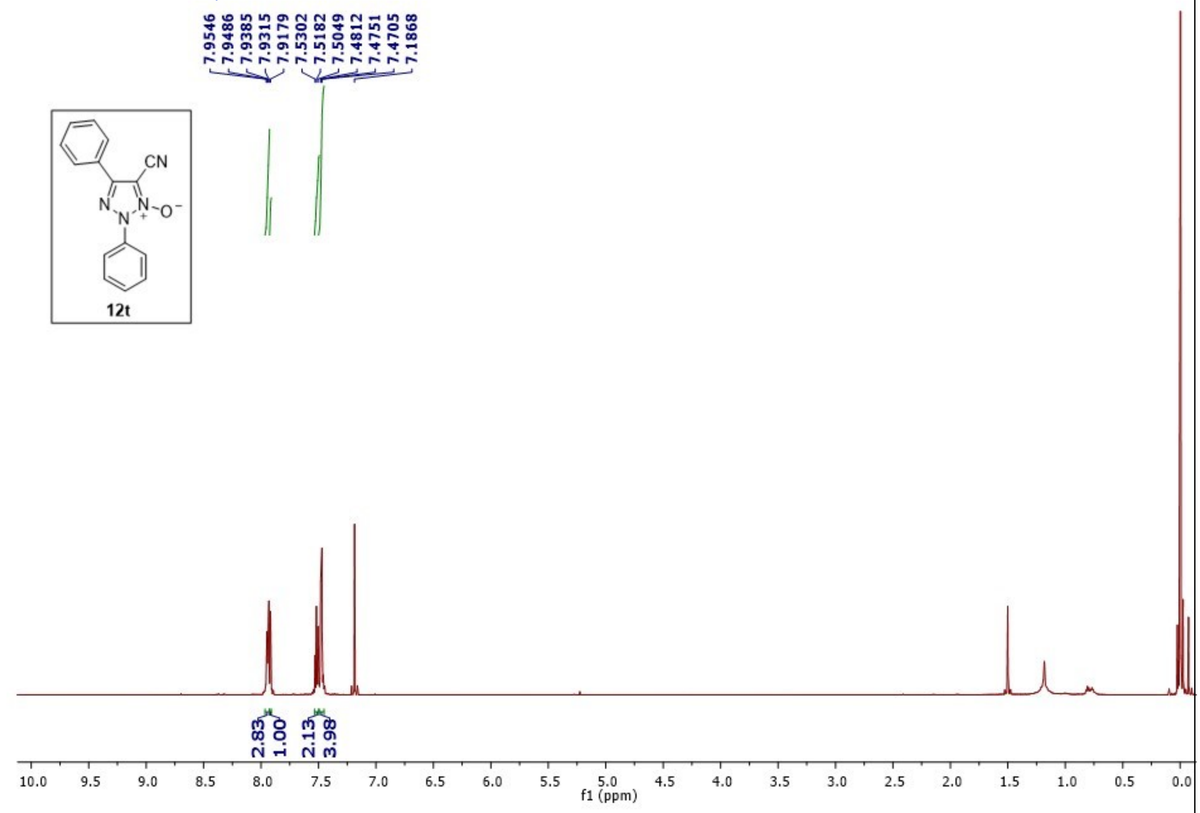

^{13}C NMR of Compound 12t

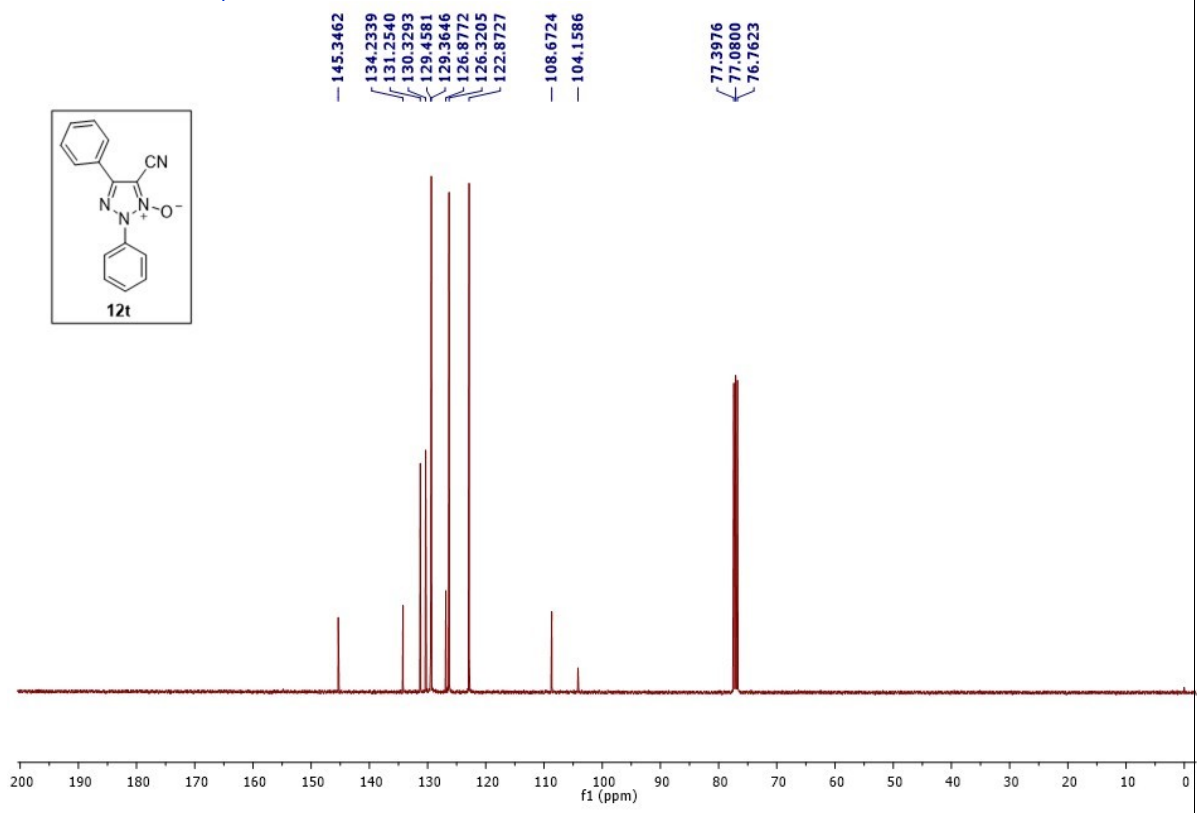

HRMS of Compound 12t

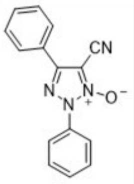

12t

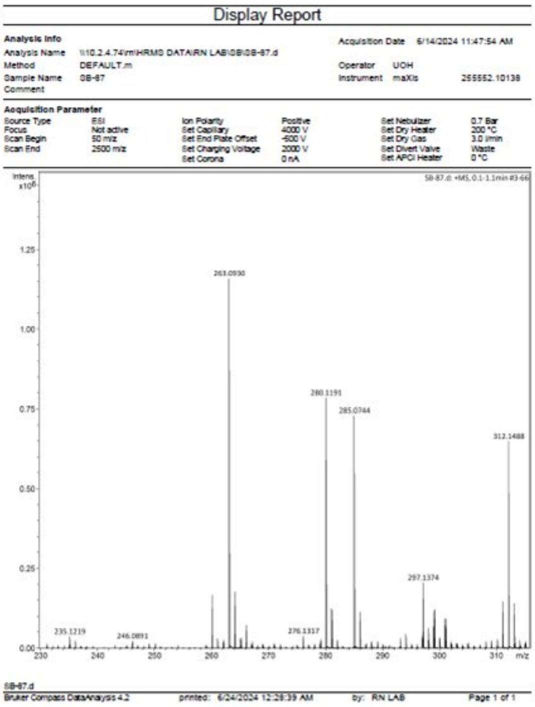

^{1}H NMR of Compound 12u

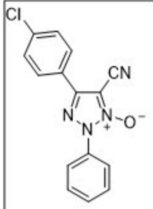

12u

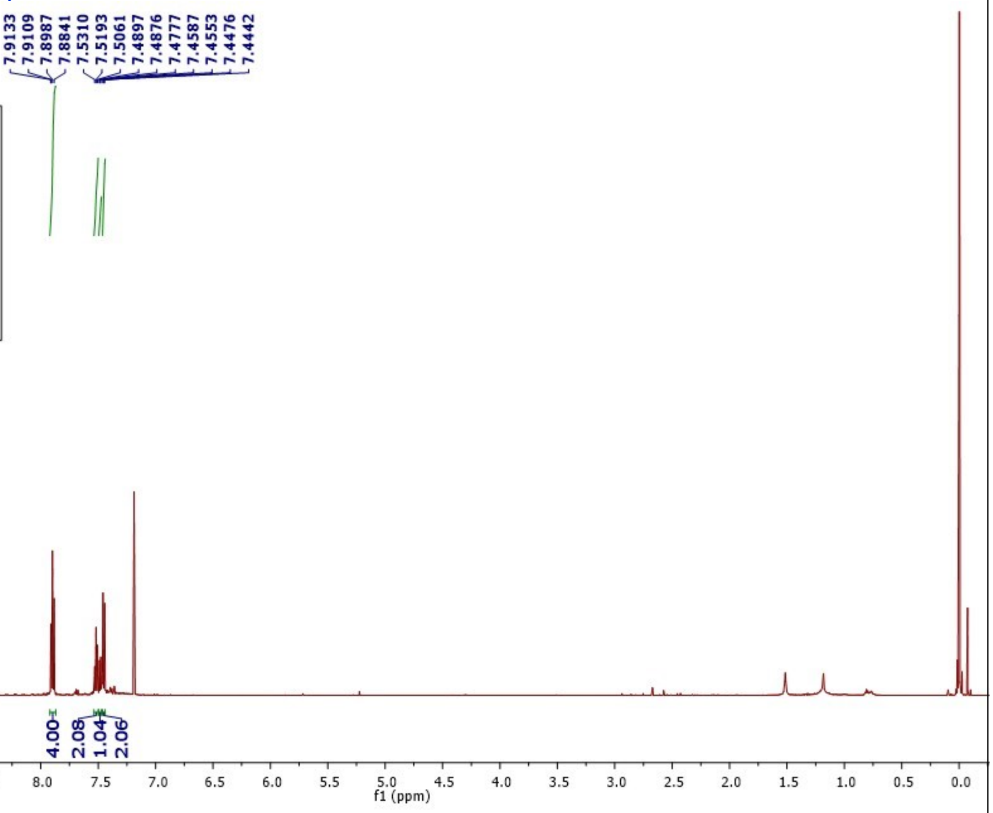

^{13}C NMR of Compound 12u

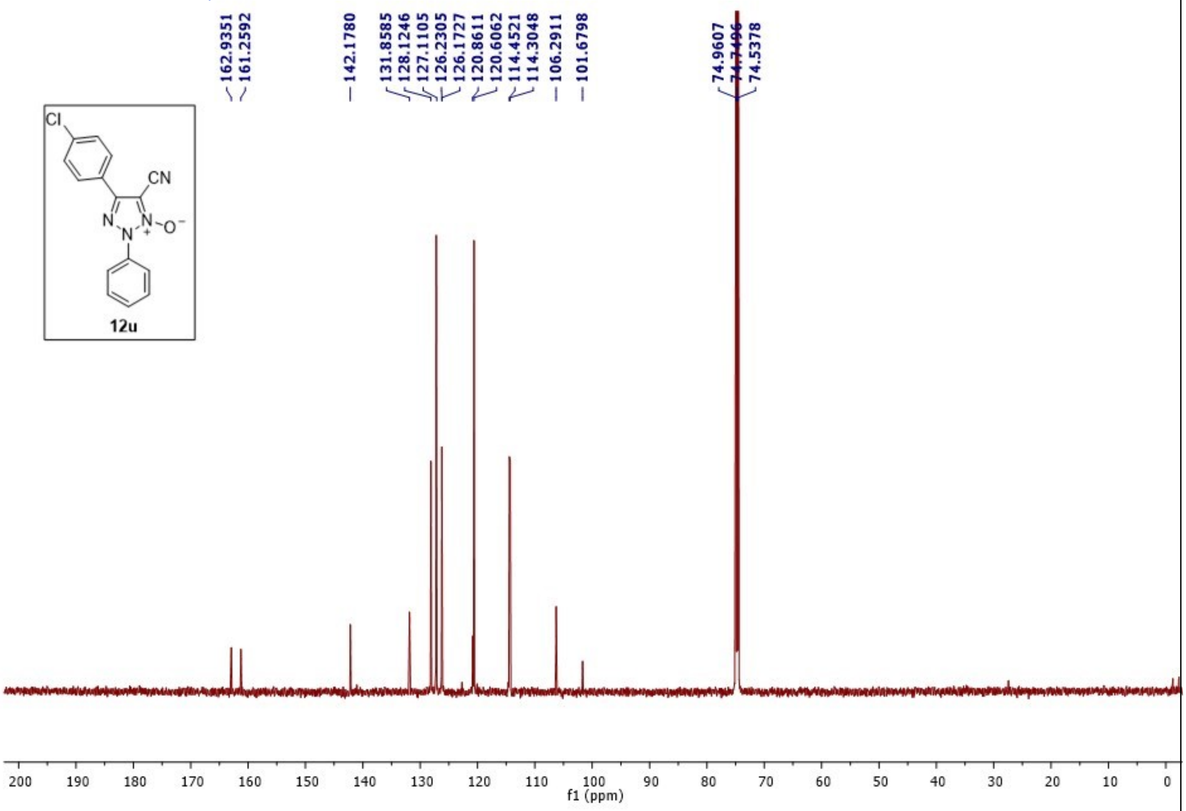

HRMS of compund 12u

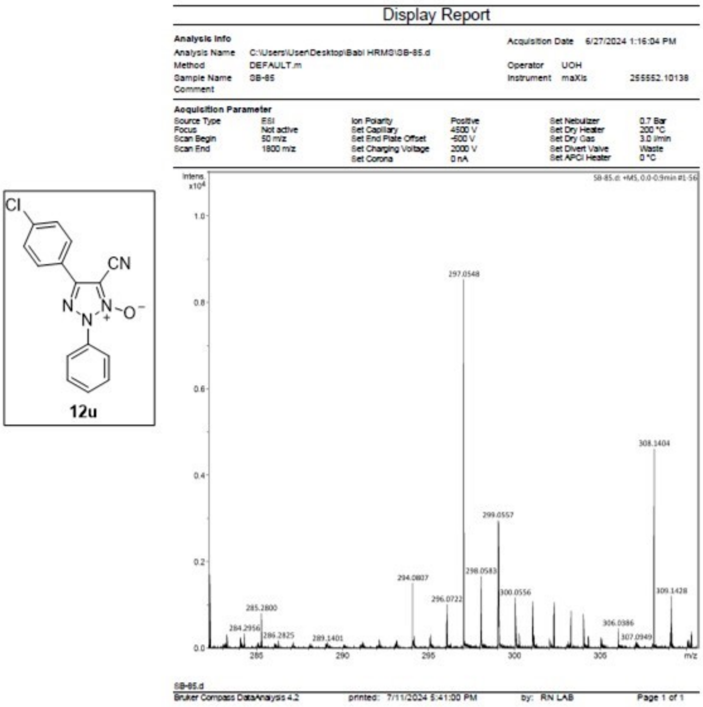

^{1}H NMR of compound 12v

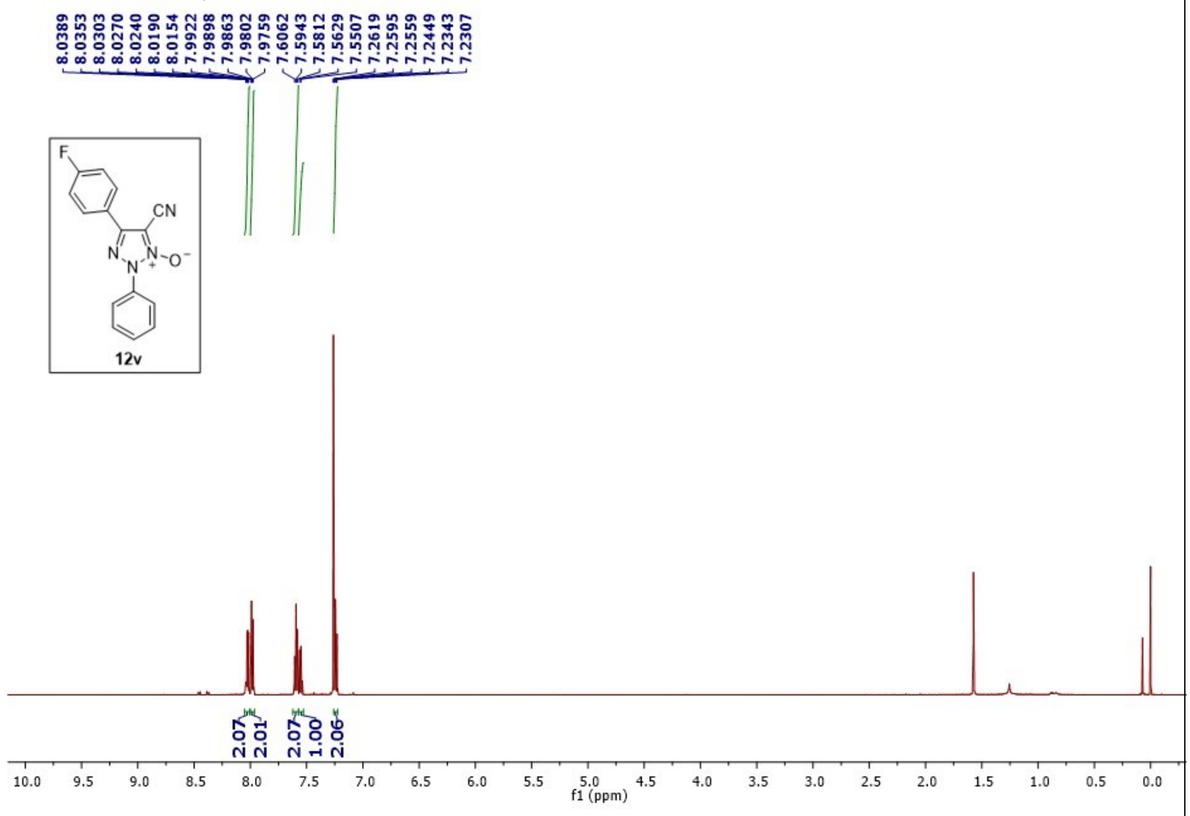

^{13}C NMR of compound 12v

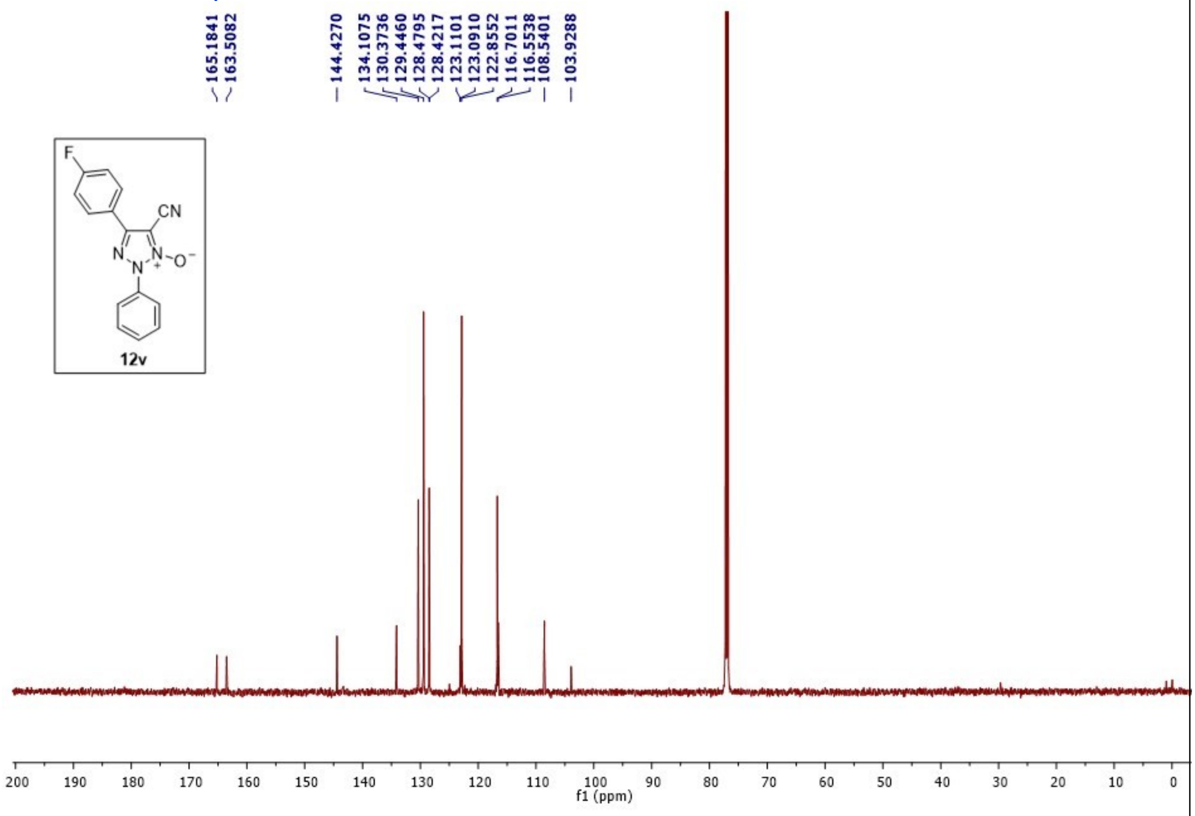

HRMS of compound 12v

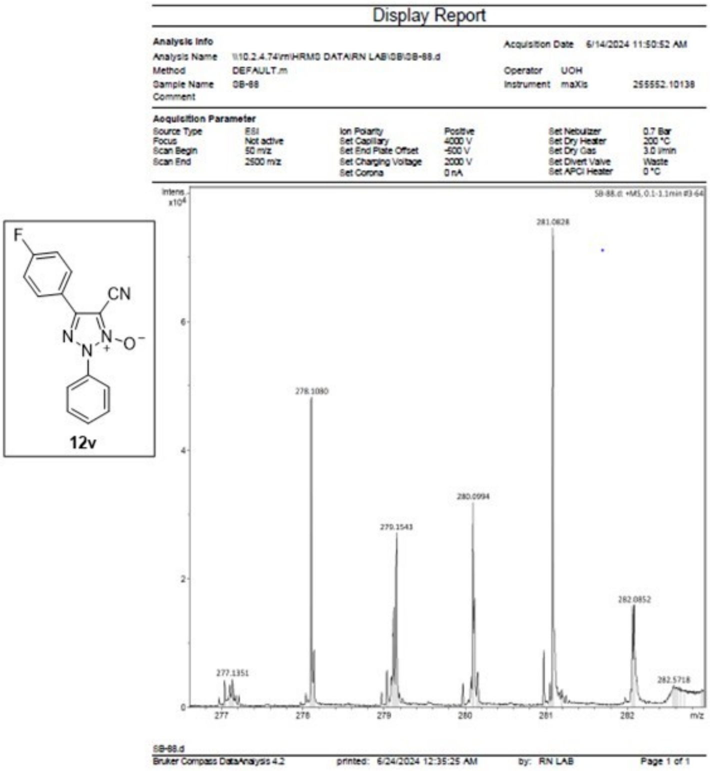

¹H NMR of compound 13C

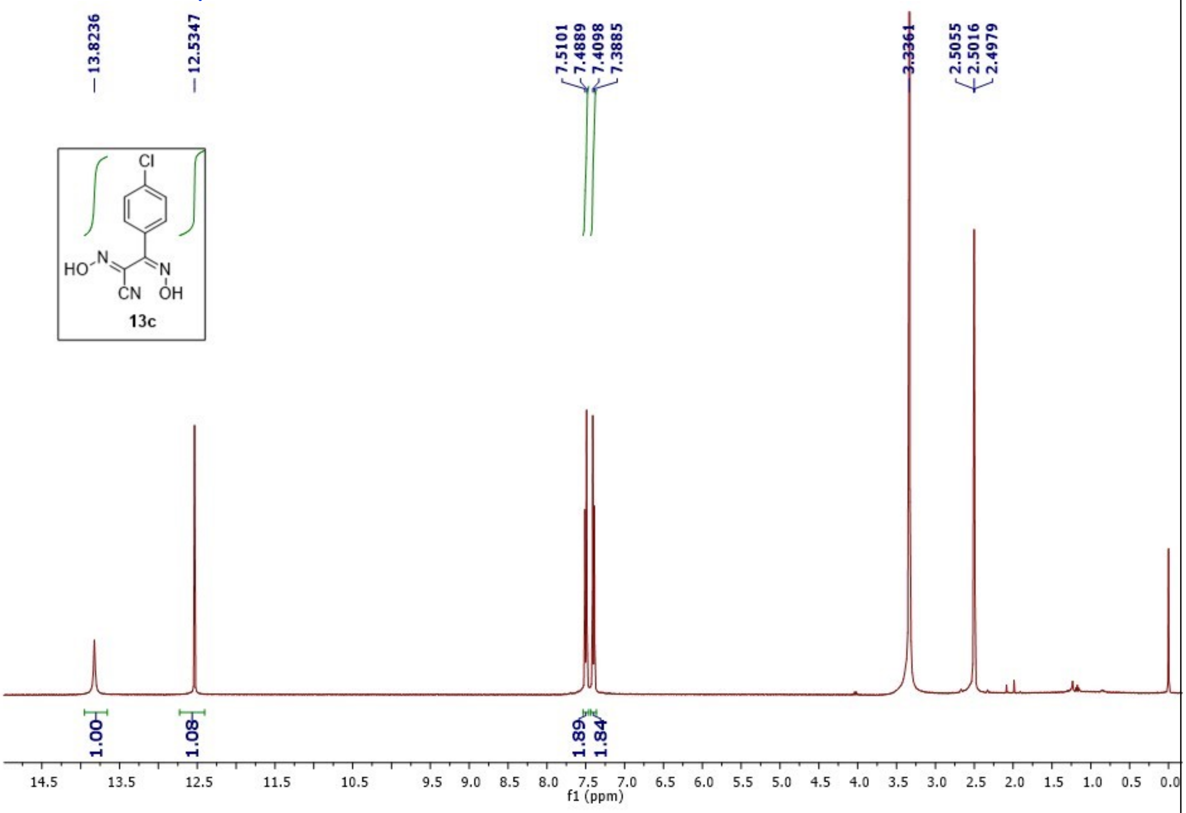

^{13}C NMR of Compound 13C

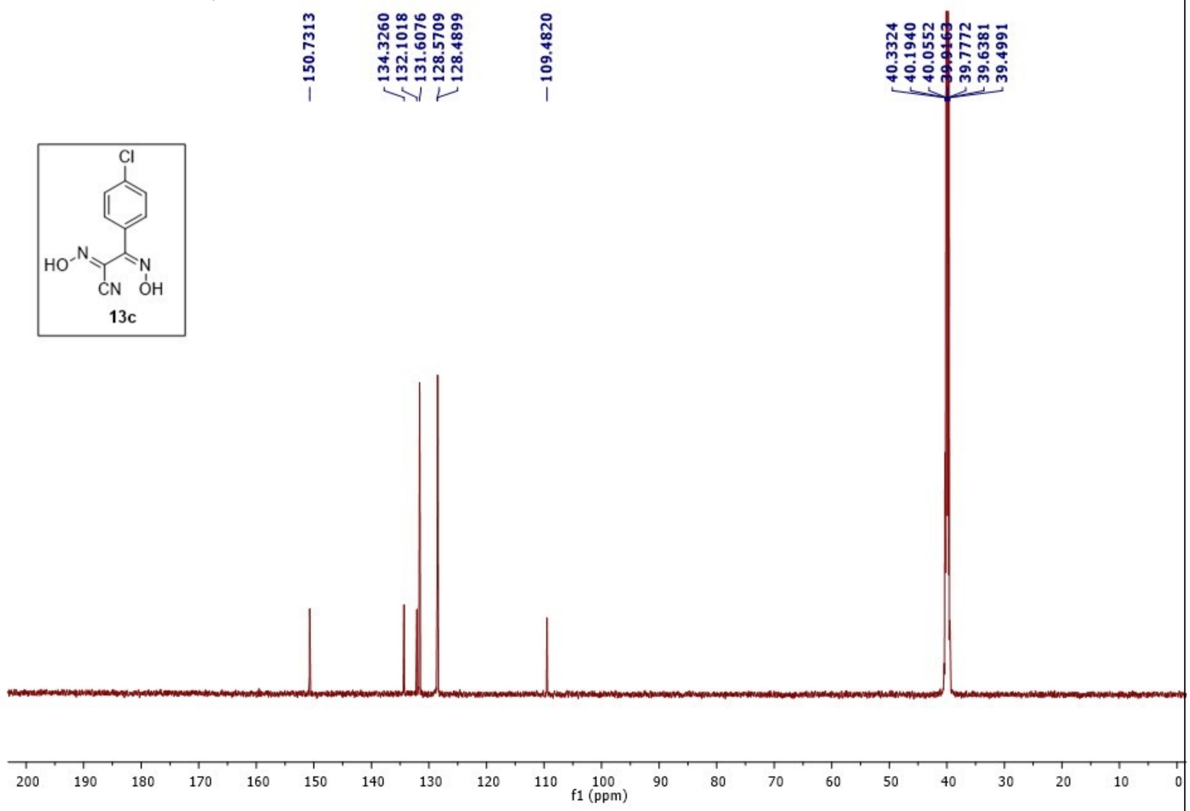

HRMS of Compound 13C

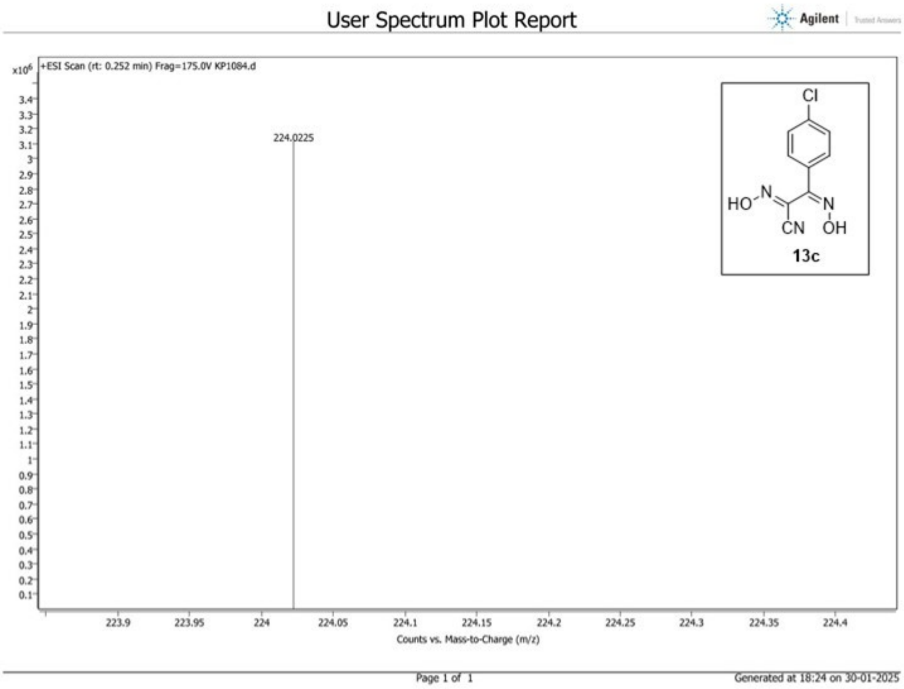

^{1}H NMR of Compound 14a

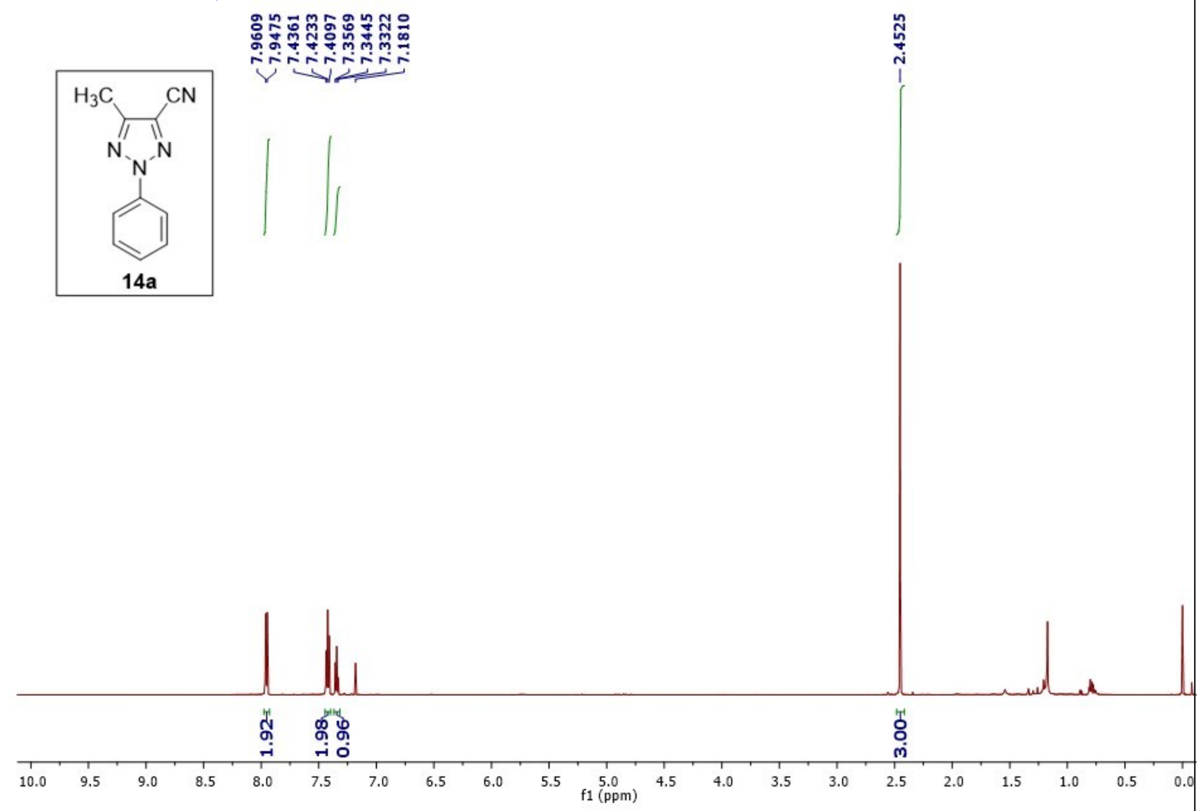

^{13}C NMR of compound 14a

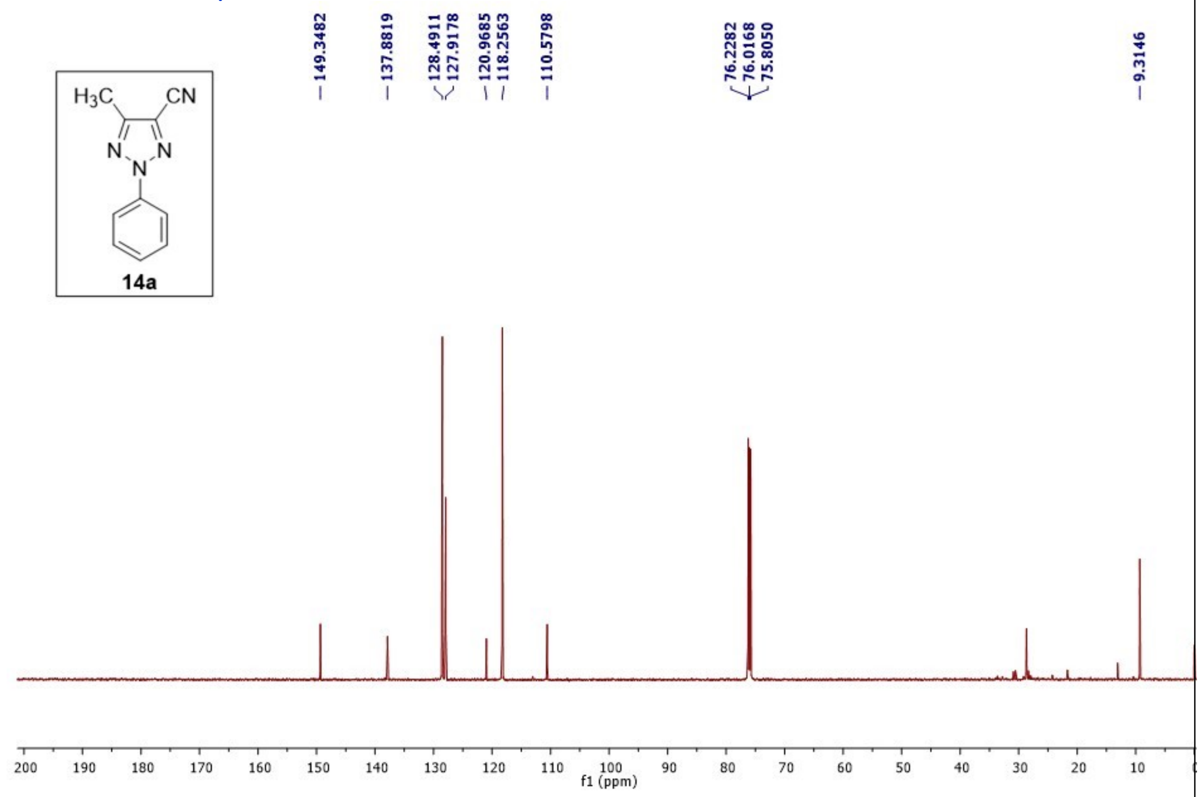

HRMS of Compound 14a

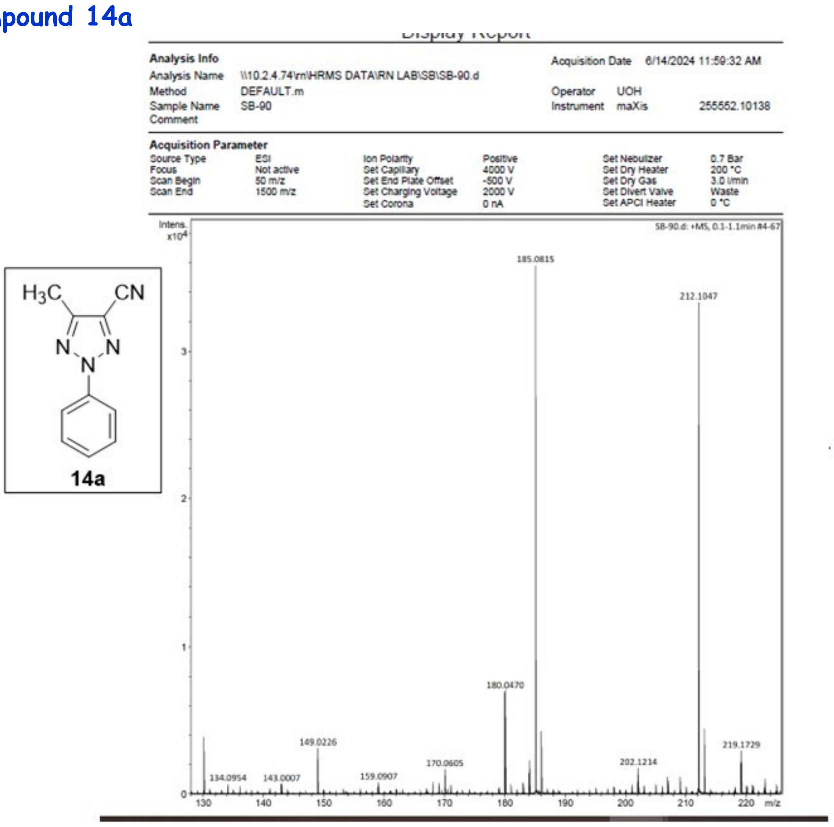

^{1}H NMR of Compound 14b

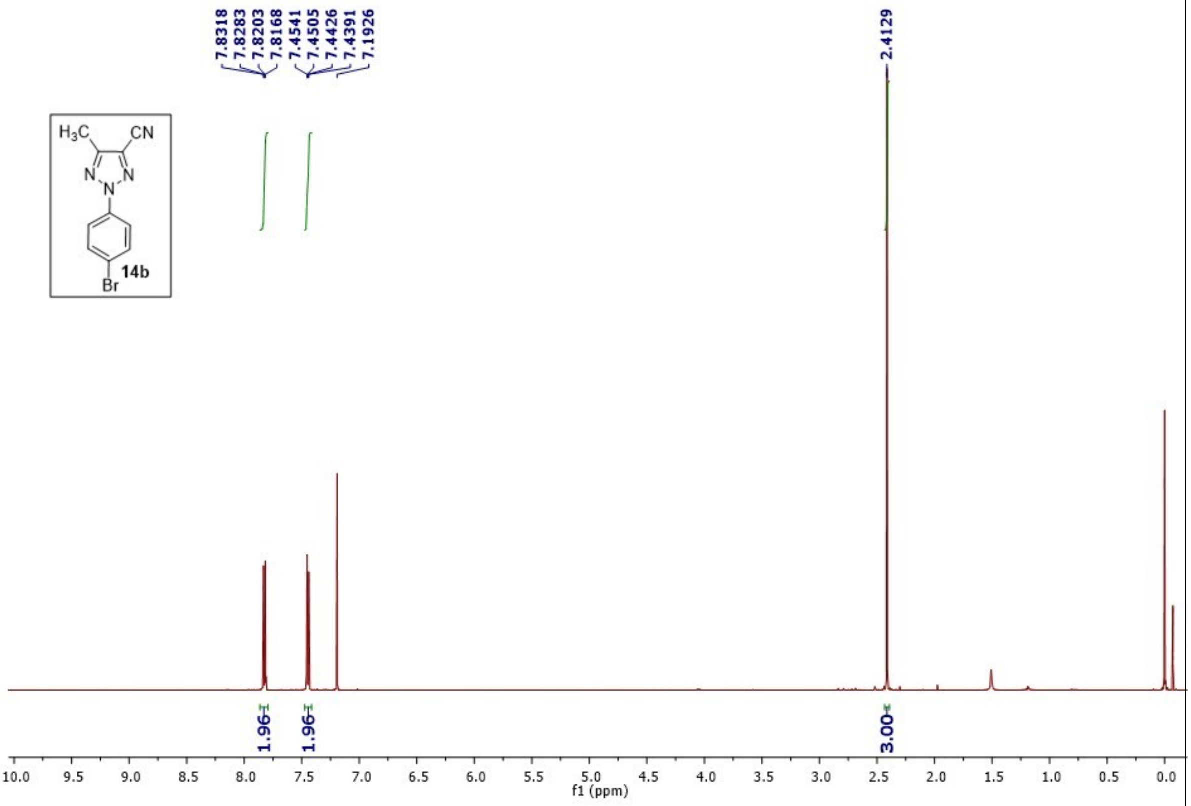

^{13}C NMR of Compound 14b

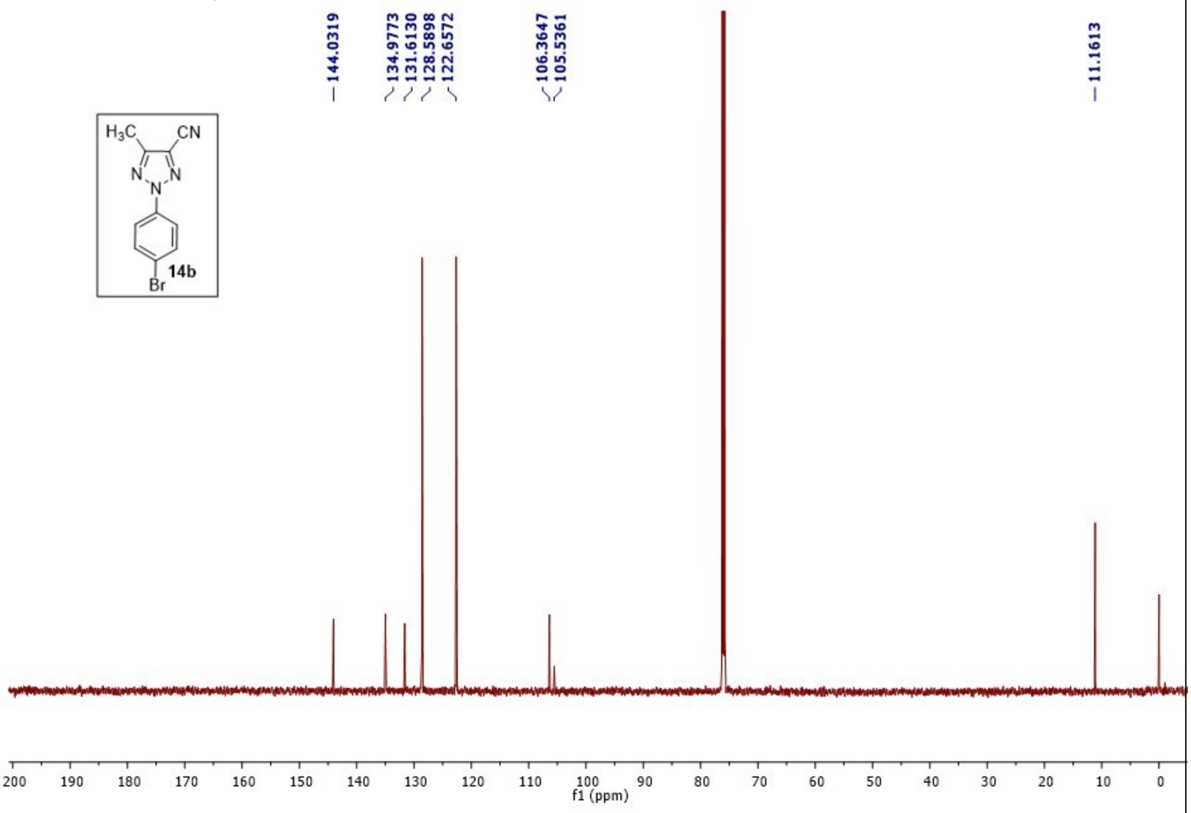

HRMS of Compound 14b

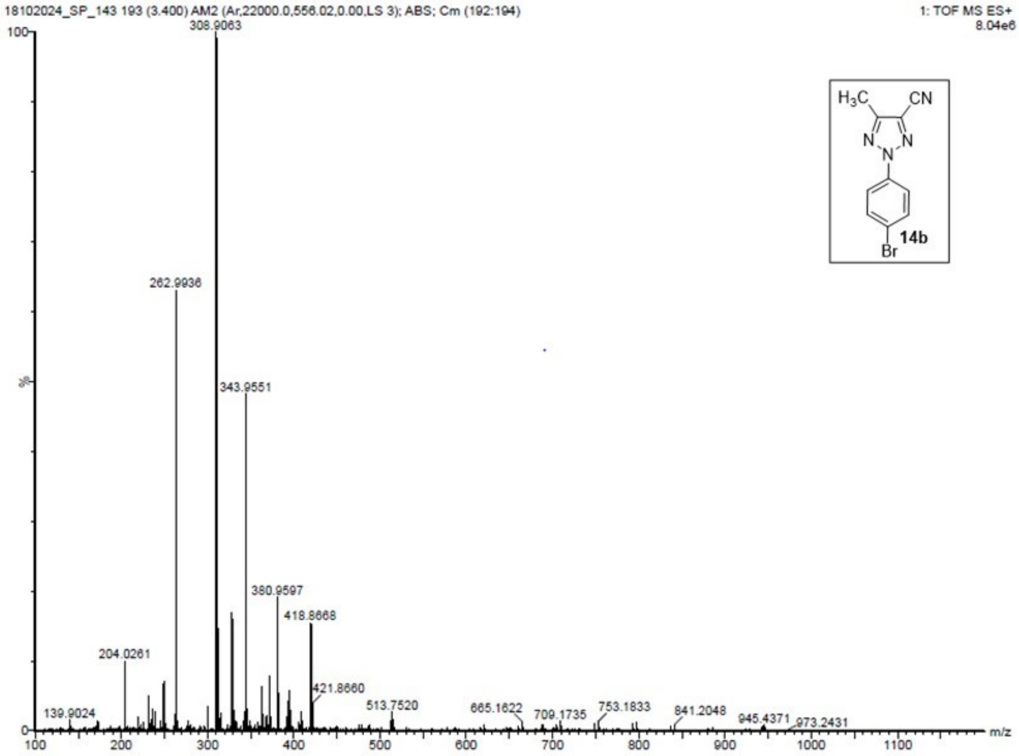

^{1}H NMR of Compound 14c

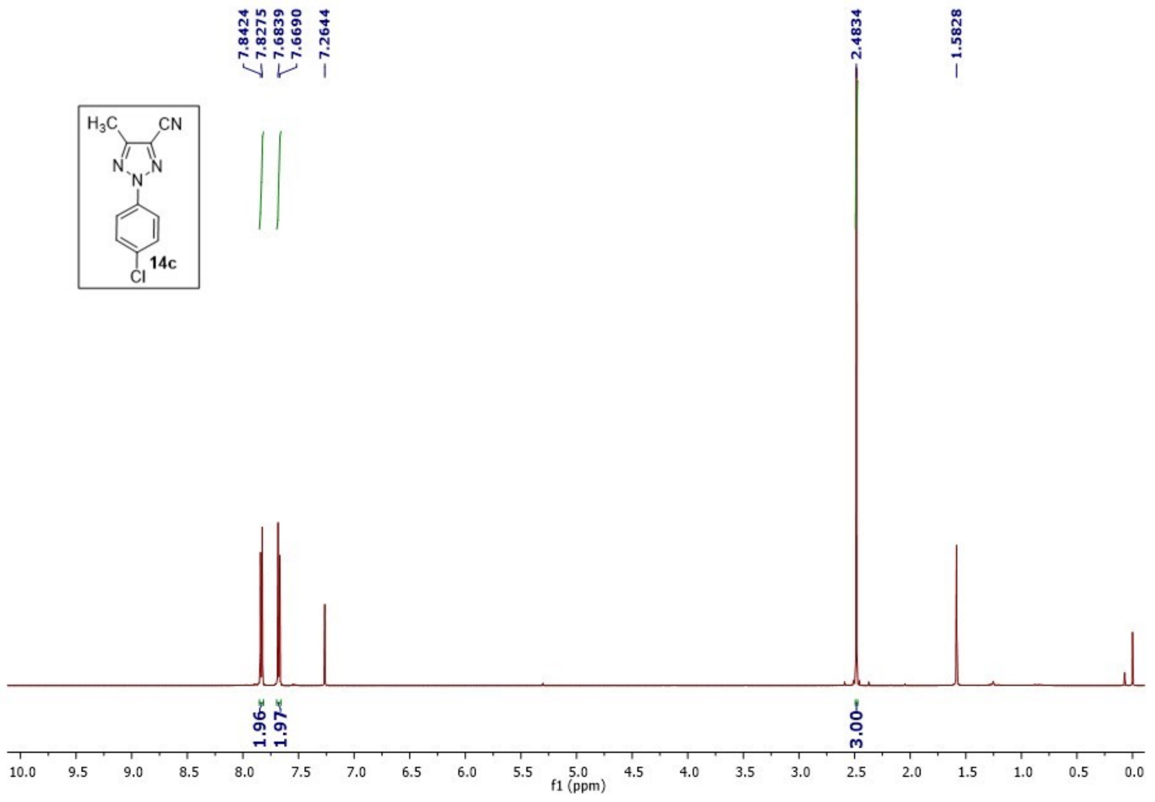

^{13}C NMR of Compound 14c

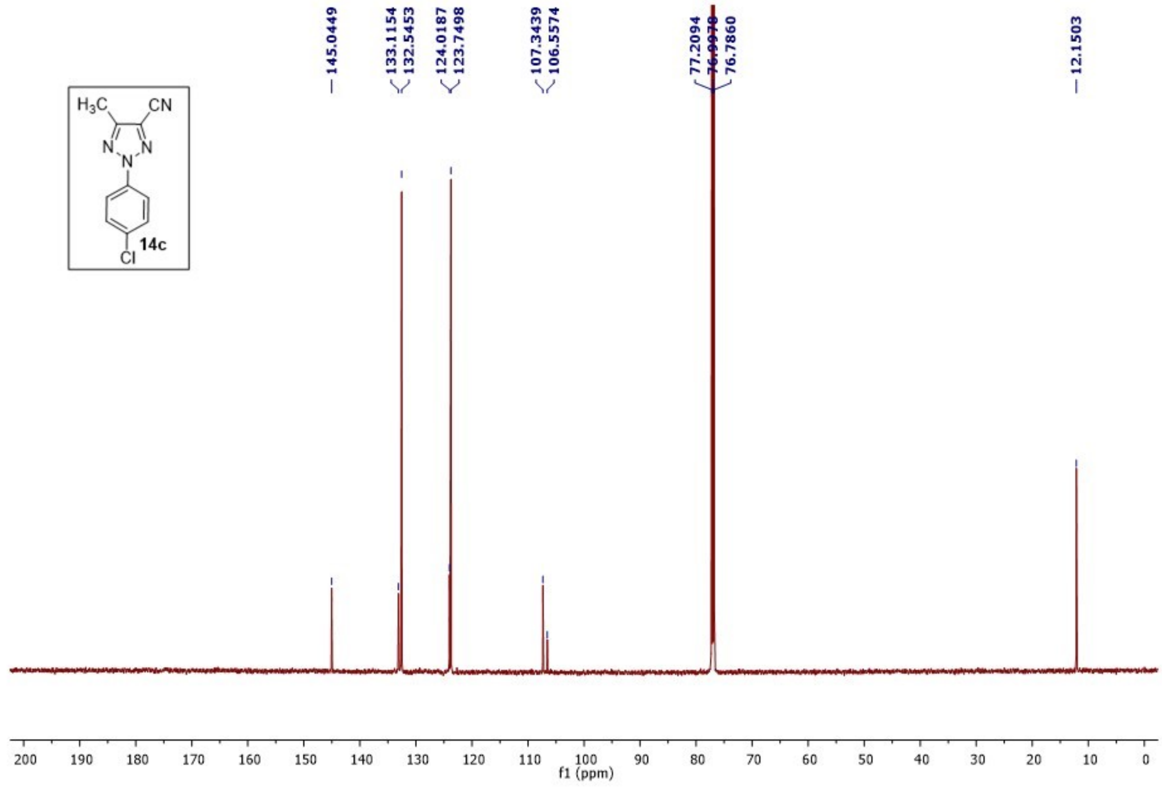

HRMS of Compound 14c

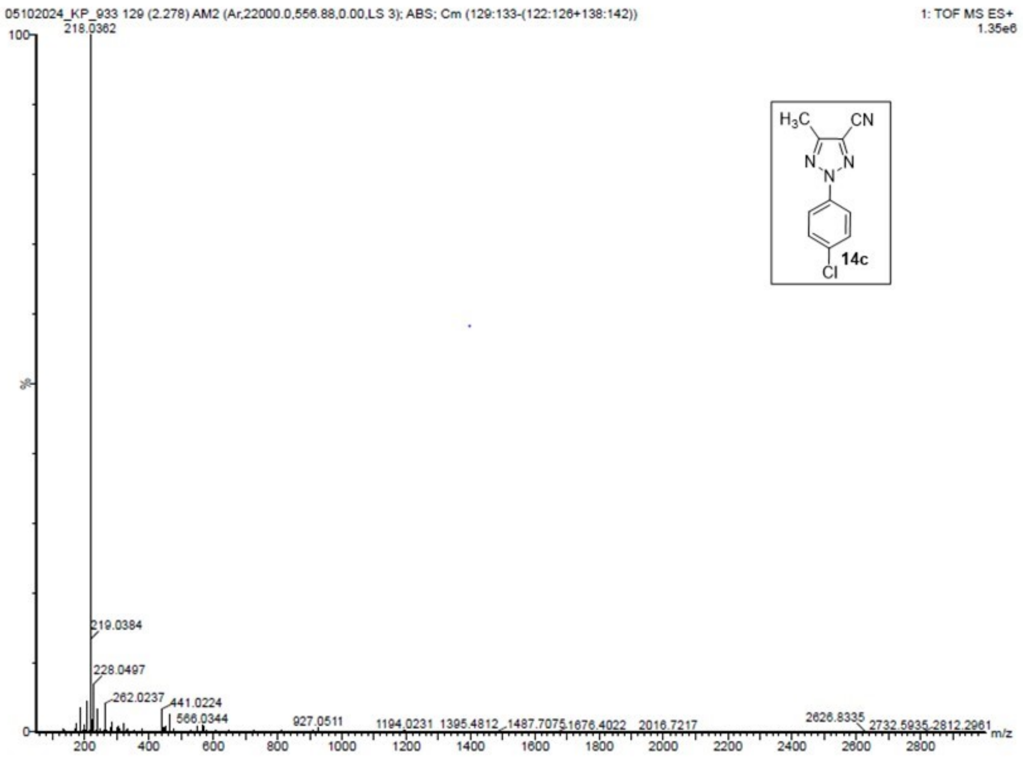

^{1}H NMR of Compound 14d

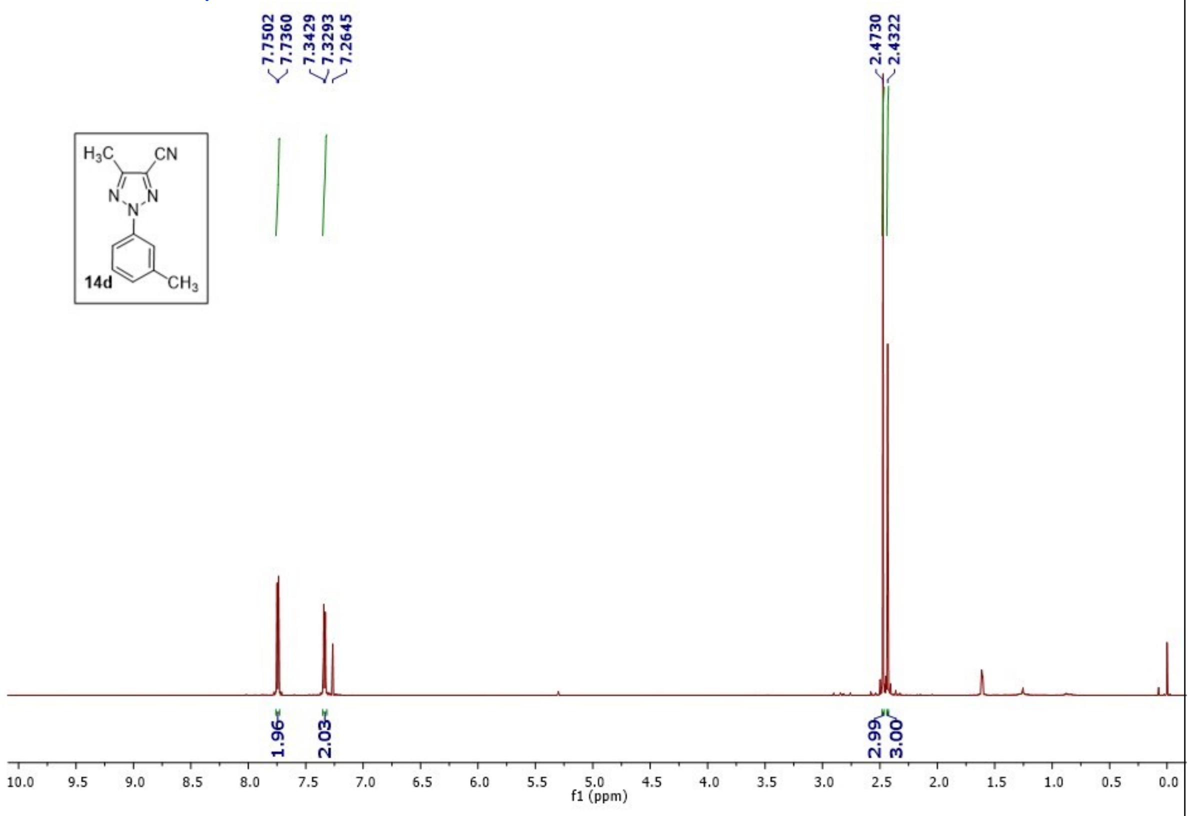

^{13}C NMR of Compound 14d

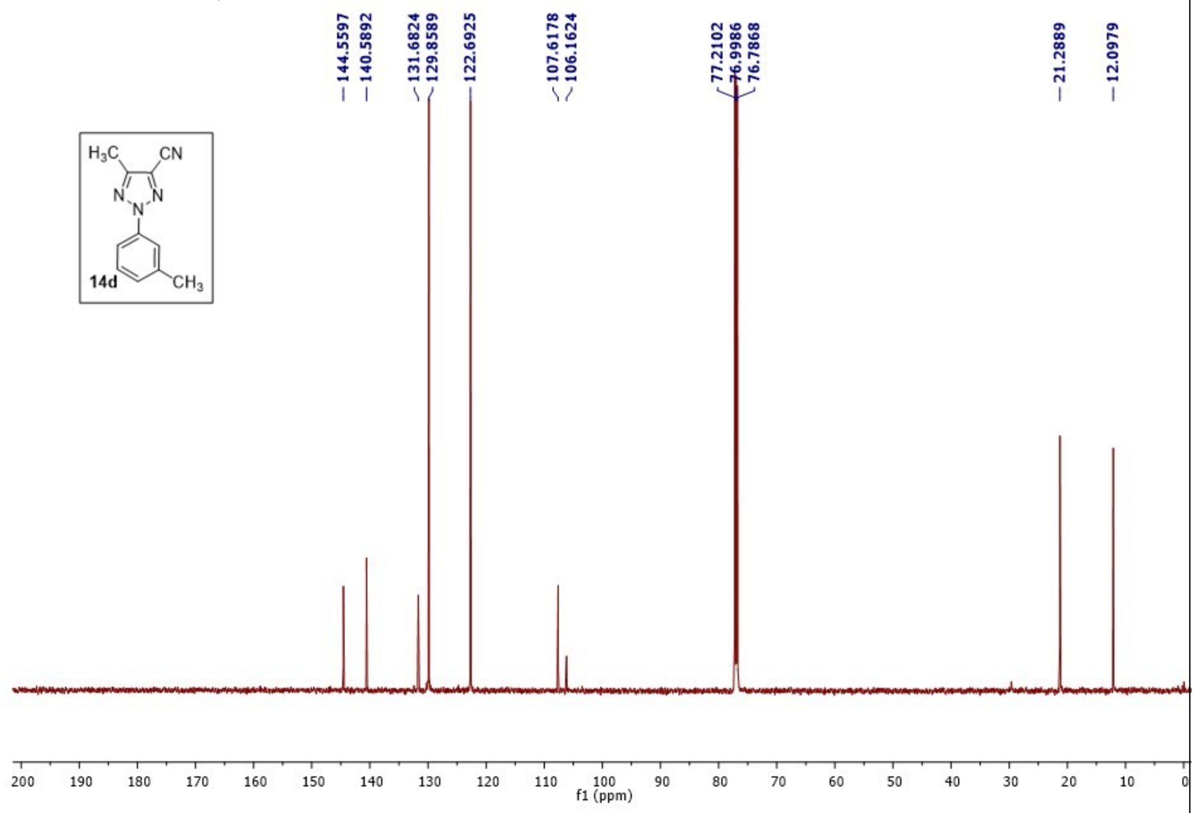

HRMS of Compound 14d

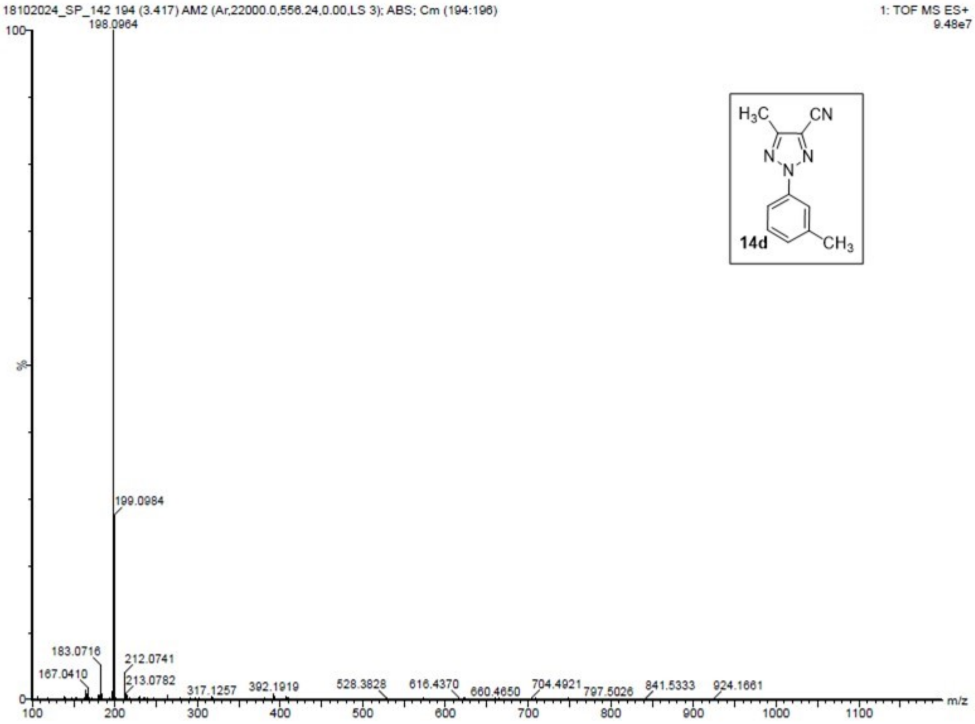

^{1}H NMR of Compound 14e

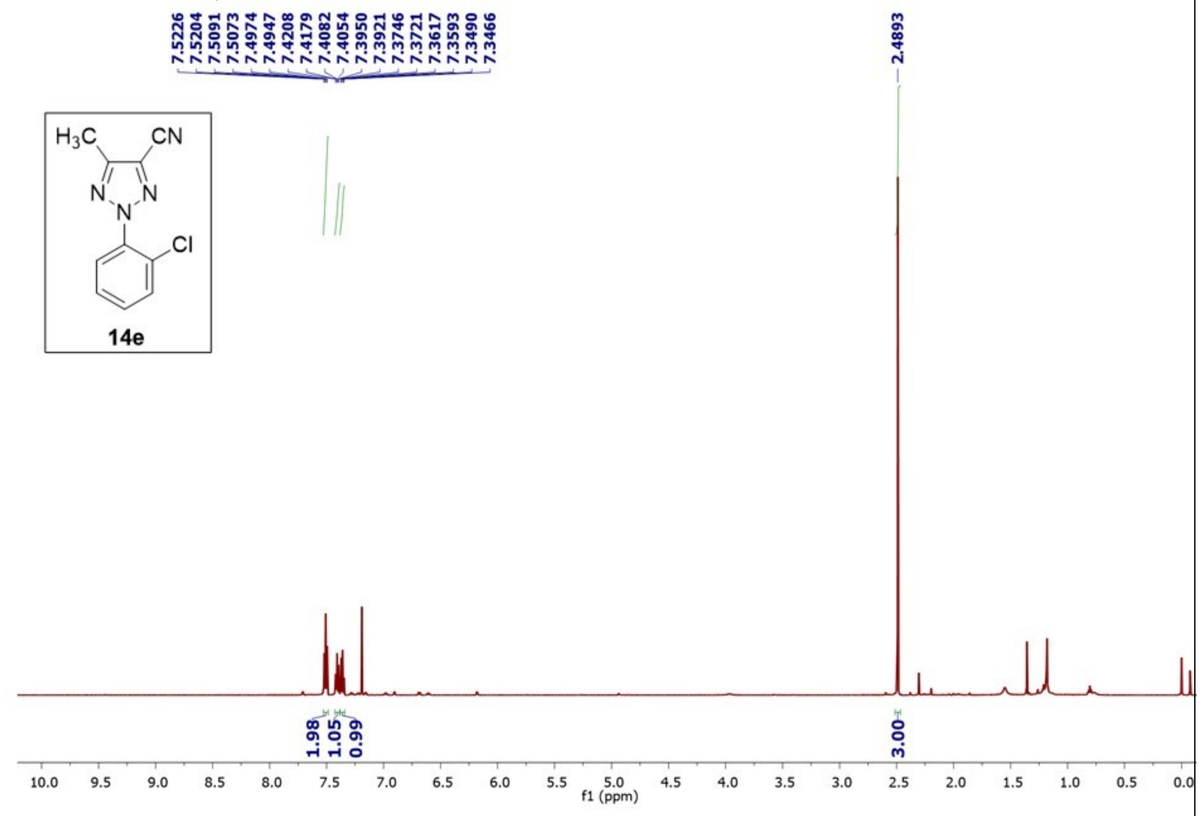

^{13}C NMR of Compound 14e

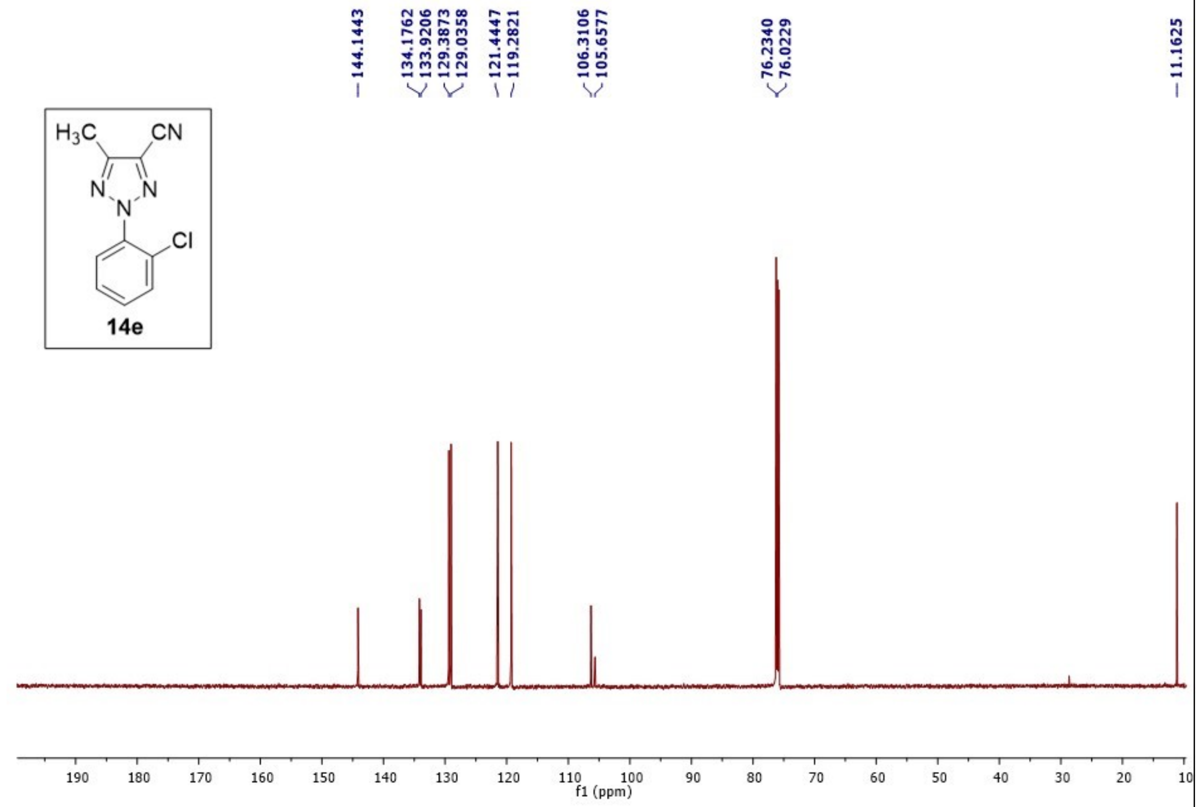

HRMS of Compound 14e

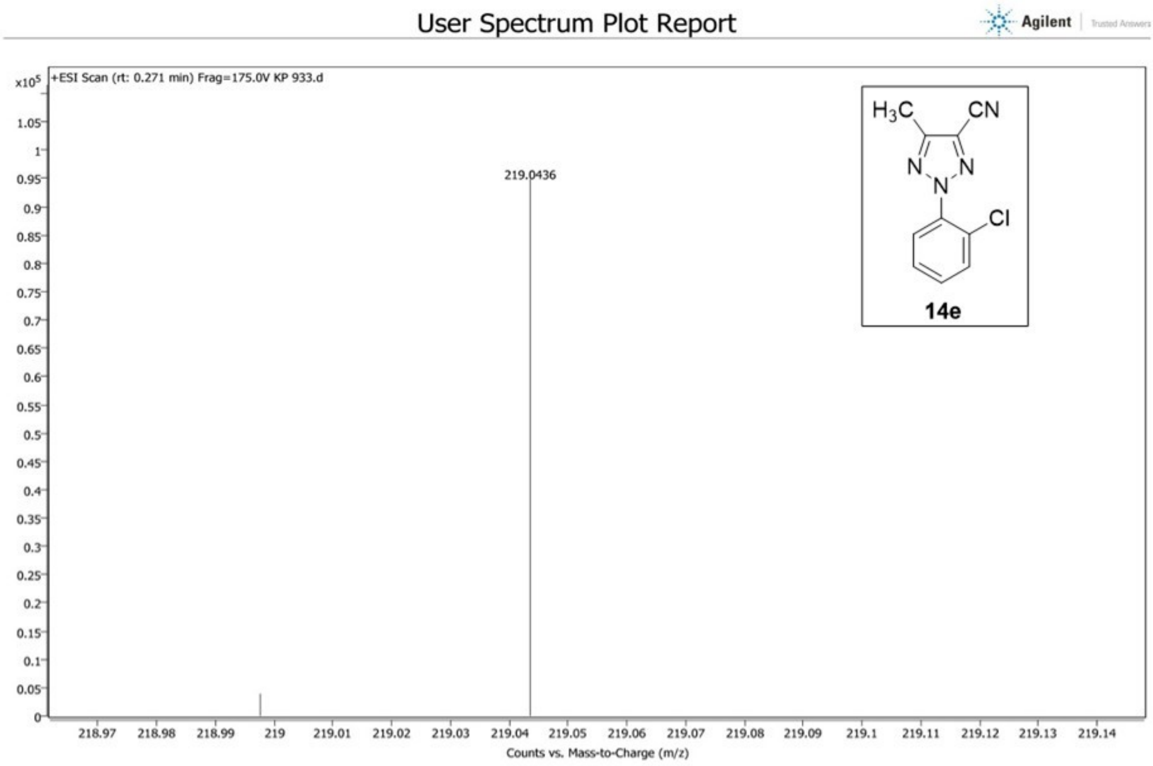

^{1}H NMR of Compound 16a

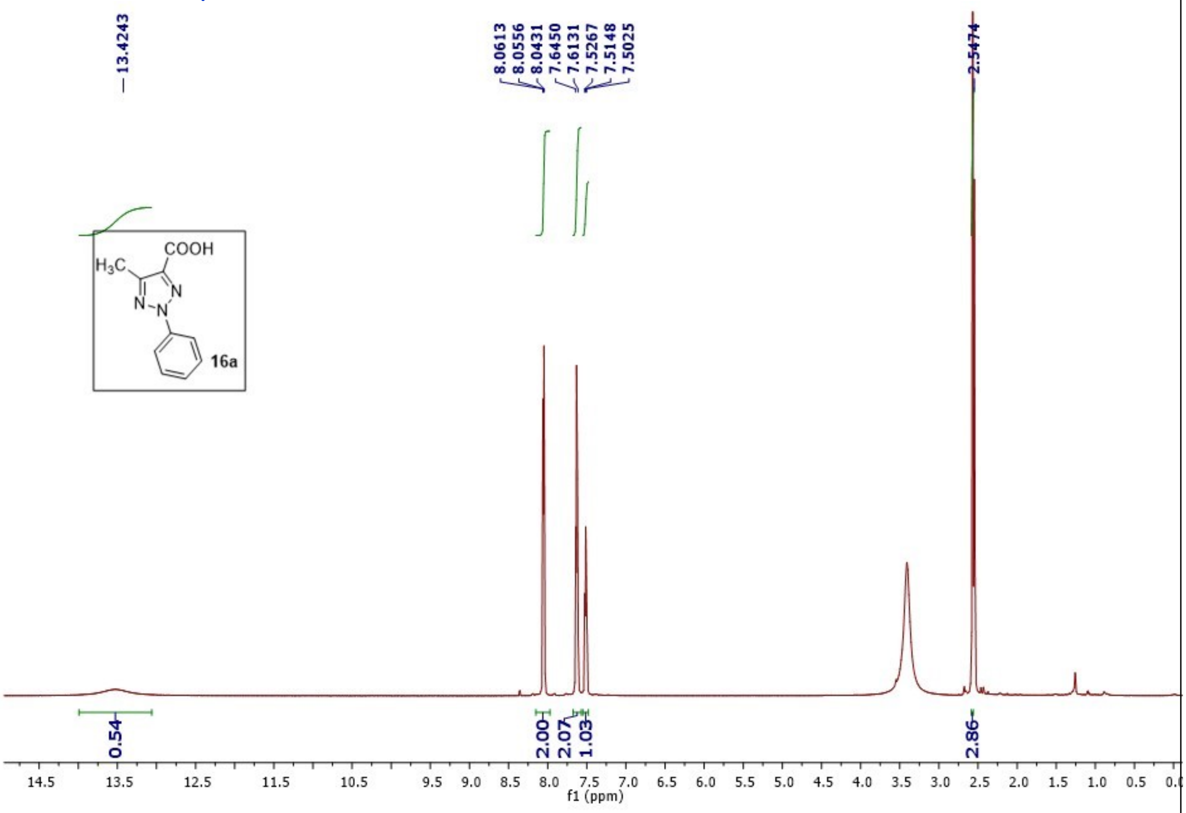

^{13}C NMR of Compound 16a

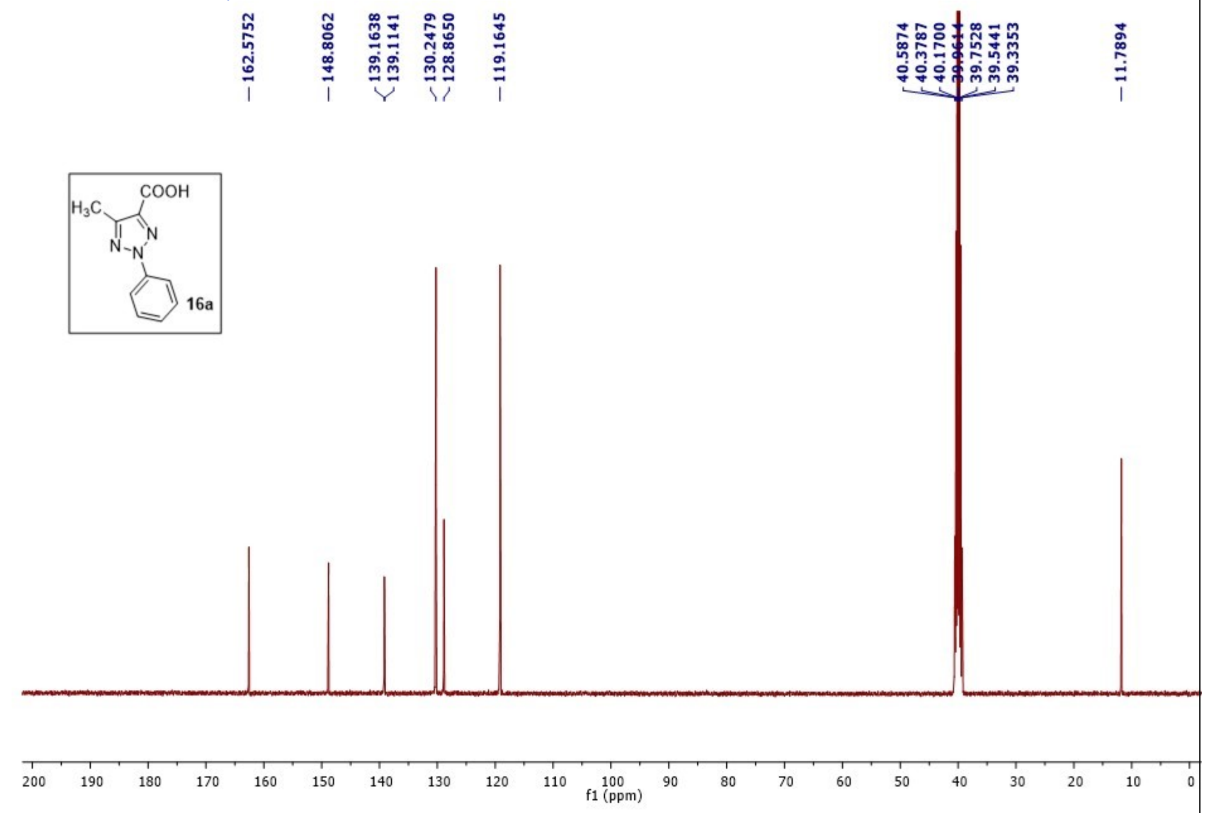

^{1}H NMR of Compound 16b

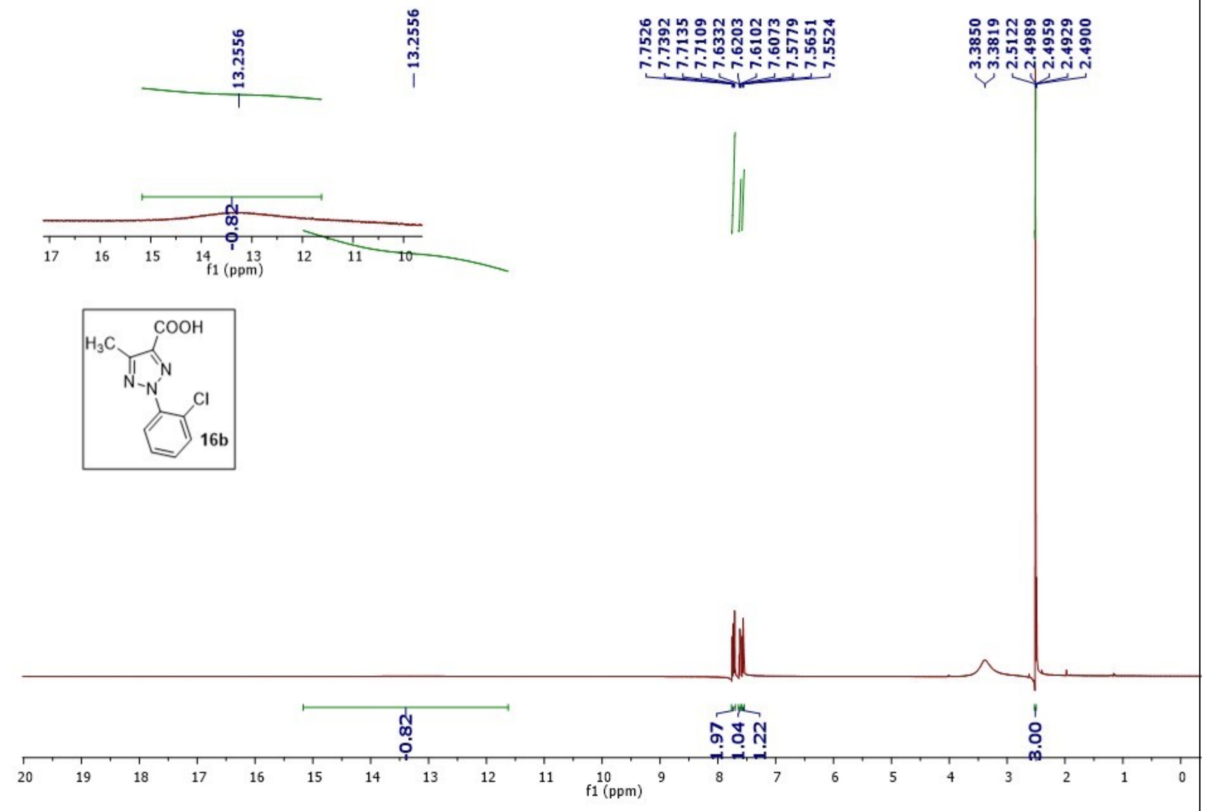

^{13}C NMR of Compound 16b

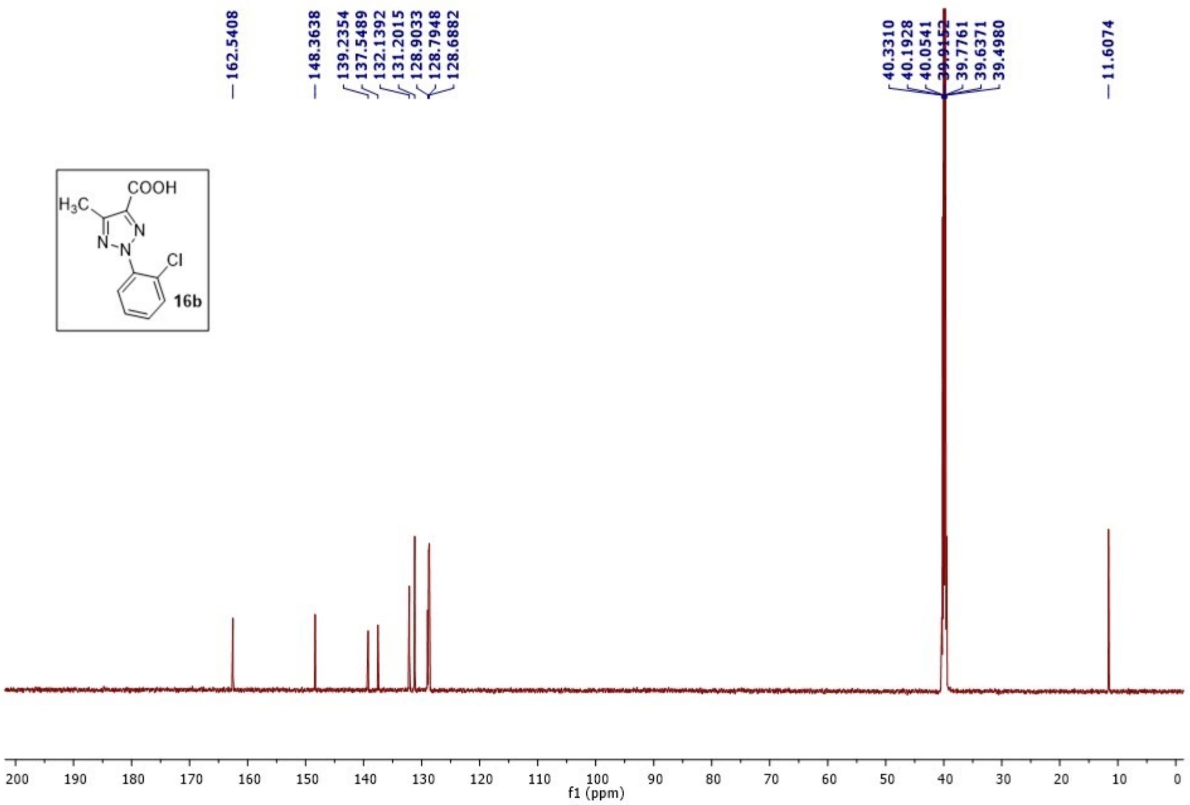

Supplement: RA-015-D5RA01327E-s001 [file RA-015-D5RA01327E-s001.pdf]
